# Overexpression of DDX49 in prostate cancer is associated with poor prognosis

**DOI:** 10.1186/s12894-023-01251-4

**Published:** 2023-04-27

**Authors:** Junyue Tao, Qintao Ge, Jialing Meng, Chaozhao Liang, Zongyao Hao, Jun Zhou

**Affiliations:** 1 Department of Urology, The First Affiliated Hospital of Anhui Medical University, Jixi Road 218, Shushan District, Hefei City, 230022 Anhui Province People’s Republic of China; 2 Institute of Urology, Anhui Medical University, Hefei, 230032 People’s Republic of China; 3 Anhui Province Key Laboratory of Genitourinary Diseases, Anhui Medical University, Hefei, 230032 People’s Republic of China

**Keywords:** DDX49, Prostate cancer, Prognosis, Proliferation, Apoptosis

## Abstract

**Background:**

There is increasing evidence that DEAD-box helicases (*DDX*) can act either as promoters or suppressors in various cancer types. Nevertheless, the function of *DDX49* in prostate cancer (PCa) is unknown. This study reveals the prognostic and predictive value of *DDX49* in PCa.

**Methods:**

First, we evaluated the expression of *DDX49* between PCa and normal tissues based on TCGA and GEO databases. Univariate and multivariate regression analyses were conducted to reveal the risk factors for PCa recurrence. A K–M curve was employed to assess the relationship between *DDX49* and recurrence-free survival. In vitro, *DDX49* expression was evaluated in PCa and normal prostate cell lines. Furthermore, we constructed a sh*DDX49* lentivirus to knock down the expression of *DDX49*. Celigo^®^ Image Cytometer and MTT assay were performed to analyse cell proliferation in PC-3 cells. Cell cycle distribution was detected with flow cytometry analysis. Apoptosis affected by the lack of *DDX49* was metred with the PathScan^®^ Stress and Apoptosis Signalling Antibody Array Kit.

**Results:**

This study shows a high increase in *DDX49* in PCa tissues in comparison with normal tissues and that increased *DDX49* indicates a poor prognosis among PCa patients. Meanwhile, *DDX49* knockdown suppressed the proliferation and migration of PC-3 cells, causing cell cycle arrest in the G1 phase. Stress and apoptosis pathway analysis revealed that the phosphorylation of HSP27, p53, and SAPK/JNK was reduced in the *DDX49* knockdown group compared with the control group.

**Conclusions:**

In summary, these results suggest that high expression of *DDX49* predicts a poor prognosis among PCa patients. Downregulation of *DDX49* can suppress cell proliferation, block the cell cycle, and facilitate cell apoptosis. Therefore, knockdown of *DDX49* is a promising novel therapy for treating patients with PCa.

**Supplementary Information:**

The online version contains supplementary material available at 10.1186/s12894-023-01251-4.

## Background

Prostate cancer (PCa) is among the dominant causes of cancer deaths in men worldwide [1]. Androgen deprivation therapy (ADT) with drugs or surgical castration has become the main therapy for metastatic prostate cancer in recent years [2, 3]. Although ADT works initially, men with clinically advanced PCa usually develop castration-resistant prostate cancer (CRPC) within a few years after ADT [4]. Genome-wide association studies (GWAS) have validated that the heterogeneity of genetic alterations in CRPC patients plays an essential role [5]. Therefore, studying the role of genetic alterations in PCa and searching for new molecular markers may provide important references for exploring the underlying mechanism of PCa and developing more effective treatments for CRPC.

DEAD (Asp-Glu-Ala-Asp)-box family genes exert a vital function in the metabolism and functional activation of RNAs [6]. Previous reports have indicated that several members of the DEAD-box family showed aberrant expression in many cancers, and deregulated expression of these RNA helicase-regulating genes could affect cell proliferation and apoptosis, thereby promoting carcinogenesis or cancer progression [7–9]. Tanaka et al. reported that *DDX1* was involved in the initial progression of testicular tumours through the regulation of *cyclin-D2*, *CD9* and *NANOG* [10]. Furthermore, George et al. found that *DDX1* promotes tumorigenesis and that *DDX1* is associated with the high expression of *MYCN* in advanced neuroblastoma, which indicates a poor prognosis [11]. Overexpression of *DDX9* has been reported among many cancers, and elevated *DDX9* may promote Wilms tumour metastasis, contribute to the progression of colorectal cancer through the activation of the NF-κB signalling pathway, and be related to the poor clinical outcome of lung cancer [12–14].

Furthermore, several studies have also investigated the roles of *DDX* in PCa. Clark et al. found that DDX5 is overexpressed in PCa compared to normal prostate tissue, and *DDX5* expression is positively correlated with the stage of PCa. More importantly, they demonstrated a possible functional role for *DDX5* as an androgen receptor (*AR*) transcriptional coactivator and mediator of posttranscriptional regulation of *AR* mRNA [15]. In another study, *DDX3* also regulated *AR* posttranscriptionally by sequestering mRNA, and inhibiting *DDX3* was sufficient to reduce AR protein expression and signalling, resensitizing PCa cells to AR signalling inhibitors [16]. Yu et al. [17] reported that *DDX52* knockdown inhibits PCa cell growth by regulating c-Myc signal transduction, and they found that *DDX52* has a significant decreasing effect on c-Myc, which promotes the progression of PCa and that c-Myc’s oncogenic potential in PCa cells may rely on DDX52. However, the DDX family comprises at least 35 genes, most of which have not been evaluated for function in PCa, especially in castration-resistant PCa. It is important to understand how these genetic changes contribute to the progression of PCa, as they will contribute to the study of the mechanism and treatment of PCa.

Here, we reveal the association of *DDX49* and PCa prognosis in the Cancer Genome Atlas (TCGA) as well as the Gene Expression Omnibus (GEO) database. Then, lentivirus-delivered short hairpin RNA (shRNA) was applied to knock down *DDX49* in PCa cells, and changes in cell proliferation, the cell cycle and cell apoptosis were evaluated.

## Materials and methods

### In silico analysis of DDX49 expression

To explore the correlation between *DDX49* expression and PCa, six cohorts were enrolled from TCGA (https://portal.gdc.cancer.gov/repository) and GEO databases (https://www.ncbi.nlm.nih.gov/geo/), including TCGA-PRAD, GSE29079, GSE25183, GSE46602, GSE38241, and GSE69223. From these, the association between different *DDX49* expression levels and recurrence-free survival (RFS) in PCa patients was assessed. In addition, the correlation of *DDX49* expression with apoptosis-related genes was assessed based on TCGA cohorts via a visualization tool, GEPIA (http://gepia.cancer-pku.cn/) [18]. We also identified genes correlated with *DDX49* expression from the STRING database, which predicted protein–protein interactions [19]. Subsequently, all genes correlated with *DDX49* were analysed for cellular processes and pathways through Gene Ontology (GO) term enrichment and the Kyoto Encyclopedia of Genes and Genomes (KEGG) database.

### Pathway enrichment analyses

Patients in the TCGA-PRAD cohort were classified into high-*DDX49* and low-*DDX49* groups in accordance with the mean *DDX49* expression. The R package “limma” was employed for screening differentially expressed genes (DEGs) between groups with the absolute value of fold-change > 0.4 and *p* adjusted value < 0.01 as cut-offs [20]. GO and KEGG enrichment analyses were conducted with the R package “org.Hs.eg.db” and further annotated via the R package “clusterProfiler”. The top five GO terms and the top KEGG term were listed and visualized by the R package “enrichplot” [21, 22].

### Cell culture

We purchased human cell lines, including RWPE-1, PC-3, LNCaP, and C4-2, from the Shanghai Cell Bank (Shanghai, China). Keratinocyte serum-free medium (K-SFM) supplemented with human recombinant epidermal growth factor (EGF) and bovine pituitary extract (BPE) was adopted for culturing RWPE-1 cells. PC-3, LNCaP, and C4-2 cells were cultured with RPMI 1640 media, 10% foetal bovine serum (FBS), 1% L-glutamine, and 1% penicillin–streptomycin liquid (all from Gibco^®^, Shanghai, China), respectively. We cultivated cells at 37 °C and 5% CO_2_ under a humidified environment.

### Quantitative reverse transcription–polymerase chain reaction (qRT‒PCR)

TRIzol reagent (Invitrogen, Shanghai, China) was adopted for isolating total RNA from RWPE-1, PC-3, LNCaP, and C4-2 cells. In addition, the primers used to test the expression of *DDX49* were as follows; forward: 5′-ATGAGCACGAGGACTGGTC-3′ and reverse: 5′-GCGGCAAAGCGTTCTTTCT-3′; for the *GAPDH* control, forward: 5′-TGACTTCAACAGCGACACCCA-3′ and reverse: 5′-CACCCTGTTGCTGTAGCCAAA-3′. Thermocycling conditions included 94 °C for 3 min, then 22 cycles of 94 °C for 30 s, 55 °C for 30 s, and 72 °C for 30 s, and then 72 °C for 5 min. All results were evaluated three times.

### Construction of and infection with shDDX49 lentivirus

The *DDX49* ORF was targeted to express shRNA using pGCL-GFP-lentivirus (GenBank no. NM_003410) (shDDX49 lentivirus). A nontargeting sequence was used for the lentivirus-negative control (shCtrl) (Shanghai Genechem Co. Ltd, Shanghai, China). Experiment template: 5′-GCCTGAGAATGATCATGGA-3′.

We chose PC-3 as the candidate for treatment with shDDX49 lentivirus. According to the MOI of lentivirus, 5 µl of shDDX49 or shCtrl lentivirus was added to each well of PC-3 cells cultured in 6-well plates. After the first 72 h of infection, we observed green fluorescence.

### Cell growth assay

We adopted the Celigo^®^ Image Cytometer to automatically identify the intensity and distribution of fluorescence in the PC-3 cells after treatment with shCtrl or shDDX49 lentivirus. After treatment with lentivirus for ten days, PC-3 cells in different groups were inoculated on 96-well plates at 2000 cells per well, and in the following five days, Celigo^®^ Image Cytometers were adopted to record the cell status each day.

The MTT assay is another way to evaluate cell growth inhibition caused by the lack of *DDX49*. PC-3 cells in different treatment groups were inoculated on six 96-well plates at 2000 cells per well, and we collected and detected the activity of cells on the 1st, 2nd, 3rd, 4th, and 5th days. First, the cells were washed three times in cold PBS, and a 3-(4,5-dimethyl-2-yl)-2,5-diphenyltetrazolium bromide (MTT) solution was added to every well to reach a final concentration of 0.5 mg/ml. Then, the cells were cultured in MTT solution at 37 °C for 4 h, and the solution was discarded. Finally, formazan salt was solubilized in 100 ml of dimethyl sulfoxide (DMSO) for 10 min. In addition, the OD was measured at 490 nm per well.

### Cell cycle assay

We employed the flow cytometry system to assess the different proportions of cell cycle stages among the shCtrl and shDDX49 groups. After treatment with different lentiviruses, we collected the cells at 1, 2, 3, 4, or 5 days. Cells were rinsed twice in cold PBS, fixed with 0.5 ml of 70% ethanol, and cultured for 1 h at 4 °C. Then, 50 µg/ml propidium iodide (Sigma‒Aldrich) was used to stain these cells. The cells were filtered by a 300-mesh filter, and then we recorded the cell cycle phase of each cell through the stained nuclei using a BD FACSCalibur flow cytometer (BD Biosciences, USA).

### Analysis of stress and apoptosis

To evaluate whether the lack of *DDX49* affects the apoptosis pathway, the PathScan® Stress and Apoptosis Signalling Antibody Array Kit was used. This kit tested the phosphorylation statuses of ERK1/2, Akt, Bad, HSP27, Smad2, p53, p38 MAPK, SAPK/JNK, Chk1, Chk2, IkBa, eIF2a, and TAK1, the cleaved levels of PARP, cleaved caspase-3, and cleaved caspase-7, and the total expression levels of IkBa, survivin, and α-tubulin. First, after 48 h of transfection with shDDX49 or shCtrl lentivirus, we collected and washed the PC-3 cells twice with cold PBS. The cell protein concentrations were regulated to 0.2–1.0 mg/ml. After treatment with blocking buffer for 15 min, all 50–75-µl cell protein samples were introduced into every tube and cultured at 4 °C overnight. On the second day, the cell protein solution was erased, and the plate was rinsed three times for 5 min each and then treated with 75 µl of 1 × antibody while gently shaking for 1 h at room temperature. Then, 75 µl of 1 × HRP-streptavidin was added for 30 min. Finally, the plate was washed three times and then exposed and visualized on the CLINX ChemiScope 5300 machine.

### Statistical analysis

To explore the association of *DDX49* expression with clinicopathological characteristics, Fisher’s exact test and Pearson’s chi-squared test were conducted, while Student’s t-test was adopted to assess the differences between groups. Recurrence-free survival (RFS) was analysed with the Kaplan–Meier method, and differences were assessed with the log-rank test. Multivariate recurrence-associated analysis was carried out with logistic regression analysis. The relative risks of PCa recurrence were denoted as adjusted hazard ratios and corresponding 95% confidence intervals (95% CIs). The statistical data are displayed as the mean ± SD. For all analyses, *p* < 0.05 indicated statistical significance. In addition, SPSS version 22.0 (SPSS Inc., Chicago, IL, USA) was involved in statistical analyses.

## Results

### DDX49 expression is enhanced in PCa tissues

**Fig. 1:**
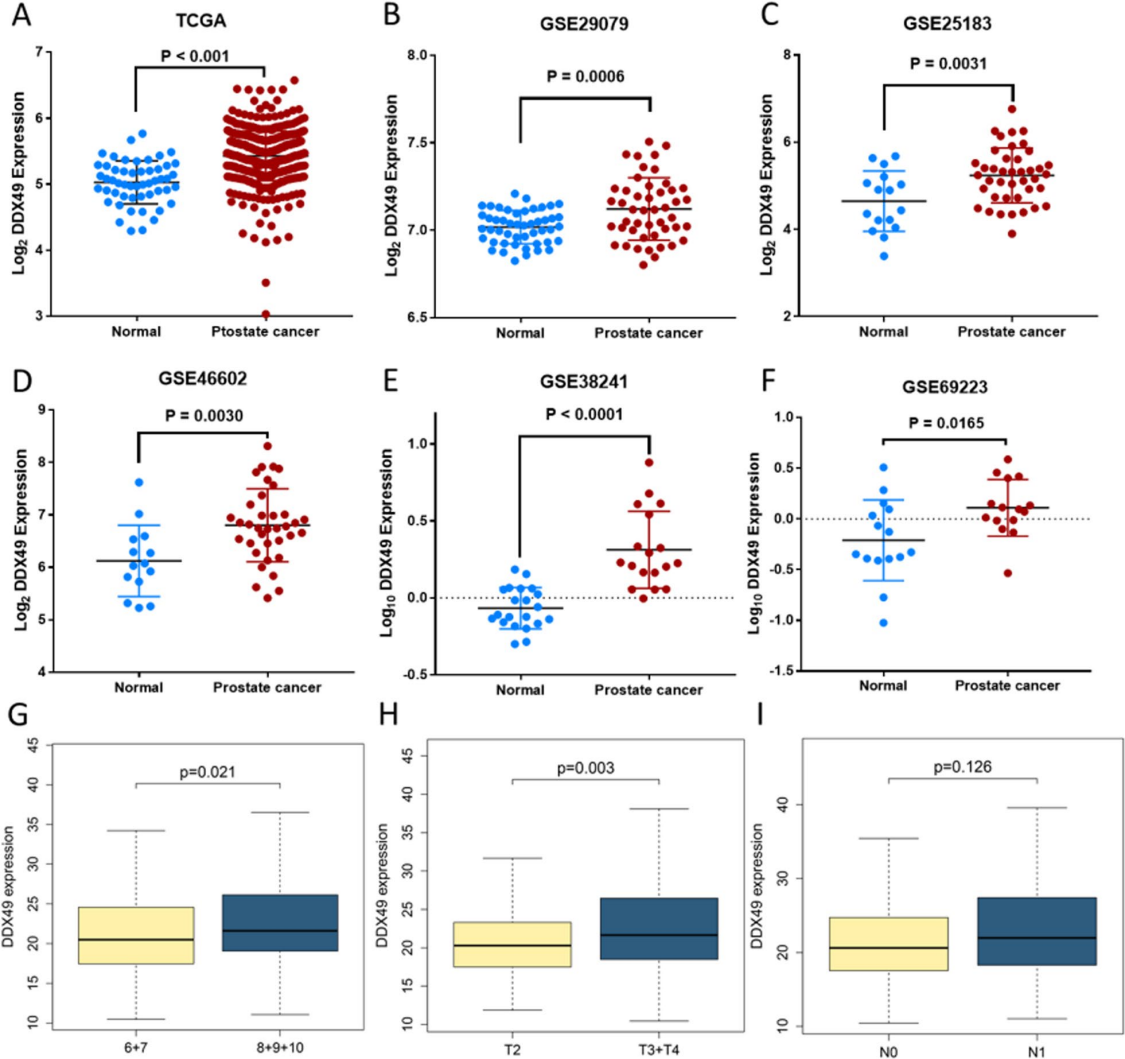
*DDX49* expression in prostate cancer and normal tissues. **A**–**F** The different expression of *DDX49* between tumor and normal tissues; The different expression of *DDX49* in early and advanced tumor stage represented by Gleason Score (**G**), T stage (**H**) and N stage (**I**)

The expression levels of *DDX49* between PCa tumour tissues and normal tissues were compared based on the TCGA-PRAD cohort, which contained the transcription profiles of 497 tumour tissues and 52 normal tissues. *DDX49* was significantly increased in PCa tumour tissues (*p* < 0.001) (Fig. 1A). For verification, we compared the differential expression of *DDX49* between tumour and normal tissues based on the RNA sequencing data from the GEO database, which included GSE29079 (*p* = 0.0006), GSE25183 (*p* = 0.0031), GSE46602 (*p* = 0.0030), GSE 38,241 (*p* < 0.0001), and GSE69223 (*p* = 0.0165), and found increased *DDX49* expression levels in PCa tissues compared with normal tissues (Fig. 1B–F). We found positive correlations for the *DDX49* expression level and clinicopathological stage of each PCa tissue. Patients with a Gleason score > 7 had higher *DDX49* expression than those with a Gleason score ≤ 7 (*p* = 0.021). Similar results were observed in patients with PCa categorized as T3/T4 (*p* = 0.003) or N1 (*p* = 0.126) (Fig. 1G–I).

### High DDX49 expression indicates a poor clinical outcome of PCa

**Fig. 2:**
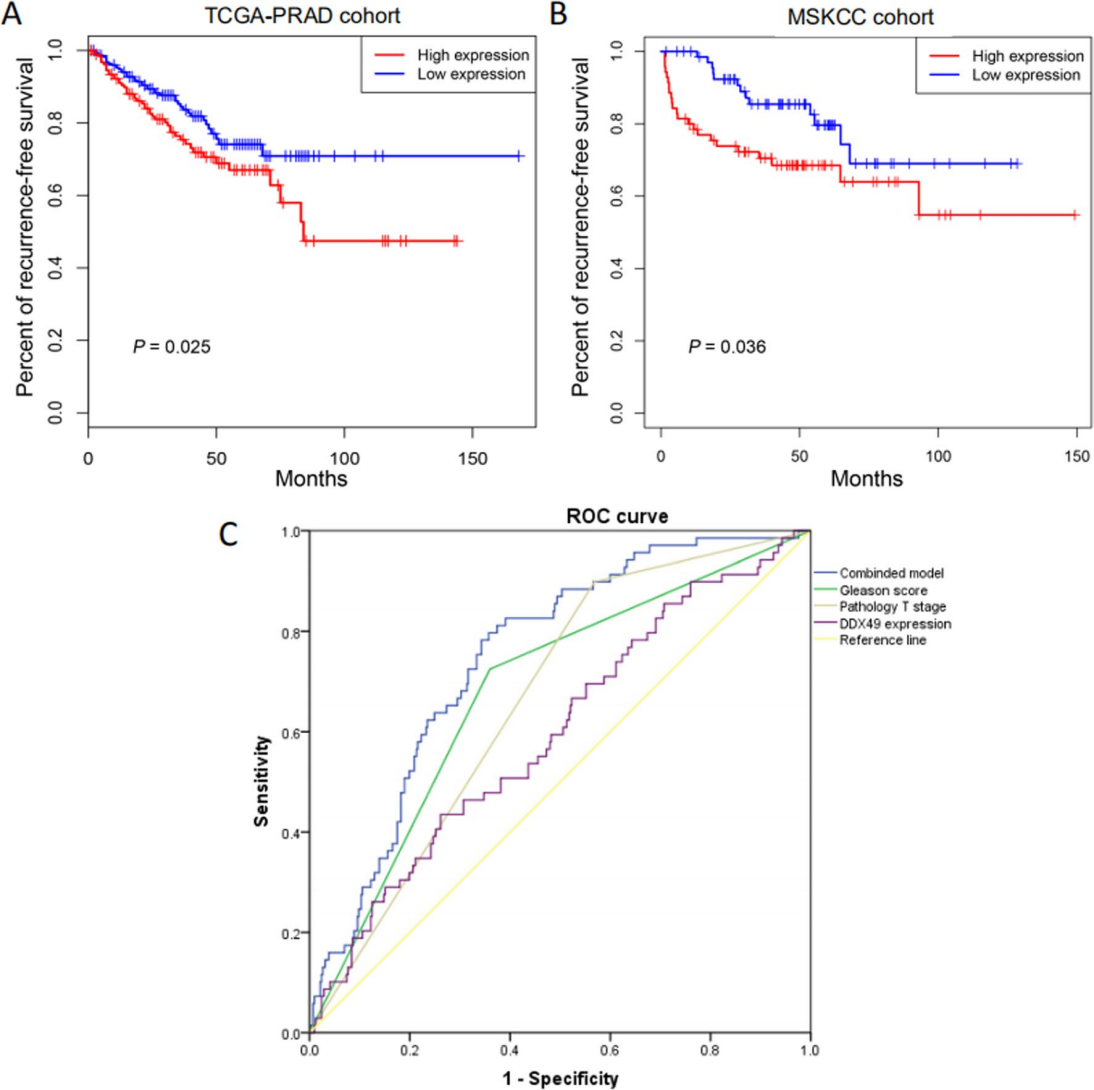
The association between *DDX49* and biochemical recurrence of PCa. **A** K–M curves for *DDX49* based on TCGA-PRAD cohort; **B** K–M curves for *DDX49* based on GEO cohort; **C** ROC curves for Gleason score, pathology T stage, DDX49 expression and the combined model in TCGA-PRAD cohort

Subsequently, we evaluated the association between RFS status, clinicopathological characteristics, and *DDX49* expression. As shown in Table 1, patients with a higher Gleason score showed a higher recurrence risk, as 72.46% of patients with Gleason score > 7 were in the recurrence group, in comparison with 36.09% in the recurrence-free group (*p* < 0.01). Consistently, patients in the recurrence group showed an increased risk of advanced PCa, judged by pathology T (*p* < 0.001), N (*p* = 0.002), and M (*p* < 0.001) stages. Meanwhile, elevated expression of *DDX49* was found in patients with recurrent PCa compared with patients who were free of recurrence (24.32 ± 7.14 vs. 22.02 ± 6.52, *p* = 0.008), indicating that *DDX49* may be involved in the biochemical recurrence of PCa. PCa tissues were further classified into high-expression and low-expression groups on the basis of the median expression level of *DDX49*, and K–M curves were used to compare the different RFS between groups. Based on the TCGA-PRAD cohorts, we found that RFS was shorter among patients with high *DDX49* expression than among patients with low *DDX49* expression (Fig. 2A, *p* = 0.025); this was also confirmed in the MSKCC cohorts (Fig. 2B, *p* = 0.036). Additionally, multivariate logistic regression analysis was conducted to enrol all the clinical features and *DDX49* expression, and the results indicated that Gleason score (HR = 2.504, 95% CI = 1.308–4.794, *p* = 0.006), pathology T stage (HR = 3.828, 95% CI = 1.519–9.648, *p* = 0.004), and *DDX49* (HR = 1.048, 95% CI = 1.005–1.093, *p* = 0.029) were independent risk factors related to PCa recurrence among patients (Table 2). ROC analysis was employed with the purpose of evaluating the discriminative efficiency of using T stage, Gleason score, or *DDX49* expression alone and in combination; the combination displayed a better discriminative efficiency of PCa recurrence likelihood than did each single factor (AUC = 0.750) (Fig. 2C).

**Table 1:** Clinical and demographic features of the patients

	Recurrence-free	Recurrence	*p* value
Age, Mean ± SD	60.91 ± 7.01	61.91 ± 5.62	0.187
Gleason score, N (%)			
6	45 (10.61%)	0	< 0.001*
7	226 (53.30%)	19 (27.54%)	
8	54 (12.74%)	9 (13.04%)	
9	95 (22.41%)	41 (59.42%)	
10	4 (0.94%)	0	
PSA, N (%)			
< = 4	350 (94.85%)	59 (88.06%)	0.065
> 4	19 (5.15%)	8 (11.94%)	
HIS, N (%)			
Acinar	414 (97.64%)	64 (92.75%)	0.07
Other	10 (2.36%)	5 (7.25%)	
Clinical M stage, N (%)			
M0	386 (99.23%)	65 (100%)	0.477
M1	3 (0.77%)	0	
Pathology T stage, N (%)			
T2	180 (43.17%)	7 (10.14%)	< 0.001*
T3	228 (54.68%)	61 (88.41%)	
T4	9 (2.16%)	1 (1.45%)	
Pathology N stage, N (%)			
N0	298 (83.94%)	45 (68.18%)	0.002*
N1	57 (16.06%)	21 (31.82%)	
Stage, N (%)			
I	32 (7.60%)	0	< 0.001*
II	142 (33.73%)	5 (7.25%)	
III	188 (44.66%)	43 (62.32%)	
IV	59 (14.01)	21 (30.43%)	
DDX49 expression, Mean ± SD	22.02 ± 6.52	24.32 ± 7.14	0.008*

Correlations between the recurrence status and clinical parameters were assessed by Student’s T-test, Mann–Whitney U test or Chi-Square test. *p < 0.05

**Table 2:** Multivariate logistic regression analysis of factors effective on PCa recurrence

Parameters	HR	95% CI	*p* value	AUC
Gleason				0.682
6 + 7	Reference	Reference	Reference	
8 + 9 + 10	2.504	1.308–4.794	0.006*	
Pathology T stage				0.665
T2	Reference	Reference	Reference	
T3 + T4	3.828	1.519–9.648	0.004*	
DDX49	1.048	1.005–1.093	0.029*	0.596
Combined model	–	–	–	0.750

*P < 0.05

### Protein interaction network of DDX49 and gene enrichment analyses

**Fig. 3:**
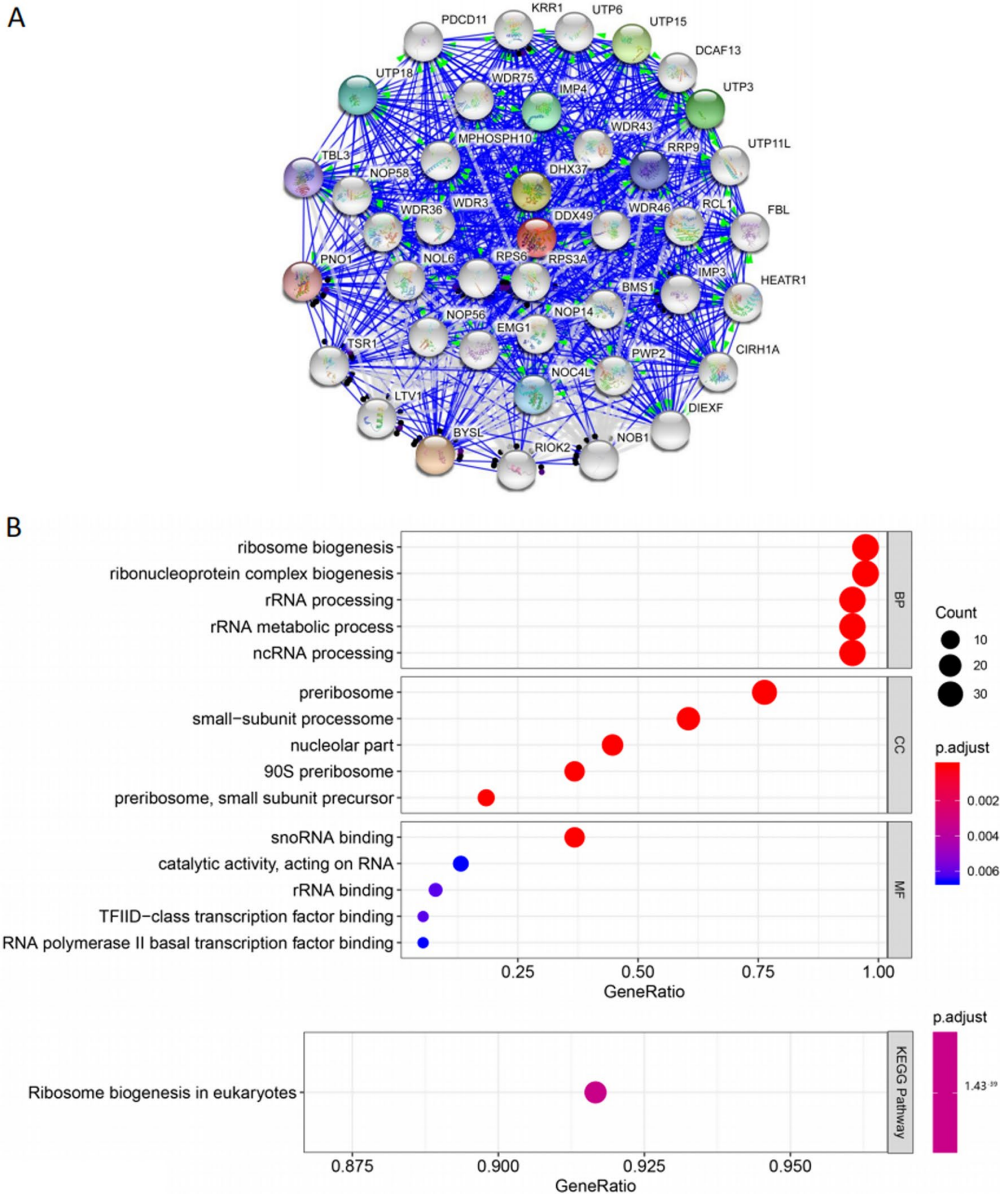
Functional enrichment analyses of the DEGs between the two groups and protein interaction network. **A** Protein interaction network of *DDX49*; **B** GO and KEGG pathway analysis

To investigate the functionally related or interaction partners of *DDX49*, 40 DEGs were eventually identified and used to establish a protein interaction network of *DDX49* via the STRING database. We set a high confidence of 0.7, and the active interactions came from experiments, text mining, databases, gene fusion, coexpression, neighbourhood, or cooccurrence (Additional file 1: Table S1, Fig. 3A). Furthermore, GO and KEGG enrichment analyses were adopted to study the signalling pathway activation patterns among the *DDX49* high-expression and low-expression groups. As shown in Fig. 3B, the DEGs were tightly linked to ribosome biogenesis, rRNA processing, rRNA metabolism, and ncRNA processing in the biological process GO terms. Within cellular components, DEGs were linked to per-ribosome, small-subunit processome, nucleolar part, and 90S preribosome. For molecular function, snoRNA binding and rRNA binding GO terms were identified. Consistently, the results of KEGG enrichment also showed an association with ribosome biogenesis (Additional file 2: Table S2), suggesting a role for ribosome biogenesis in PCa.

### DDX49 expression was enhanced in PCa cell lines, but DDX49 knockdown inhibited proliferation

**Fig. 4:**
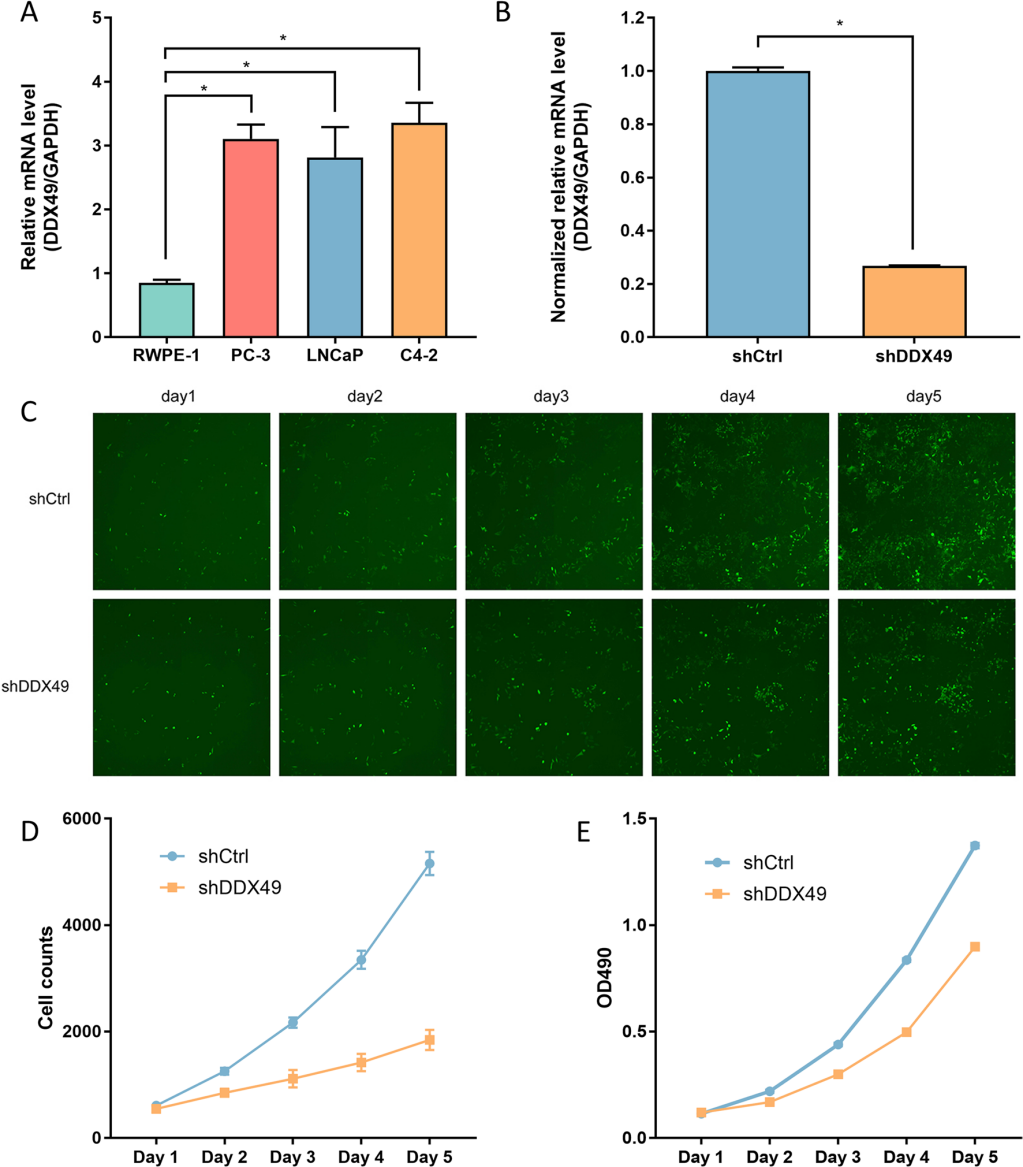
Increased *DDX49* in PCa cell lines and knockdown of *DDX49* in PC-3 cells inhibits cell proliferation. **A** Comparison of *DDX49* expression among PCa cells lines and normal prostate epithelium cell; **B** knockdown of *DDX49* via *DDX49*-shRNA lentivirus in PC-3 cells; **C** Detection of cell proliferation by Celigo method after treating with *DDX49*-shRNA or NC lentivirus in 1–5 days; **D** The quantification of cell numbers each group in 1–5 days; **E** the fold change of PC-3 treated with *DDX49*-shRNA or NC lentivirus in 1–5 days in MTT assay

qPCR was employed to test the *DDX49* mRNA expression levels among prostate epithelium cells, RWPE-1, and three other PCa cell lines, including PC-3, LNCaP, and C4-2. Figure 4A shows the high expression of *DDX49* in all PCa cell lines relative to the normal prostate epithelium. Among them, the expression level of *DDX49* was highest in C4-2, followed by PC3 and LNCaP, indicating that although DDX49 is highly expressed in PCa, the expression level varies among different types of PCa cell lines.

Next, we packaged the shDDX49 lentivirus to test the potential biological function of *DDX49*. We initially infected PC-3 cells with shDDX49 lentivirus and found that the shDDX49 lentivirus knocked down *DDX49* expression by 73.2% compared to the shCtrl lentivirus (Fig. 4B). As shown in Fig. 4C and quantitatively confirmed in Fig. 4D, the positive cell numbers of the shCtrl group expanded dramatically over five days compared to those of the shDDX49 group PC-3 cells. This illustrated that knockdown of *DDX49* leads to the inhibition of PC-3 cell growth. We also used an MTT assay to evaluate changes in cell proliferation between the shDDX49 and shCtrl groups, and the obtained results were similar. On the fifth day, the fold change in the PC-3 cells in the shCtrl group was 11.95, while the fold change in the shDDX49 group was only 7.49 (Fig. 4E).

### Knockdown of DDX49 leads to cell cycle arrest

**Fig. 5:**
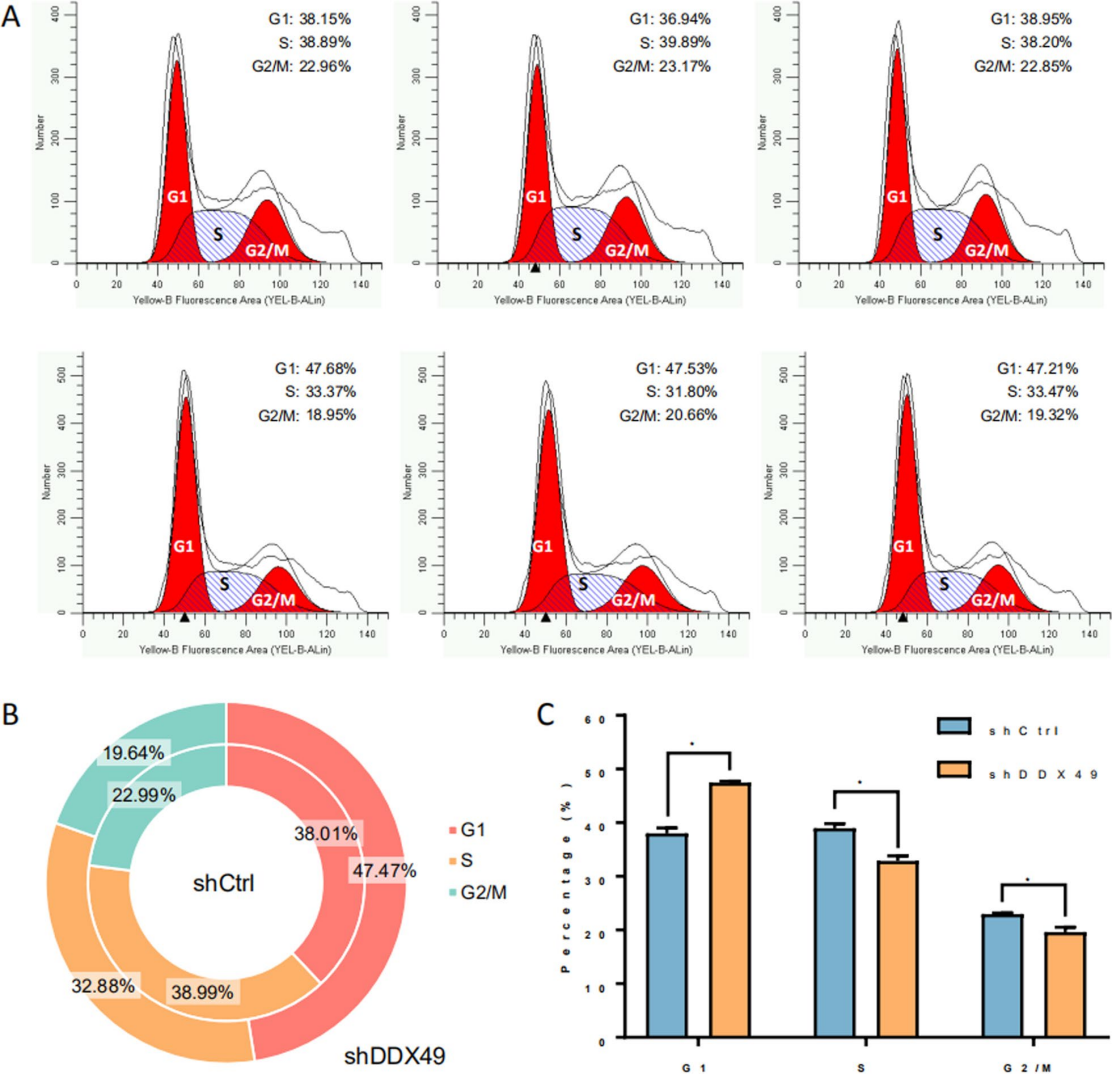
Knockdown of *DDX49* in PC-3 cells leads to cell cycle arrest. **A** The representative result of cell cycle in shCtrl and shDDX49 PC-3 group; **B**–**C** The quantification of cell cycle distribution in shCtrl and shDDX49 PC-3 group

This study further evaluated the impacts of *DDX49* knockdown on the cell cycle of PC-3 cells. In the shCtrl group, 38.01 ± 1.01% of cells were in G1 phase, 38.99 ± 0.85% were in S phase, and 22.99 ± 0.16% were in G2/M phase. Meanwhile, the shDDX49 group displayed a different distribution of cell cycle phases: 47.47 ± 0.24% in G1 phase and 32.88 ± 0.94% in S phase, and the proportion of G2/M phase cells was reduced to 19.64 ± 0.90% (Fig. 5A–B). As shown in Fig. 5C, shDDX49 lentivirus infection significantly increased the proportion of G1 phase PC-3 cells relative to the shCtrl group (*p* < 0.01), whereas the distributions of G2/M and S phase cells were dramatically decreased (*p* < 0.01).

### Knockdown of DDX49 affects stress and apoptosis pathway genes

**Fig. 6:**
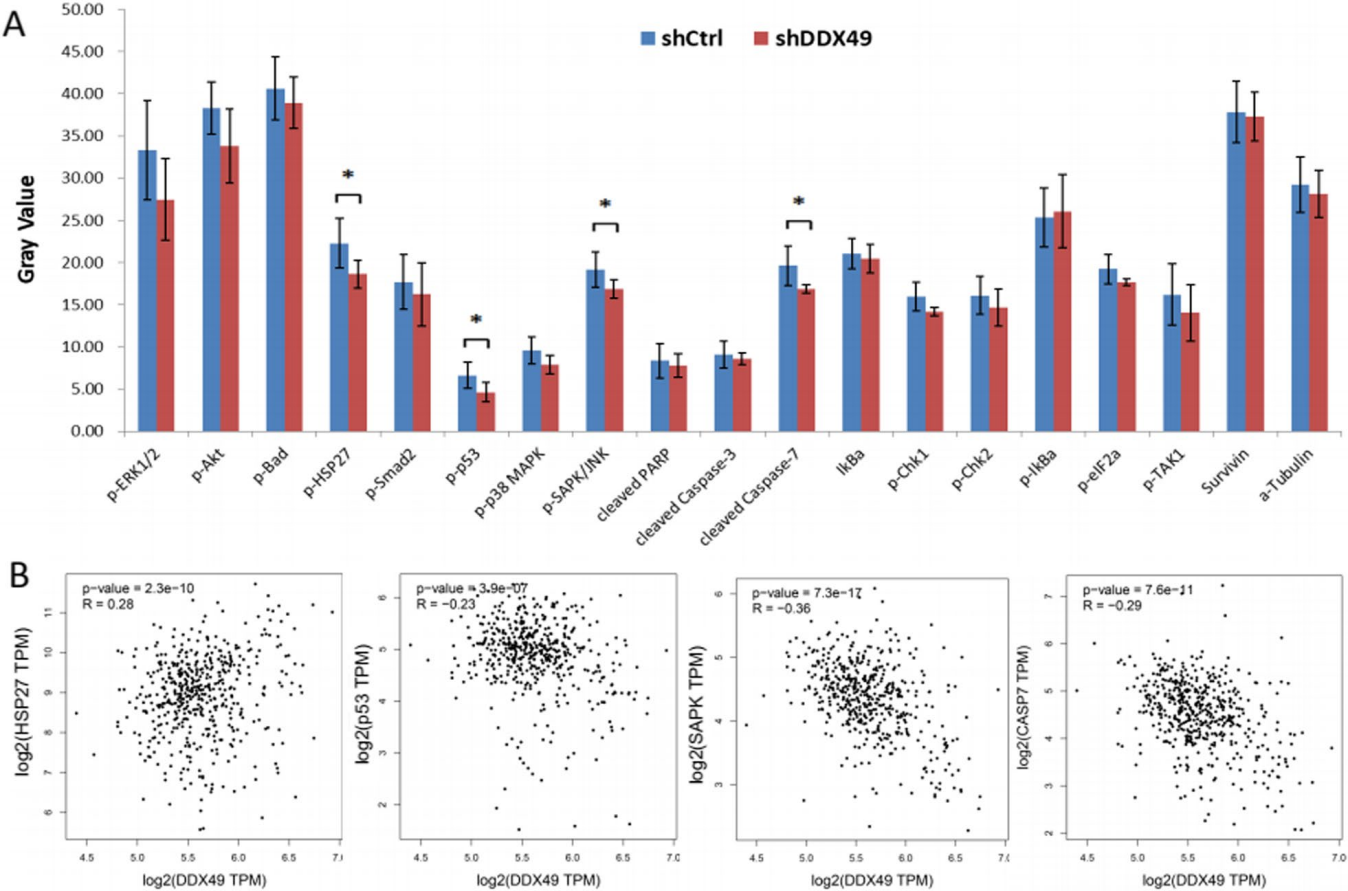
Knockdown of *DDX49* affects stress and apoptosis pathway gene. **A** The Phosphorylation of p-HSP27, p-p53 and p-SAPK/JNK decreased in shDDX49 PC-3 group than shCtrl PC-3 group, and the cleaved Caspase-7 level was also decreased. **B** The correlations between the expression of *DDX49* and HSP27, p-53, SAPK and CASP7

By adopting the PathScan® Stress and Apoptosis Signalling Antibody Array Kit, we detected changes in stress and apoptosis signalling markers caused by suppression of *DDX49*. We found that the phosphorylation levels of HSP27, p53, and SAPK/JNK were decreased in the shDDX49 group compared with the shCtrl group. Likewise, cleaved caspase-7 was also decreased in the shDDX49 group (Fig. 6A). Additionally, the association between the mRNA levels of stress and apoptosis pathway genes was studied based on the TCGA-PRAD cohort. The expression level of *DDX49* mRNA showed a significant relationship with *HSP27* (R = 0.28, *p* < 0.001), *p53* (R = − 0.23, *p* < 0.001), *SAPK* (R = − 0.36, *p* < 0.001), and *CASP7* (R = − 0.29, *p* < 0.001) (Fig. 6B).

## Discussion

In recent decades, PCa has been considered the dominant cause of cancer-specific death among males [23]. Although patients with primary PCa have a 5-year survival of more than 80%, advanced PCa always recurs within 2–3 years after the first treatment, after becoming castration-resistant [24, 25]. Insight into the molecular mechanisms of PCa reveals prognostic and therapeutic heterogeneity. The comprehensive molecular alteration landscape of PCa has been elucidated, and many pivotal mutant genes have been identified, such as *ERG, ETV1/4, FLI1, SPOP, FOXA1* and *IDH1*; however, prior studies are still insufficient to cover all patients with PCa, and nearly 26% of available PCa tissue samples require further study to investigate their gene signatures [26]. Through bioinformatics and robust statistical methods, we identified *DDX49* as a potential prognostic gene for PCa.

The *DDX* gene belongs to the helicase 2 superfamily and is involved in almost every aspect of RNA metabolism [27, 28]. They engage in various cellular functions, from splicing, mRNA nuclear output, and mRNA degradation to translation [29]. A growing number of studies have considered it to be a promising translational target of cancer therapeutics [30, 31]. Therefore, DDX genes are an attractive target for cancer treatment and underlying biomarkers in the diagnosis and prognosis of many cancers, including PCa.

*DDX49*, a member of the DEAD-box RNA helicase protein family, has been shown to maintain stable levels of preribosomal 47S RNA and regulate global translation [32]. This property of *DDX49* may be hijacked in cancer to promote uncontrolled cell proliferation, consistent with numerous studies. Lian et al. [33] discovered the remarkable overexpression of *DDX49* in lung adenocarcinoma and cell lines in comparison with paracancerous tissues and normal cells, and *DDX49* knockdown suppressed the viability and invasion of lung cancer cells. *DDX49* expression among breast cancer patients was also markedly increased. In addition, *DDX49* may promote the growth of breast cancer stem cells by regulating the expression of Oct3/4, SOX-2 and other proteins, thus promoting the development of the disease [34]. In addition, Dai et al. also reported that *DDX49* is overexpressed in hepatocellular carcinoma and leads to poor prognosis [35]. However, the relationship between *DDX49* and the prognosis of PCa has not been explored until now.

This research first confirmed the relationship between high expression of *DDX49* and the recurrence of PCa and that DDX49 is predictive of a poor patient prognosis. Additionally, the proportion of PC-3 cells arrested in G1 phase was significantly enhanced after *DDX49* knockdown. As cell cycle arrest reflects the alteration of apoptosis [36], we next evaluated the potential influence of reduced *DDX49* expression on the protein pathways regulating apoptosis. *DDX49* knockdown decreased the phosphorylation abundance of p53, an important protein that is involved in regulating apoptosis in PCa [37]. We speculate that p53 possibly plays a vital role in the proliferation-promoting effect of *DDX49* on PCa cells. Furthermore, the phosphorylated form of HSP-27 was decreased after *DDX49* knockdown. Interestingly, previous reports have confirmed that Hsp-27 can promote the occurrence of CRPC by protecting eukaryotic translation initiation factor 4E from degradation [38]. This result indicates that *DDX49* may not only play a cancer-promoting role in prostate cells by affecting p-HSP27 but also exert significant effects on CRPC development via p-HSP27. In this study, we also showed a decreased level of SAPK/JNK phosphorylation, indicating that SAPK/JNK may participate in the cancer-promoting function of *DDX49* in PCa. However, the exact mechanism by which *DDX49* affects the phosphorylation abundance of these proteins is unclear. Recent studies have shown that *DDX3* can bind to casein kinase 1 as a regulatory subunit to directly stimulate its kinase activity and promote protein phosphorylation [39]. We speculate that *DDX49* may promote the phosphorylation of these proteins in a similar way. Further work is needed to elucidate how *DDX49* affects these possible pathways to modulate the cell cycle, apoptosis, and cell proliferation.

## Conclusions

In summary, this study demonstrates the association of high *DDX49* expression with PCa recurrence, which is predictive of a poor patient prognosis. In vitro results also demonstrated that the downregulation of *DDX49* expression suppressed cell proliferation, blocked the cell cycle, and facilitated cell apoptosis. Therefore, knockdown of *DDX49* may become a potential new strategy to treat PCa patients.

## Supplementary Information

**Additional file 1: Table S1.** The selection of DDX49 associated gene from the STRING.

**Additional file 2: Table S2.** The GO and KEGG enrichment results of DDX49 related genes.

## Data Availability

The mRNA-Seq data and clinical follow-up data associated with the prostate cancer tissues samples (TCGA-PRAD) were downloaded from the TCGA (https://portal.gdc.cancer.gov/repository). The datasets of gene expression profiles for prostate cancer (GSE29079, GSE25183, GSE46602, GSE38241, and GSE69223) are available in the GEO (https://www.ncbi.nlm.nih.gov/geo/).

## Abbreviations

DDX: DEAD-box helicases
PCa: Prostate cancer
ADT: Androgen deprivation therapy
CRPC: Castration-resistant prostate cancer
AR: Androgen receptor
TCGA: The Cancer Genome Atlas
GEO: The Gene Expression Omnibus
shRNA: Short hairpin RNA
K-SFM: Keratinocyte serum free medium
EGF: Epidermal growth factor
FBS: Foetal bovine serum
qRT‒PCR: Quantitative reverse transcription–polymerase chain reaction
RFS: Recurrence free survival
