# Supplementary material for: Overexpression of DDX49 in prostate cancer is associated with poor prognosis
Source: BMC Urol. 2023 Apr 27;23:66. doi: 10.1186/s12894-023-01251-4 (PMC10134639; doi:10.1186/s12894-023-01251-4)
Supplement: Supplementary file 1 — Additional file 1: Table S1. The selection of DDX49 associated gene from the STRING. [file 12894_2023_1251_MOESM1_ESM.pdf]

Table S1. The selection of DDX49 associated gene from the STRING.

Gene 1	Gene 2	node1_string_internal_id	node2_string_internal_id	node1_external_id	node2_external_id	neighborhood_on_chromosome	gene_fusion	phylogenetic_cooccurrence	homology	coexpression	experimentally_determined_interaction	database_annotated	automated_textmining	combined_score
WDR46	WDR75	4444423	4438919	9606.ENSP00000363746	9606.ENSP00000314193	0	0	0	0	0.872	0.797	0.9	0.689	0.999
CIRH1A	WDR75	4439903	4438919	9606.ENSP00000327179	9606.ENSP00000314193	0	0	0	0	0.882	0.924	0.9	0.892	0.999
TSR1	NOB1	4437751	4435851	9606.ENSP00000301364	9606.ENSP00000268802	0	0	0	0	0.861	0.969	0.9	0.891	0.999
DCAF13	UTP18	4437420	4433308	9606.ENSP00000297579	9606.ENSP00000225298	0	0	0	0	0.919	0.784	0.9	0.72	0.999
TBL3	UTP18	4450478	4433308	9606.ENSP00000454836	9606.ENSP00000225298	0	0	0	0	0.888	0.798	0.9	0.896	0.999
WDR3	NOL6	4438389	4437451	9606.ENSP00000308179	9606.ENSP00000297990	0	0	0	0	0.894	0.758	0.9	0.683	0.999
NOP14	HEATR1	4448347	4442698	9606.ENSP00000405068	9606.ENSP00000355541	0	0	0	0	0.897	0.784	0.9	0.66	0.999
NOP58	FBL	4435411	4433181	9606.ENSP00000264279	9606.ENSP00000221801	0	0	0	0	0.927	0.962	0.9	0.966	0.999
IMP4	UTP3	4434687	4434386	9606.ENSP00000259239	9606.ENSP00000254803	0	0	0	0	0.895	0.962	0.9	0.686	0.999
TBL3	WDR46	4450478	4444423	9606.ENSP00000454836	9606.ENSP00000363746	0	0	0	0	0.897	0.797	0.9	0.726	0.999
WDR36	DCAF13	4449235	4437420	9606.ENSP00000423067	9606.ENSP00000297579	0	0	0	0	0.877	0.97	0.9	0.456	0.999
RCL1	IMP4	4445592	4434687	9606.ENSP00000371169	9606.ENSP00000259239	0	0	0	0	0.924	0.784	0.9	0.563	0.999
WDR3	BYSL	4438389	4433499	9606.ENSP00000308179	9606.ENSP00000230340	0	0	0	0	0.89	0.983	0.9	0.321	0.999
PNO1	BYSL	4435289	4433499	9606.ENSP00000263657	9606.ENSP00000230340	0	0	0	0	0.929	0.992	0.9	0.914	0.999
NOL6	UTP18	4437451	4433308	9606.ENSP00000297990	9606.ENSP00000225298	0	0	0	0	0.865	0.788	0.9	0.82	0.999
TBL3	WDR36	4450478	4449235	9606.ENSP00000454836	9606.ENSP00000423067	0	0	0	0.545	0.905	0.967	0.9	0.881	0.999
WDR46	DCAF13	4444423	4437420	9606.ENSP00000363746	9606.ENSP00000297579	0	0	0	0	0.902	0.792	0.9	0.766	0.999
WDR43	UTP18	4447102	4433308	9606.ENSP00000384302	9606.ENSP00000225298	0	0	0	0	0.904	0.666	0.9	0.807	0.999
IMP3	RRP9	4439890	4433566	9606.ENSP00000326981	9606.ENSP00000232888	0	0	0	0	0.828	0.957	0.9	0.763	0.999
HEATR1	UTP15	4442698	4437338	9606.ENSP00000355541	9606.ENSP00000296792	0	0	0	0	0.889	0.872	0.9	0.917	0.999
IMP3	MPHOSPH	4439890	4433887	9606.ENSP00000326981	9606.ENSP00000244230	0	0	0	0	0.661	0.971	0.9	0.655	0.999
WDR46	UTP15	4444423	4437338	9606.ENSP00000363746	9606.ENSP00000296792	0	0	0	0	0.868	0.797	0.9	0.778	0.999
RIOK2	BYSL	4436521	4433499	9606.ENSP00000283109	9606.ENSP00000230340	0	0	0	0	0.836	0.944	0.9	0.949	0.999
WDR43	MPHOSPH	4447102	4433887	9606.ENSP00000384302	9606.ENSP00000244230	0	0	0	0	0.876	0.788	0.9	0.741	0.999
IMP3	UTP3	4439890	4434386	9606.ENSP00000326981	9606.ENSP00000254803	0	0	0	0	0.655	0.965	0.9	0.536	0.999
NOP14	WDR46	4448347	4444423	9606.ENSP00000405068	9606.ENSP00000363746	0	0	0	0	0.918	0.797	0.9	0.586	0.999
WDR43	CIRH1A	4447102	4439903	9606.ENSP00000384302	9606.ENSP00000327179	0	0	0	0	0.922	0.932	0.9	0.952	0.999
TBL3	WDR3	4450478	4438389	9606.ENSP00000454836	9606.ENSP00000308179	0	0	0.3	0.575	0.905	0.969	0.9	0.881	0.999
PDCD11	BYSL	4443395	4433499	9606.ENSP00000358812	9606.ENSP00000230340	0	0	0	0	0.853	0.736	0.9	0.823	0.999
WDR36	RRP9	4449235	4433566	9606.ENSP00000423067	9606.ENSP00000232888	0	0	0	0	0.896	0.957	0.9	0.646	0.999
WDR36	NOP56	4449235	4445513	9606.ENSP00000423067	9606.ENSP00000370589	0	0	0	0	0.891	0.96	0.9	0.573	0.999
LTV1	PNO1	4442915	4435289	9606.ENSP00000356548	9606.ENSP00000263657	0	0	0	0	0.845	0.944	0.9	0.916	0.999
PWP2	UTP6	4436922	4434930	9606.ENSP00000291576	9606.ENSP00000261708	0	0	0	0	0.819	0.742	0.9	0.859	0.999
UTP6	MPHOSPH	4434930	4433887	9606.ENSP00000261708	9606.ENSP00000244230	0	0	0	0	0.862	0.798	0.9	0.743	0.999
TBL3	WDR43	4450478	4447102	9606.ENSP00000454836	9606.ENSP00000384302	0	0	0	0	0.899	0.718	0.9	0.759	0.999
WDR36	WDR75	4449235	4438919	9606.ENSP00000423067	9606.ENSP00000314193	0	0	0	0	0.892	0.784	0.9	0.722	0.999
WDR43	UTP15	4447102	4437338	9606.ENSP00000384302	9606.ENSP00000296792	0	0	0	0	0.923	0.862	0.9	0.957	0.999
TSR1	PNO1	4437751	4435289	9606.ENSP00000301364	9606.ENSP00000263657	0	0	0	0	0.887	0.958	0.9	0.894	0.999
IMP4	RRP9	4434687	4433566	9606.ENSP00000259239	9606.ENSP00000232888	0	0	0	0	0.894	0.796	0.9	0.682	0.999
TBL3	UTP15	4450478	4437338	9606.ENSP00000454836	9606.ENSP00000296792	0	0	0.362	0.564	0.903	0.968	0.9	0.854	0.999
NOL6	NOP58	4437451	4435411	9606.ENSP00000297990	9606.ENSP00000264279	0	0	0	0	0.862	0.958	0.9	0.678	0.999
NOP58	MPHOSPH	4435411	4433887	9606.ENSP00000264279	9606.ENSP00000244230	0	0	0	0	0.874	0.96	0.9	0.768	0.999

Gene 1	Gene 2	node1_string_internal_id	node2_string_internal_id	node1_external_id	node2_external_id	neighborhood_on_chromosome	gene_fusion	phylogenetic_cooccurrence	homology	coexpression	experimentally_determined_interaction	database_annotated	automated_textmining	combined_score
PWP2	MPHOSPH	4436922	4433887	9606.ENSP00000291576	9606.ENSP00000244230	0	0	0	0	0.731	0.963	0.9	0.812	0.999
TBL3	HEATR1	4450478	4442698	9606.ENSP00000454836	9606.ENSP00000355541	0	0	0	0	0.901	0.796	0.9	0.82	0.999
DCAF13	PNO1	4437420	4435289	9606.ENSP00000297579	9606.ENSP00000263657	0	0	0	0	0.893	0.784	0.9	0.622	0.999
DCAF13	RRP9	4437420	4433566	9606.ENSP00000297579	9606.ENSP00000232888	0	0	0	0	0.902	0.97	0.9	0.736	0.999
PWP2	RRP9	4436922	4433566	9606.ENSP00000291576	9606.ENSP00000232888	0	0	0	0.567	0.833	0.966	0.9	0.759	0.999
CIRH1A	UTP18	4439903	4433308	9606.ENSP00000327179	9606.ENSP00000225298	0	0	0	0	0.895	0.798	0.9	0.846	0.999
BMS1	BYSL	4444407	4433499	9606.ENSP00000363642	9606.ENSP00000230340	0	0	0	0	0.886	0.73	0.9	0.778	0.999
NOP14	RRP9	4448347	4433566	9606.ENSP00000405068	9606.ENSP00000232888	0	0	0	0	0.879	0.796	0.9	0.755	0.999
IMP3	IMP4	4439890	4434687	9606.ENSP00000326981	9606.ENSP00000259239	0	0	0	0	0.902	0.796	0.9	0.842	0.999
HEATR1	NOP58	4442698	4435411	9606.ENSP00000355541	9606.ENSP00000264279	0	0	0	0	0.902	0.798	0.9	0.636	0.999
CIRH1A	PWP2	4439903	4436922	9606.ENSP00000327179	9606.ENSP00000291576	0	0	0	0	0.817	0.742	0.9	0.826	0.999
UTP15	NOP58	4437338	4435411	9606.ENSP00000296792	9606.ENSP00000264279	0	0	0	0	0.882	0.933	0.9	0.588	0.999
BMS1	UTP18	4444407	4433308	9606.ENSP00000363642	9606.ENSP00000225298	0	0	0	0	0.863	0.79	0.9	0.752	0.999
LTV1	RIOK2	4442915	4436521	9606.ENSP00000356548	9606.ENSP00000283109	0	0	0	0	0.746	0.953	0.9	0.919	0.999
RCL1	PNO1	4445592	4435289	9606.ENSP00000371169	9606.ENSP00000263657	0	0	0	0	0.851	0.784	0.9	0.734	0.999
CIRH1A	DCAF13	4439903	4437420	9606.ENSP00000327179	9606.ENSP00000297579	0	0	0	0	0.866	0.798	0.9	0.759	0.999
BMS1	PDCD11	4444407	4443395	9606.ENSP00000363642	9606.ENSP00000358812	0	0	0	0	0.906	0.731	0.9	0.816	0.999
WDR43	NOP58	4447102	4435411	9606.ENSP00000384302	9606.ENSP00000264279	0	0	0	0	0.924	0.788	0.9	0.5	0.999
WDR46	BMS1	4444423	4444407	9606.ENSP00000363746	9606.ENSP00000363642	0	0	0	0	0.909	0.797	0.9	0.556	0.999
NOC4L	NOP58	4440054	4435411	9606.ENSP00000328854	9606.ENSP00000264279	0	0	0	0	0.87	0.959	0.9	0.116	0.999
NOL6	UTP6	4437451	4434930	9606.ENSP00000297990	9606.ENSP00000261708	0	0	0	0	0.883	0.788	0.9	0.792	0.999
RCL1	BMS1	4445592	4444407	9606.ENSP00000371169	9606.ENSP00000363642	0	0	0	0	0.443	0.798	0.9	0.976	0.999
UTP15	UTP18	4437338	4433308	9606.ENSP00000296792	9606.ENSP00000225298	0	0	0	0	0.896	0.798	0.9	0.842	0.999
DCAF13	KRR1	4437420	4433449	9606.ENSP00000297579	9606.ENSP00000229214	0	0	0	0	0.846	0.797	0.9	0.72	0.999
NOP14	WDR3	4448347	4438389	9606.ENSP00000405068	9606.ENSP00000308179	0	0	0	0	0.904	0.784	0.9	0.668	0.999
UTP6	UTP18	4434930	4433308	9606.ENSP00000261708	9606.ENSP00000225298	0	0	0	0	0.91	0.798	0.9	0.947	0.999
BYSL	KRR1	4433499	4433449	9606.ENSP00000230340	9606.ENSP00000229214	0	0	0	0	0.841	0.805	0.9	0.747	0.999
CIRH1A	WDR3	4439903	4438389	9606.ENSP00000327179	9606.ENSP00000308179	0	0	0	0	0.907	0.798	0.9	0.778	0.999
NOP14	NOL6	4448347	4437451	9606.ENSP00000405068	9606.ENSP00000297990	0	0	0	0	0.894	0.788	0.9	0.68	0.999
WDR75	DCAF13	4438919	4437420	9606.ENSP00000314193	9606.ENSP00000297579	0	0	0	0	0.882	0.784	0.9	0.722	0.999
NOP14	NOC4L	4448347	4440054	9606.ENSP00000405068	9606.ENSP00000328854	0	0	0	0	0.858	0.788	0.9	0.806	0.999
NOP56	MPHOSPH	4445513	4433887	9606.ENSP00000370589	9606.ENSP00000244230	0	0	0	0	0.841	0.962	0.9	0.714	0.999
WDR43	HEATR1	4447102	4442698	9606.ENSP00000384302	9606.ENSP00000355541	0	0	0	0	0.911	0.788	0.9	0.917	0.999
BMS1	RRP9	4444407	4433566	9606.ENSP00000363642	9606.ENSP00000232888	0	0	0	0	0.885	0.797	0.9	0.705	0.999
WDR3	PWP2	4438389	4436922	9606.ENSP00000308179	9606.ENSP00000291576	0	0	0	0.544	0.829	0.976	0.9	0.818	0.999
UTP15	UTP6	4437338	4434930	9606.ENSP00000296792	9606.ENSP00000261708	0	0	0	0	0.896	0.796	0.9	0.829	0.999
WDR36	BMS1	4449235	4444407	9606.ENSP00000423067	9606.ENSP00000363642	0	0	0	0	0.899	0.797	0.9	0.585	0.999
RCL1	BYSL	4445592	4433499	9606.ENSP00000371169	9606.ENSP00000230340	0	0	0	0	0.863	0.737	0.9	0.772	0.999
WDR36	WDR46	4449235	4444423	9606.ENSP00000423067	9606.ENSP00000363746	0	0	0	0	0.891	0.798	0.9	0.613	0.999
WDR43	NOL6	4447102	4437451	9606.ENSP00000384302	9606.ENSP00000297990	0	0	0	0	0.89	0.788	0.9	0.748	0.999
MPHOSPH	UTP18	4433887	4433308	9606.ENSP00000244230	9606.ENSP00000225298	0	0	0	0	0.85	0.96	0.9	0.798	0.999
TBL3	BMS1	4450478	4444407	9606.ENSP00000454836	9606.ENSP00000363642	0	0	0	0	0.893	0.797	0.9	0.691	0.999
NOP14	UTP15	4448347	4437338	9606.ENSP00000405068	9606.ENSP00000296792	0	0	0	0	0.897	0.787	0.9	0.721	0.999
HEATR1	RRP9	4442698	4433566	9606.ENSP00000355541	9606.ENSP00000232888	0	0	0	0	0.914	0.797	0.9	0.766	0.999

Gene 1	Gene 2	node1_string_internal_id	node2_string_internal_id	node1_external_id	node2_external_id	neighborhood_on_chromosome	gene_fusion	phylogenetic_cooccurrence	homology	coexpression	experimentally_determined_interaction	database_annotated	automated_textmining	combined_score
NOP56	DCAF13	4445513	4437420	9606.ENSP00000370589	9606.ENSP00000297579	0	0	0	0	0.845	0.956	0.9	0.736	0.999
RPS6	RPS3A	4445386	4441583	9606.ENSP00000369757	9606.ENSP00000346050	0	0	0	0	0.997	0.992	0.9	0.721	0.999
TBL3	NOP56	4450478	4445513	9606.ENSP00000454836	9606.ENSP00000370589	0	0	0	0	0.896	0.956	0.9	0.691	0.999
TBL3	MPHOSPH	4450478	4433887	9606.ENSP00000454836	9606.ENSP00000244230	0	0	0	0	0.854	0.97	0.9	0.815	0.999
TBL3	NOP58	4450478	4435411	9606.ENSP00000454836	9606.ENSP00000264279	0	0	0	0	0.894	0.971	0.9	0.759	0.999
EMG1	UTP18	4450965	4433308	9606.ENSP00000470560	9606.ENSP00000225298	0	0	0	0	0.877	0.784	0.9	0.694	0.999
TBL3	UTP6	4450478	4434930	9606.ENSP00000454836	9606.ENSP00000261708	0	0	0	0	0.889	0.798	0.9	0.894	0.999
WDR46	BYSL	4444423	4433499	9606.ENSP00000363746	9606.ENSP00000230340	0	0	0	0	0.897	0.758	0.9	0.644	0.999
NOP14	CIRH1A	4448347	4439903	9606.ENSP00000405068	9606.ENSP00000327179	0	0	0	0	0.893	0.798	0.9	0.717	0.999
WDR46	KRR1	4444423	4433449	9606.ENSP00000363746	9606.ENSP00000229214	0	0	0	0	0.852	0.797	0.9	0.736	0.999
WDR46	UTP6	4444423	4434930	9606.ENSP00000363746	9606.ENSP00000261708	0	0	0	0	0.902	0.797	0.9	0.661	0.999
UTP3	MPHOSPH	4434386	4433887	9606.ENSP00000254803	9606.ENSP00000244230	0	0	0	0	0.886	0.968	0.9	0.806	0.999
PDCD11	HEATR1	4443395	4442698	9606.ENSP00000358812	9606.ENSP00000355541	0	0	0	0	0.908	0.737	0.9	0.787	0.999
EMG1	NOP14	4450965	4448347	9606.ENSP00000470560	9606.ENSP00000405068	0	0	0	0	0.465	0.798	0.9	0.989	0.999
TBL3	FBL	4450478	4433181	9606.ENSP00000454836	9606.ENSP00000221801	0	0	0	0	0.812	0.96	0.9	0.62	0.999
DCAF13	UTP15	4437420	4437338	9606.ENSP00000297579	9606.ENSP00000296792	0	0	0	0	0.905	0.784	0.9	0.66	0.999
NOL6	RRP9	4437451	4433566	9606.ENSP00000297990	9606.ENSP00000232888	0	0	0	0	0.873	0.866	0.9	0.748	0.999
NOP56	RRP9	4445513	4433566	9606.ENSP00000370589	9606.ENSP00000232888	0	0	0	0	0.883	0.796	0.9	0.881	0.999
UTP15	MPHOSPH	4437338	4433887	9606.ENSP00000296792	9606.ENSP00000244230	0	0	0	0	0.827	0.961	0.9	0.741	0.999
LTV1	NOB1	4442915	4435851	9606.ENSP00000356548	9606.ENSP00000268802	0	0	0	0	0.799	0.825	0.9	0.917	0.999
HEATR1	PWP2	4442698	4436922	9606.ENSP00000355541	9606.ENSP00000291576	0	0	0	0	0.835	0.737	0.9	0.801	0.999
CIRH1A	MPHOSPH	4439903	4433887	9606.ENSP00000327179	9606.ENSP00000244230	0	0	0	0	0.841	0.798	0.9	0.776	0.999
WDR46	UTP18	4444423	4433308	9606.ENSP00000363746	9606.ENSP00000225298	0	0	0	0	0.924	0.784	0.9	0.731	0.999
HEATR1	UTP6	4442698	4434930	9606.ENSP00000355541	9606.ENSP00000261708	0	0	0	0	0.844	0.797	0.9	0.836	0.999
TBL3	NOP14	4450478	4448347	9606.ENSP00000454836	9606.ENSP00000405068	0	0	0	0	0.9	0.792	0.9	0.595	0.999
HEATR1	DCAF13	4442698	4437420	9606.ENSP00000355541	9606.ENSP00000297579	0	0	0	0	0.879	0.796	0.9	0.657	0.999
BMS1	HEATR1	4444407	4442698	9606.ENSP00000363642	9606.ENSP00000355541	0	0	0	0	0.9	0.797	0.9	0.738	0.999
TBL3	DCAF13	4450478	4437420	9606.ENSP00000454836	9606.ENSP00000297579	0	0	0	0.55	0.9	0.968	0.9	0.718	0.999
NOP56	CIRH1A	4445513	4439903	9606.ENSP00000370589	9606.ENSP00000327179	0	0	0	0	0.891	0.798	0.9	0.655	0.999
PDCD11	NOL6	4443395	4437451	9606.ENSP00000358812	9606.ENSP00000297990	0	0	0	0	0.893	0.731	0.9	0.795	0.999
WDR36	CIRH1A	4449235	4439903	9606.ENSP00000423067	9606.ENSP00000327179	0	0	0	0	0.905	0.798	0.9	0.782	0.999
WDR46	NOC4L	4444423	4440054	9606.ENSP00000363746	9606.ENSP00000328854	0	0	0	0	0.889	0.788	0.9	0.7	0.999
WDR36	KRR1	4449235	4433449	9606.ENSP00000423067	9606.ENSP00000229214	0	0	0	0	0.636	0.961	0.9	0.678	0.999
UTP11L	UTP18	4444078	4433308	9606.ENSP00000362105	9606.ENSP00000225298	0	0	0	0	0.834	0.79	0.9	0.747	0.999
MPHOSPH	RRP9	4433887	4433566	9606.ENSP00000244230	9606.ENSP00000232888	0	0	0	0	0.857	0.798	0.9	0.798	0.999
TSR1	RIOK2	4437751	4436521	9606.ENSP00000301364	9606.ENSP00000283109	0	0	0	0	0.821	0.97	0.9	0.917	0.999
WDR75	UTP18	4438919	4433308	9606.ENSP00000314193	9606.ENSP00000225298	0	0	0	0	0.891	0.789	0.9	0.7	0.999
NOL6	FBL	4437451	4433181	9606.ENSP00000297990	9606.ENSP00000221801	0	0	0	0	0.753	0.952	0.9	0.718	0.999
WDR3	PNO1	4438389	4435289	9606.ENSP00000308179	9606.ENSP00000263657	0	0	0	0	0.859	0.985	0.9	0.382	0.999
NOP14	UTP18	4448347	4433308	9606.ENSP00000405068	9606.ENSP00000225298	0	0	0	0	0.87	0.798	0.9	0.761	0.999
WDR75	UTP15	4438919	4437338	9606.ENSP00000314193	9606.ENSP00000296792	0	0	0	0	0.923	0.798	0.9	0.907	0.999
CIRH1A	UTP15	4439903	4437338	9606.ENSP00000327179	9606.ENSP00000296792	0	0	0	0	0.882	0.929	0.9	0.916	0.999
WDR36	UTP15	4449235	4437338	9606.ENSP00000423067	9606.ENSP00000296792	0	0	0	0	0.924	0.958	0.9	0.807	0.999
NOP14	NOP58	4448347	4435411	9606.ENSP00000405068	9606.ENSP00000264279	0	0	0	0	0.878	0.798	0.9	0.678	0.999

Gene 1	Gene 2	node1_string_internal_id	node2_string_internal_id	node1_external_id	node2_external_id	neighborhood_on_chromosome	gene_fusion	phylogenetic_cooccurrence	homology	coexpression	experimentally_determined_interaction	database_annotated	automated_textmining	combined_score
WDR36	WDR3	4449235	4438389	9606.ENSP00000423067	9606.ENSP00000308179	0	0	0	0.543	0.914	0.959	0.9	0.828	0.999
BMS1	UTP3	4444407	4434386	9606.ENSP00000363642	9606.ENSP00000254803	0	0	0	0	0.905	0.784	0.9	0.791	0.999
NOC4L	WDR3	4440054	4438389	9606.ENSP00000328854	9606.ENSP00000308179	0	0	0	0	0.891	0.788	0.9	0.669	0.999
WDR36	NOP58	4449235	4435411	9606.ENSP00000423067	9606.ENSP00000264279	0	0	0	0	0.89	0.961	0.9	0.366	0.999
BMS1	NOP58	4444407	4435411	9606.ENSP00000363642	9606.ENSP00000264279	0	0	0	0	0.887	0.798	0.9	0.619	0.999
TBL3	PWP2	4450478	4436922	9606.ENSP00000454836	9606.ENSP00000291576	0	0	0	0.545	0.817	0.958	0.9	0.864	0.999
WDR36	PWP2	4449235	4436922	9606.ENSP00000423067	9606.ENSP00000291576	0	0	0	0.543	0.868	0.964	0.9	0.831	0.999
NOP14	BMS1	4448347	4444407	9606.ENSP00000405068	9606.ENSP00000363642	0	0	0	0	0.909	0.798	0.9	0.803	0.999
RRP9	UTP18	4433566	4433308	9606.ENSP00000232888	9606.ENSP00000225298	0	0	0	0	0.921	0.784	0.9	0.761	0.999
NOP58	RRP9	4435411	4433566	9606.ENSP00000264279	9606.ENSP00000232888	0	0	0	0	0.879	0.798	0.9	0.882	0.999
RIOK2	NOB1	4436521	4435851	9606.ENSP00000283109	9606.ENSP00000268802	0	0	0	0	0.849	0.932	0.9	0.919	0.999
NOC4L	KRR1	4440054	4433449	9606.ENSP00000328854	9606.ENSP00000229214	0	0	0	0	0.841	0.965	0.9	0.427	0.999
TSR1	BYSL	4437751	4433499	9606.ENSP00000301364	9606.ENSP00000230340	0	0	0	0	0.908	0.99	0.9	0.915	0.999
UTP6	RRP9	4434930	4433566	9606.ENSP00000261708	9606.ENSP00000232888	0	0	0	0	0.905	0.798	0.9	0.787	0.999
NOB1	PNO1	4435851	4435289	9606.ENSP00000268802	9606.ENSP00000263657	0	0	0	0	0.86	0.972	0.9	0.917	0.999
DCAF13	UTP6	4437420	4434930	9606.ENSP00000297579	9606.ENSP00000261708	0	0	0	0	0.891	0.798	0.9	0.663	0.999
WDR46	UTP3	4444423	4434386	9606.ENSP00000363746	9606.ENSP00000254803	0	0	0	0	0.909	0.816	0.9	0.719	0.999
WDR3	UTP6	4438389	4434930	9606.ENSP00000308179	9606.ENSP00000261708	0	0	0	0	0.896	0.798	0.9	0.854	0.999
PDCD11	CIRH1A	4443395	4439903	9606.ENSP00000358812	9606.ENSP00000327179	0	0	0	0	0.891	0.788	0.9	0.745	0.999
NOP56	BMS1	4445513	4444407	9606.ENSP00000370589	9606.ENSP00000363642	0	0	0	0	0.901	0.791	0.9	0.602	0.999
WDR36	UTP18	4449235	4433308	9606.ENSP00000423067	9606.ENSP00000225298	0	0	0	0	0.891	0.798	0.9	0.881	0.999
TBL3	KRR1	4450478	4433449	9606.ENSP00000454836	9606.ENSP00000229214	0	0	0	0	0.838	0.972	0.9	0.537	0.999
EMG1	BYSL	4450965	4433499	9606.ENSP00000470560	9606.ENSP00000230340	0	0	0	0	0.866	0.847	0.9	0.677	0.999
CIRH1A	NOL6	4439903	4437451	9606.ENSP00000327179	9606.ENSP00000297990	0	0	0	0	0.882	0.788	0.9	0.783	0.999
WDR43	WDR46	4447102	4444423	9606.ENSP00000384302	9606.ENSP00000363746	0	0	0	0	0.895	0.805	0.9	0.721	0.999
WDR36	MPHOSPH	4449235	4433887	9606.ENSP00000423067	9606.ENSP00000244230	0	0	0	0	0.857	0.957	0.9	0.684	0.999
NOP56	FBL	4445513	4433181	9606.ENSP00000370589	9606.ENSP00000221801	0	0	0	0	0.939	0.961	0.9	0.95	0.999
NOP14	PDCD11	4448347	4443395	9606.ENSP00000405068	9606.ENSP00000358812	0	0	0	0	0.895	0.731	0.9	0.761	0.999
LTV1	TSR1	4442915	4437751	9606.ENSP00000356548	9606.ENSP00000301364	0	0	0	0	0.856	0.992	0.9	0.916	0.999
WDR43	WDR75	4447102	4438919	9606.ENSP00000384302	9606.ENSP00000314193	0	0	0	0	0.908	0.788	0.9	0.92	0.999
WDR36	WDR43	4449235	4447102	9606.ENSP00000423067	9606.ENSP00000384302	0	0	0	0	0.906	0.788	0.9	0.768	0.999
CIRH1A	RRP9	4439903	4433566	9606.ENSP00000327179	9606.ENSP00000232888	0	0	0	0	0.911	0.798	0.9	0.734	0.999
UTP3	UTP18	4434386	4433308	9606.ENSP00000254803	9606.ENSP00000225298	0	0	0	0	0.802	0.798	0.9	0.852	0.999
WDR46	UTP11L	4444423	4444078	9606.ENSP00000363746	9606.ENSP00000362105	0	0	0	0	0.893	0.779	0.9	0.659	0.999
IMP4	MPHOSPH	4434687	4433887	9606.ENSP00000259239	9606.ENSP00000244230	0	0	0	0	0.854	0.969	0.9	0.426	0.999
WDR46	CIRH1A	4444423	4439903	9606.ENSP00000363746	9606.ENSP00000327179	0	0	0	0	0.891	0.798	0.9	0.72	0.999
PDCD11	MPHOSPH	4443395	4433887	9606.ENSP00000358812	9606.ENSP00000244230	0	0	0	0	0.853	0.788	0.9	0.788	0.999
BMS1	UTP6	4444407	4434930	9606.ENSP00000363642	9606.ENSP00000261708	0	0	0	0	0.876	0.798	0.9	0.692	0.999
DIEXF	UTP3	4449004	4434386	9606.ENSP00000419005	9606.ENSP00000254803	0	0	0	0	0.848	0.692	0.9	0.813	0.999
HEATR1	WDR3	4442698	4438389	9606.ENSP00000355541	9606.ENSP00000308179	0	0	0	0	0.907	0.784	0.9	0.75	0.999
NOB1	BYSL	4435851	4433499	9606.ENSP00000268802	9606.ENSP00000230340	0	0	0	0	0.889	0.918	0.9	0.916	0.999
WDR75	RRP9	4438919	4433566	9606.ENSP00000314193	9606.ENSP00000232888	0	0	0	0	0.893	0.796	0.9	0.63	0.999
NOP14	MPHOSPH	4448347	4433887	9606.ENSP00000405068	9606.ENSP00000244230	0	0	0	0	0.89	0.798	0.9	0.853	0.999
NOL6	MPHOSPH	4437451	4433887	9606.ENSP00000297990	9606.ENSP00000244230	0	0	0	0	0.845	0.969	0.9	0.724	0.999

Gene 1	Gene 2	node1_string_internal_id	node2_string_internal_id	node1_external_id	node2_external_id	neighborhood_on_chromosome	gene_fusion	phylogenetic_cooccurrence	homology	coexpression	experimentally_determined_interaction	database_annotated	automated_textmining	combined_score
UTP6	UTP3	4434930	4434386	9606.ENSP00000261708	9606.ENSP00000254803	0	0	0	0	0.858	0.798	0.9	0.788	0.999
PDCD11	RRP9	4443395	4433566	9606.ENSP00000358812	9606.ENSP00000232888	0	0	0	0	0.864	0.733	0.9	0.757	0.999
WDR36	UTP6	4449235	4434930	9606.ENSP00000423067	9606.ENSP00000261708	0	0	0	0	0.875	0.869	0.9	0.884	0.999
CIRH1A	NOP58	4439903	4435411	9606.ENSP00000327179	9606.ENSP00000264279	0	0	0	0	0.888	0.798	0.9	0.722	0.999
WDR46	NOL6	4444423	4437451	9606.ENSP00000363746	9606.ENSP00000297990	0	0	0	0	0.885	0.805	0.9	0.647	0.999
TBL3	NOL6	4450478	4437451	9606.ENSP00000454836	9606.ENSP00000297990	0	0	0	0	0.882	0.788	0.9	0.794	0.999
NOL6	PWP2	4437451	4436922	9606.ENSP00000297990	9606.ENSP00000291576	0	0	0	0	0.837	0.733	0.9	0.827	0.999
BMS1	PNO1	4444407	4435289	9606.ENSP00000363642	9606.ENSP00000263657	0	0	0	0	0.853	0.792	0.9	0.771	0.999
NOP56	UTP18	4445513	4433308	9606.ENSP00000370589	9606.ENSP00000225298	0	0	0	0	0.865	0.866	0.9	0.66	0.999
UTP11L	UTP3	4444078	4434386	9606.ENSP00000362105	9606.ENSP00000254803	0	0	0	0	0.858	0.797	0.9	0.781	0.999
WDR46	HEATR1	4444423	4442698	9606.ENSP00000363746	9606.ENSP00000355541	0	0	0	0	0.885	0.798	0.9	0.727	0.999
NOP56	NOL6	4445513	4437451	9606.ENSP00000370589	9606.ENSP00000297990	0	0	0	0	0.877	0.927	0.9	0.718	0.999
NOP14	BYSL	4448347	4433499	9606.ENSP00000405068	9606.ENSP00000230340	0	0	0	0	0.897	0.819	0.9	0.568	0.999
HEATR1	CIRH1A	4442698	4439903	9606.ENSP00000355541	9606.ENSP00000327179	0	0	0	0	0.901	0.798	0.9	0.896	0.999
TBL3	RRP9	4450478	4433566	9606.ENSP00000454836	9606.ENSP00000232888	0	0	0.287	0.579	0.903	0.968	0.9	0.747	0.999
WDR3	RRP9	4438389	4433566	9606.ENSP00000308179	9606.ENSP00000232888	0	0	0.34	0.581	0.903	0.965	0.9	0.535	0.999
BMS1	UTP15	4444407	4437338	9606.ENSP00000363642	9606.ENSP00000296792	0	0	0	0	0.876	0.791	0.9	0.743	0.999
LTV1	BYSL	4442915	4433499	9606.ENSP00000356548	9606.ENSP00000230340	0	0	0	0	0.811	0.984	0.9	0.948	0.999
NOP56	PDCD11	4445513	4443395	9606.ENSP00000370589	9606.ENSP00000358812	0	0	0	0	0.896	0.731	0.9	0.703	0.999
WDR36	NOL6	4449235	4437451	9606.ENSP00000423067	9606.ENSP00000297990	0	0	0	0	0.916	0.736	0.9	0.75	0.999
PNO1	KRR1	4435289	4433449	9606.ENSP00000263657	9606.ENSP00000229214	0	0	0	0	0.853	0.784	0.9	0.792	0.999
CIRH1A	UTP6	4439903	4434930	9606.ENSP00000327179	9606.ENSP00000261708	0	0	0	0	0.899	0.798	0.9	0.833	0.999
WDR36	HEATR1	4449235	4442698	9606.ENSP00000423067	9606.ENSP00000355541	0	0	0	0	0.91	0.796	0.9	0.81	0.999
NOP56	IMP4	4445513	4434687	9606.ENSP00000370589	9606.ENSP00000259239	0	0	0	0	0.847	0.8	0.9	0.711	0.999
NOC4L	BYSL	4440054	4433499	9606.ENSP00000328854	9606.ENSP00000230340	0	0	0	0	0.901	0.81	0.9	0.752	0.999
NOP56	NOP58	4445513	4435411	9606.ENSP00000370589	9606.ENSP00000264279	0	0	0	0.873	0.944	0.973	0.9	0.974	0.999
NOP58	KRR1	4435411	4433449	9606.ENSP00000264279	9606.ENSP00000229214	0	0	0	0	0.875	0.962	0.9	0.392	0.999
NOP14	UTP6	4448347	4434930	9606.ENSP00000405068	9606.ENSP00000261708	0	0	0	0	0.894	0.798	0.9	0.705	0.999
NOP14	UTP3	4448347	4434386	9606.ENSP00000405068	9606.ENSP00000254803	0	0	0	0	0.898	0.798	0.9	0.679	0.999
NOC4L	RRP9	4440054	4433566	9606.ENSP00000328854	9606.ENSP00000232888	0	0	0	0	0.902	0.744	0.9	0.676	0.999
NOP56	HEATR1	4445513	4442698	9606.ENSP00000370589	9606.ENSP00000355541	0	0	0	0	0.895	0.797	0.9	0.727	0.999
HEATR1	WDR75	4442698	4438919	9606.ENSP00000355541	9606.ENSP00000314193	0	0	0	0	0.893	0.798	0.9	0.915	0.999
DCAF13	NOP58	4437420	4435411	9606.ENSP00000297579	9606.ENSP00000264279	0	0	0	0	0.88	0.972	0.9	0.513	0.999
HEATR1	UTP18	4442698	4433308	9606.ENSP00000355541	9606.ENSP00000225298	0	0	0	0	0.905	0.797	0.9	0.826	0.999
HEATR1	NOL6	4442698	4437451	9606.ENSP00000355541	9606.ENSP00000297990	0	0	0	0	0.906	0.736	0.9	0.788	0.999
RIOK2	PNO1	4436521	4435289	9606.ENSP00000283109	9606.ENSP00000263657	0	0	0	0	0.85	0.95	0.9	0.916	0.999
WDR3	UTP18	4438389	4433308	9606.ENSP00000308179	9606.ENSP00000225298	0	0	0	0	0.899	0.798	0.9	0.84	0.999
NOP14	NOP56	4448347	4445513	9606.ENSP00000405068	9606.ENSP00000370589	0	0	0	0	0.889	0.784	0.9	0.695	0.999
BMS1	MPHOSPH	4444407	4433887	9606.ENSP00000363642	9606.ENSP00000244230	0	0	0	0	0.896	0.797	0.9	0.84	0.999
NOP56	KRR1	4445513	4433449	9606.ENSP00000370589	9606.ENSP00000229214	0	0	0	0	0.846	0.962	0.9	0.61	0.999
IMP3	PWP2	4439890	4436922	9606.ENSP00000326981	9606.ENSP00000291576	0	0	0	0	0.534	0.958	0.9	0.747	0.999
RRP9	FBL	4433566	4433181	9606.ENSP00000232888	9606.ENSP00000221801	0	0	0	0	0.804	0.827	0.9	0.832	0.999
WDR43	FBL	4447102	4433181	9606.ENSP00000384302	9606.ENSP00000221801	0	0	0	0	0.849	0.789	0.9	0.544	0.998
PWP2	UTP18	4436922	4433308	9606.ENSP00000291576	9606.ENSP00000225298	0	0	0	0	0.724	0.742	0.9	0.864	0.998

Gene 1	Gene 2	node1_string_internal_id	node2_string_internal_id	node1_external_id	node2_external_id	neighborhood_on_chromosome	gene_fusion	phylogenetic_cooccurrence	homology	coexpression	experimentally_determined_interaction	database_annotated	automated_textmining	combined_score
WDR46	PNO1	4444423	4435289	9606.ENSP00000363746	9606.ENSP00000263657	0	0	0	0	0.856	0.784	0.9	0.649	0.998
RCL1	RRP9	4445592	4433566	9606.ENSP00000371169	9606.ENSP00000232888	0	0	0	0	0.823	0.796	0.9	0.741	0.998
WDR75	PNO1	4438919	4435289	9606.ENSP00000314193	9606.ENSP00000263657	0	0	0	0	0.854	0.796	0.9	0.439	0.998
WDR75	NOL6	4438919	4437451	9606.ENSP00000314193	9606.ENSP00000297990	0	0	0	0	0.872	0.736	0.9	0.628	0.998
WDR3	NOP58	4438389	4435411	9606.ENSP00000308179	9606.ENSP00000264279	0	0	0	0	0.897	0.798	0.9	0.325	0.998
CIRH1A	FBL	4439903	4433181	9606.ENSP00000327179	9606.ENSP00000221801	0	0	0	0	0.834	0.811	0.9	0.671	0.998
TBL3	PNO1	4450478	4435289	9606.ENSP00000454836	9606.ENSP00000263657	0	0	0	0	0.854	0.784	0.9	0.55	0.998
PDCD11	UTP15	4443395	4437338	9606.ENSP00000358812	9606.ENSP00000296792	0	0	0	0	0.843	0.721	0.9	0.679	0.998
BMS1	WDR75	4444407	4438919	9606.ENSP00000363642	9606.ENSP00000314193	0	0	0	0	0.881	0.797	0.9	0.614	0.998
TBL3	IMP4	4450478	4434687	9606.ENSP00000454836	9606.ENSP00000259239	0	0	0	0	0.859	0.798	0.9	0.426	0.998
IMP4	FBL	4434687	4433181	9606.ENSP00000259239	9606.ENSP00000221801	0	0	0	0	0.71	0.838	0.9	0.668	0.998
NOP58	UTP6	4435411	4434930	9606.ENSP00000264279	9606.ENSP00000261708	0	0	0	0	0.881	0.798	0.9	0.453	0.998
UTP11L	DCAF13	4444078	4437420	9606.ENSP00000362105	9606.ENSP00000297579	0	0	0	0	0.877	0.779	0.9	0.634	0.998
MPHOSPH	FBL	4433887	4433181	9606.ENSP00000244230	9606.ENSP00000221801	0	0	0	0	0.543	0.865	0.9	0.784	0.998
PDCD11	PWP2	4443395	4436922	9606.ENSP00000358812	9606.ENSP00000291576	0	0	0	0	0.812	0.733	0.9	0.794	0.998
NOL6	UTP15	4437451	4437338	9606.ENSP00000297990	9606.ENSP00000296792	0	0	0	0	0.857	0.651	0.9	0.756	0.998
NOP14	WDR43	4448347	4447102	9606.ENSP00000405068	9606.ENSP00000384302	0	0	0	0	0.899	0.651	0.9	0.739	0.998
UTP15	BYSL	4437338	4433499	9606.ENSP00000296792	9606.ENSP00000230340	0	0	0	0	0.897	0.766	0.9	0.588	0.998
EMG1	RRP9	4450965	4433566	9606.ENSP00000470560	9606.ENSP00000232888	0	0	0	0	0.852	0.785	0.9	0.572	0.998
NOL6	IMP4	4437451	4434687	9606.ENSP00000297990	9606.ENSP00000259239	0	0	0	0	0.822	0.788	0.9	0.545	0.998
EMG1	IMP4	4450965	4434687	9606.ENSP00000470560	9606.ENSP00000259239	0	0	0	0	0.871	0.784	0.9	0.426	0.998
NOP14	KRR1	4448347	4433449	9606.ENSP00000405068	9606.ENSP00000229214	0	0	0	0	0.86	0.797	0.9	0.638	0.998
RRP9	KRR1	4433566	4433449	9606.ENSP00000232888	9606.ENSP00000229214	0	0	0	0	0.85	0.797	0.9	0.703	0.998
NOP56	WDR75	4445513	4438919	9606.ENSP00000370589	9606.ENSP00000314193	0	0	0	0	0.85	0.784	0.9	0.652	0.998
WDR36	FBL	4449235	4433181	9606.ENSP00000423067	9606.ENSP00000221801	0	0	0	0	0.751	0.886	0.9	0.652	0.998
EMG1	NOP58	4450965	4435411	9606.ENSP00000470560	9606.ENSP00000264279	0	0	0	0	0.862	0.798	0.9	0.562	0.998
BMS1	DCAF13	4444407	4437420	9606.ENSP00000363642	9606.ENSP00000297579	0	0	0	0	0.873	0.792	0.9	0.625	0.998
DCAF13	BYSL	4437420	4433499	9606.ENSP00000297579	9606.ENSP00000230340	0	0	0	0	0.862	0.765	0.9	0.518	0.998
CIRH1A	IMP3	4439903	4439890	9606.ENSP00000327179	9606.ENSP00000326981	0	0	0	0	0.811	0.784	0.9	0.637	0.998
IMP3	UTP6	4439890	4434930	9606.ENSP00000326981	9606.ENSP00000261708	0	0	0	0	0.836	0.796	0.9	0.661	0.998
WDR36	BYSL	4449235	4433499	9606.ENSP00000423067	9606.ENSP00000230340	0	0	0	0	0.883	0.788	0.9	0.334	0.998
EMG1	DCAF13	4450965	4437420	9606.ENSP00000470560	9606.ENSP00000297579	0	0	0	0	0.859	0.784	0.9	0.53	0.998
PNO1	UTP18	4435289	4433308	9606.ENSP00000263657	9606.ENSP00000225298	0	0	0	0	0.886	0.792	0.9	0.624	0.998
IMP4	KRR1	4434687	4433449	9606.ENSP00000259239	9606.ENSP00000229214	0	0	0	0	0.868	0.784	0.9	0.426	0.998
NOP56	BYSL	4445513	4433499	9606.ENSP00000370589	9606.ENSP00000230340	0	0	0	0	0.88	0.73	0.9	0.64	0.998
UTP11L	BYSL	4444078	4433499	9606.ENSP00000362105	9606.ENSP00000230340	0	0	0	0	0.797	0.72	0.9	0.749	0.998
BMS1	CIRH1A	4444407	4439903	9606.ENSP00000363642	9606.ENSP00000327179	0	0	0	0	0.884	0.798	0.9	0.582	0.998
HEATR1	PNO1	4442698	4435289	9606.ENSP00000355541	9606.ENSP00000263657	0	0	0	0	0.834	0.784	0.9	0.585	0.998
NOL6	KRR1	4437451	4433449	9606.ENSP00000297990	9606.ENSP00000229214	0	0	0	0	0.509	0.788	0.9	0.876	0.998
EMG1	PNO1	4450965	4435289	9606.ENSP00000470560	9606.ENSP00000263657	0.045	0	0	0	0.88	0.784	0.9	0.46	0.998
CIRH1A	UTP3	4439903	4434386	9606.ENSP00000327179	9606.ENSP00000254803	0	0	0	0	0.843	0.798	0.9	0.525	0.998
DCAF13	MPHOSPH	4437420	4433887	9606.ENSP00000297579	9606.ENSP00000244230	0	0	0	0	0.832	0.798	0.9	0.577	0.998
HEATR1	FBL	4442698	4433181	9606.ENSP00000355541	9606.ENSP00000221801	0	0	0	0	0.708	0.836	0.9	0.673	0.998
HEATR1	NOC4L	4442698	4440054	9606.ENSP00000355541	9606.ENSP00000328854	0	0	0	0	0.868	0.788	0.9	0.426	0.998

Gene 1	Gene 2	node1_string_internal_id	node2_string_internal_id	node1_external_id	node2_external_id	neighborhood_on_chromosome	gene_fusion	phylogenetic_cooccurrence	homology	coexpression	experimentally_determined_interaction	database_annotated	automated_textmining	combined_score
WDR46	PWP2	4444423	4436922	9606.ENSP00000363746	9606.ENSP00000291576	0	0	0	0	0.816	0.737	0.9	0.646	0.998
NOP56	WDR46	4445513	4444423	9606.ENSP00000370589	9606.ENSP00000363746	0	0	0	0	0.883	0.797	0.9	0.471	0.998
BYSL	UTP18	4433499	4433308	9606.ENSP00000230340	9606.ENSP00000225298	0	0	0	0	0.889	0.651	0.9	0.618	0.998
NOP14	DCAF13	4448347	4437420	9606.ENSP00000405068	9606.ENSP00000297579	0	0	0	0	0.886	0.787	0.9	0.56	0.998
BMS1	WDR3	4444407	4438389	9606.ENSP00000363642	9606.ENSP00000308179	0	0	0	0	0.896	0.797	0.9	0.574	0.998
NOC4L	WDR75	4440054	4438919	9606.ENSP00000328854	9606.ENSP00000314193	0	0	0	0	0.884	0.788	0.9	0.426	0.998
EMG1	TBL3	4450965	4450478	9606.ENSP00000470560	9606.ENSP00000454836	0	0	0	0	0.837	0.784	0.9	0.584	0.998
UTP15	RRP9	4437338	4433566	9606.ENSP00000296792	9606.ENSP00000232888	0	0	0.398	0.557	0.89	0.796	0.9	0.756	0.998
WDR36	PNO1	4449235	4435289	9606.ENSP00000423067	9606.ENSP00000263657	0	0	0	0	0.851	0.784	0.9	0.511	0.998
TBL3	NOC4L	4450478	4440054	9606.ENSP00000454836	9606.ENSP00000328854	0	0	0	0	0.897	0.788	0.9	0.426	0.998
UTP15	FBL	4437338	4433181	9606.ENSP00000296792	9606.ENSP00000221801	0	0	0	0	0.73	0.877	0.9	0.687	0.998
UTP18	FBL	4433308	4433181	9606.ENSP00000225298	9606.ENSP00000221801	0	0	0	0	0.767	0.814	0.9	0.644	0.998
RPS6	PNO1	4445386	4435289	9606.ENSP00000369757	9606.ENSP00000263657	0	0	0	0	0.697	0.96	0.9	0.11	0.998
NOC4L	DCAF13	4440054	4437420	9606.ENSP00000328854	9606.ENSP00000297579	0	0	0	0	0.855	0.788	0.9	0.427	0.998
UTP11L	UTP6	4444078	4434930	9606.ENSP00000362105	9606.ENSP00000261708	0	0	0	0	0.868	0.779	0.9	0.591	0.998
EMG1	WDR36	4450965	4449235	9606.ENSP00000470560	9606.ENSP00000423067	0	0	0	0	0.812	0.784	0.9	0.678	0.998
WDR43	UTP6	4447102	4434930	9606.ENSP00000384302	9606.ENSP00000261708	0	0	0	0	0.891	0.651	0.9	0.758	0.998
NOP56	UTP6	4445513	4434930	9606.ENSP00000370589	9606.ENSP00000261708	0	0	0	0	0.868	0.784	0.9	0.636	0.998
PDCD11	UTP6	4443395	4434930	9606.ENSP00000358812	9606.ENSP00000261708	0	0	0	0	0.834	0.737	0.9	0.7	0.998
WDR3	UTP3	4438389	4434386	9606.ENSP00000308179	9606.ENSP00000254803	0	0	0	0	0.844	0.784	0.9	0.686	0.998
NOC4L	IMP4	4440054	4434687	9606.ENSP00000328854	9606.ENSP00000259239	0	0	0	0	0.874	0.788	0.9	0.426	0.998
NOC4L	UTP18	4440054	4433308	9606.ENSP00000328854	9606.ENSP00000225298	0	0	0	0	0.88	0.788	0.9	0.426	0.998
EMG1	UTP11L	4450965	4444078	9606.ENSP00000470560	9606.ENSP00000362105	0	0	0	0	0.855	0.779	0.9	0.572	0.998
WDR36	NOP14	4449235	4448347	9606.ENSP00000423067	9606.ENSP00000405068	0	0	0	0	0.891	0.785	0.9	0.587	0.998
NOP14	RCL1	4448347	4445592	9606.ENSP00000405068	9606.ENSP00000371169	0	0	0	0	0.661	0.796	0.9	0.756	0.998
EMG1	IMP3	4450965	4439890	9606.ENSP00000470560	9606.ENSP00000326981	0	0	0	0	0.884	0.784	0.9	0.426	0.998
UTP15	IMP4	4437338	4434687	9606.ENSP00000296792	9606.ENSP00000259239	0	0	0	0	0.854	0.784	0.9	0.453	0.998
DCAF13	UTP3	4437420	4434386	9606.ENSP00000297579	9606.ENSP00000254803	0	0	0	0	0.838	0.784	0.9	0.605	0.998
BMS1	NOL6	4444407	4437451	9606.ENSP00000363642	9606.ENSP00000297990	0	0	0	0	0.89	0.708	0.9	0.643	0.998
WDR75	MPHOSPH	4438919	4433887	9606.ENSP00000314193	9606.ENSP00000244230	0	0	0	0	0.804	0.798	0.9	0.637	0.998
WDR75	NOP58	4438919	4435411	9606.ENSP00000314193	9606.ENSP00000264279	0	0	0	0	0.912	0.798	0.9	0.387	0.998
WDR36	NOC4L	4449235	4440054	9606.ENSP00000423067	9606.ENSP00000328854	0	0	0	0	0.888	0.788	0.9	0.538	0.998
EMG1	CIRH1A	4450965	4439903	9606.ENSP00000470560	9606.ENSP00000327179	0	0	0	0	0.808	0.798	0.9	0.754	0.998
UTP6	KRR1	4434930	4433449	9606.ENSP00000261708	9606.ENSP00000229214	0	0	0	0	0.848	0.798	0.9	0.692	0.998
EMG1	NOC4L	4450965	4440054	9606.ENSP00000470560	9606.ENSP00000328854	0	0	0	0	0.872	0.788	0.9	0.537	0.998
UTP3	KRR1	4434386	4433449	9606.ENSP00000254803	9606.ENSP00000229214	0	0	0	0	0.887	0.798	0.9	0.432	0.998
PDCD11	WDR3	4443395	4438389	9606.ENSP00000358812	9606.ENSP00000308179	0	0	0	0	0.897	0.737	0.9	0.616	0.998
NOP14	UTP11L	4448347	4444078	9606.ENSP00000405068	9606.ENSP00000362105	0	0	0	0	0.861	0.779	0.9	0.605	0.998
WDR3	DCAF13	4438389	4437420	9606.ENSP00000308179	9606.ENSP00000297579	0	0	0	0	0.898	0.784	0.9	0.451	0.998
CIRH1A	BYSL	4439903	4433499	9606.ENSP00000327179	9606.ENSP00000230340	0	0	0	0	0.87	0.788	0.9	0.457	0.998
WDR3	IMP4	4438389	4434687	9606.ENSP00000308179	9606.ENSP00000259239	0	0	0	0	0.879	0.784	0.9	0.553	0.998
PDCD11	KRR1	4443395	4433449	9606.ENSP00000358812	9606.ENSP00000229214	0	0	0	0	0.827	0.73	0.9	0.717	0.998
UTP6	BYSL	4434930	4433499	9606.ENSP00000261708	9606.ENSP00000230340	0	0	0	0	0.877	0.766	0.9	0.552	0.998
NOP58	UTP3	4435411	4434386	9606.ENSP00000264279	9606.ENSP00000254803	0	0	0	0	0.874	0.798	0.9	0.395	0.998

Gene 1	Gene 2	node1_string_internal_id	node2_string_internal_id	node1_external_id	node2_external_id	neighborhood_on_chromosome	gene_fusion	phylogenetic_cooccurrence	homology	coexpression	experimentally_determined_interaction	database_annotated	automated_textmining	combined_score
WDR46	WDR3	4444423	4438389	9606.ENSP00000363746	9606.ENSP00000308179	0	0	0	0	0.9	0.797	0.9	0.56	0.998
WDR46	RRP9	4444423	4433566	9606.ENSP00000363746	9606.ENSP00000232888	0	0	0	0	0.907	0.795	0.9	0.488	0.998
NOC4L	MPHOSPH	4440054	4433887	9606.ENSP00000328854	9606.ENSP00000244230	0	0	0	0	0.855	0.788	0.9	0.426	0.998
PDCD11	UTP18	4443395	4433308	9606.ENSP00000358812	9606.ENSP00000225298	0	0	0	0	0.86	0.699	0.9	0.704	0.998
WDR36	UTP3	4449235	4434386	9606.ENSP00000423067	9606.ENSP00000254803	0	0	0	0	0.836	0.798	0.9	0.707	0.998
MPHOSPH	KRR1	4433887	4433449	9606.ENSP00000244230	9606.ENSP00000229214	0	0	0	0	0.847	0.797	0.9	0.636	0.998
WDR75	WDR3	4438919	4438389	9606.ENSP00000314193	9606.ENSP00000308179	0	0	0	0.542	0.899	0.784	0.9	0.652	0.998
WDR43	BYSL	4447102	4433499	9606.ENSP00000384302	9606.ENSP00000230340	0	0	0	0	0.906	0.72	0.9	0.343	0.998
EMG1	UTP6	4450965	4434930	9606.ENSP00000470560	9606.ENSP00000261708	0	0	0	0	0.841	0.796	0.9	0.681	0.998
EMG1	NOP56	4450965	4445513	9606.ENSP00000470560	9606.ENSP00000370589	0	0	0	0	0.844	0.784	0.9	0.647	0.998
UTP11L	IMP4	4444078	4434687	9606.ENSP00000362105	9606.ENSP00000259239	0	0	0	0	0.893	0.779	0.9	0.369	0.998
PDCD11	NOP58	4443395	4435411	9606.ENSP00000358812	9606.ENSP00000264279	0	0	0	0	0.9	0.788	0.9	0.558	0.998
HEATR1	MPHOSPH	4442698	4433887	9606.ENSP00000355541	9606.ENSP00000244230	0	0	0	0	0.824	0.798	0.9	0.74	0.998
NOC4L	CIRH1A	4440054	4439903	9606.ENSP00000328854	9606.ENSP00000327179	0	0	0	0	0.888	0.788	0.9	0.6	0.998
BMS1	UTP11L	4444407	4444078	9606.ENSP00000363642	9606.ENSP00000362105	0	0	0	0	0.849	0.779	0.9	0.734	0.998
WDR43	NOP56	4447102	4445513	9606.ENSP00000384302	9606.ENSP00000370589	0	0	0	0	0.91	0.651	0.9	0.706	0.998
WDR36	PDCD11	4449235	4443395	9606.ENSP00000423067	9606.ENSP00000358812	0	0	0	0	0.904	0.731	0.9	0.59	0.998
UTP11L	KRR1	4444078	4433449	9606.ENSP00000362105	9606.ENSP00000229214	0	0	0	0	0.867	0.779	0.9	0.658	0.998
NOP56	UTP15	4445513	4437338	9606.ENSP00000370589	9606.ENSP00000296792	0	0	0	0	0.867	0.784	0.9	0.679	0.998
EMG1	UTP15	4450965	4437338	9606.ENSP00000470560	9606.ENSP00000296792	0	0	0	0	0.85	0.785	0.9	0.63	0.998
TBL3	BYSL	4450478	4433499	9606.ENSP00000454836	9606.ENSP00000230340	0	0	0	0	0.891	0.73	0.9	0.562	0.998
DIEXF	MPHOSPH	4449004	4433887	9606.ENSP00000419005	9606.ENSP00000244230	0	0	0	0	0.817	0.716	0.9	0.741	0.998
WDR75	UTP6	4438919	4434930	9606.ENSP00000314193	9606.ENSP00000261708	0	0	0	0	0.892	0.796	0.9	0.572	0.998
BMS1	KRR1	4444407	4433449	9606.ENSP00000363642	9606.ENSP00000229214	0	0	0	0	0.869	0.797	0.9	0.55	0.998
PDCD11	NOC4L	4443395	4440054	9606.ENSP00000358812	9606.ENSP00000328854	0	0	0	0	0.853	0.788	0.9	0.617	0.998
IMP3	KRR1	4439890	4433449	9606.ENSP00000326981	9606.ENSP00000229214	0	0	0	0	0.848	0.798	0.9	0.426	0.998
PWP2	NOP58	4436922	4435411	9606.ENSP00000291576	9606.ENSP00000264279	0	0	0	0	0.811	0.742	0.9	0.693	0.998
TBL3	CIRH1A	4450478	4439903	9606.ENSP00000454836	9606.ENSP00000327179	0	0	0	0.545	0.899	0.798	0.9	0.845	0.998
DCAF13	IMP4	4437420	4434687	9606.ENSP00000297579	9606.ENSP00000259239	0	0	0	0	0.89	0.784	0.9	0.426	0.998
CIRH1A	IMP4	4439903	4434687	9606.ENSP00000327179	9606.ENSP00000259239	0	0	0	0	0.85	0.798	0.9	0.426	0.998
WDR43	BMS1	4447102	4444407	9606.ENSP00000384302	9606.ENSP00000363642	0	0	0	0	0.902	0.651	0.9	0.621	0.998
UTP11L	PNO1	4444078	4435289	9606.ENSP00000362105	9606.ENSP00000263657	0	0	0	0	0.857	0.779	0.9	0.681	0.998
TBL3	PDCD11	4450478	4443395	9606.ENSP00000454836	9606.ENSP00000358812	0	0	0	0	0.895	0.73	0.9	0.574	0.998
WDR3	UTP15	4438389	4437338	9606.ENSP00000308179	9606.ENSP00000296792	0	0	0.379	0.568	0.906	0.792	0.9	0.795	0.998
NOP14	PWP2	4448347	4436922	9606.ENSP00000405068	9606.ENSP00000291576	0	0	0	0	0.817	0.737	0.9	0.701	0.998
WDR43	UTP3	4447102	4434386	9606.ENSP00000384302	9606.ENSP00000254803	0	0	0	0	0.862	0.651	0.9	0.713	0.998
PNO1	UTP3	4435289	4434386	9606.ENSP00000263657	9606.ENSP00000254803	0	0	0	0	0.837	0.784	0.9	0.537	0.998
UTP11L	UTP15	4444078	4437338	9606.ENSP00000362105	9606.ENSP00000296792	0	0	0	0	0.712	0.779	0.9	0.734	0.998
RPS3A	PNO1	4441583	4435289	9606.ENSP00000346050	9606.ENSP00000263657	0	0	0	0	0.759	0.951	0.9	0.072	0.998
WDR75	UTP3	4438919	4434386	9606.ENSP00000314193	9606.ENSP00000254803	0	0	0	0	0.843	0.784	0.9	0.654	0.998
PNO1	RRP9	4435289	4433566	9606.ENSP00000263657	9606.ENSP00000232888	0	0	0	0	0.891	0.784	0.9	0.371	0.998
PDCD11	WDR75	4443395	4438919	9606.ENSP00000358812	9606.ENSP00000314193	0	0	0	0	0.888	0.788	0.9	0.585	0.998
RCL1	WDR3	4445592	4438389	9606.ENSP00000371169	9606.ENSP00000308179	0	0	0	0	0.853	0.784	0.9	0.583	0.998
WDR43	IMP3	4447102	4439890	9606.ENSP00000384302	9606.ENSP00000326981	0	0	0	0	0.811	0.788	0.9	0.664	0.998

Gene 1	Gene 2	node1_string_internal_id	node2_string_internal_id	node1_external_id	node2_external_id	neighborhood_on_chromosome	gene_fusion	phylogenetic_cooccurrence	homology	coexpression	experimentally_determined_interaction	database_annotated	automated_textmining	combined_score
WDR36	IMP4	4449235	4434687	9606.ENSP00000423067	9606.ENSP00000259239	0	0	0	0	0.853	0.798	0.9	0.5	0.998
EMG1	FBL	4450965	4433181	9606.ENSP00000470560	9606.ENSP00000221801	0	0	0	0	0.842	0.784	0.9	0.71	0.998
NOC4L	UTP6	4440054	4434930	9606.ENSP00000328854	9606.ENSP00000261708	0	0	0	0	0.894	0.788	0.9	0.426	0.998
WDR46	IMP4	4444423	4434687	9606.ENSP00000363746	9606.ENSP00000259239	0	0	0	0	0.899	0.805	0.9	0.426	0.998
RRP9	BYSL	4433566	4433499	9606.ENSP00000232888	9606.ENSP00000230340	0	0	0	0	0.899	0.73	0.9	0.435	0.998
EMG1	WDR3	4450965	4438389	9606.ENSP00000470560	9606.ENSP00000308179	0	0	0	0	0.826	0.784	0.9	0.541	0.998
PWP2	BYSL	4436922	4433499	9606.ENSP00000291576	9606.ENSP00000230340	0	0	0	0	0.777	0.832	0.9	0.676	0.998
TBL3	IMP3	4450478	4439890	9606.ENSP00000454836	9606.ENSP00000326981	0	0	0	0	0.842	0.784	0.9	0.654	0.998
UTP6	IMP4	4434930	4434687	9606.ENSP00000261708	9606.ENSP00000259239	0	0	0	0	0.896	0.792	0.9	0.426	0.998
NOP14	IMP4	4448347	4434687	9606.ENSP00000405068	9606.ENSP00000259239	0	0	0	0	0.856	0.792	0.9	0.426	0.998
WDR46	PDCD11	4444423	4443395	9606.ENSP00000363746	9606.ENSP00000358812	0	0	0	0	0.88	0.73	0.9	0.61	0.998
IMP3	UTP18	4439890	4433308	9606.ENSP00000326981	9606.ENSP00000225298	0	0	0	0	0.811	0.798	0.9	0.681	0.998
WDR3	MPHOSPH	4438389	4433887	9606.ENSP00000308179	9606.ENSP00000244230	0	0	0	0	0.851	0.798	0.9	0.652	0.998
UTP3	RRP9	4434386	4433566	9606.ENSP00000254803	9606.ENSP00000232888	0	0	0	0	0.863	0.784	0.9	0.598	0.998
PNO1	UTP6	4435289	4434930	9606.ENSP00000263657	9606.ENSP00000261708	0	0	0	0	0.866	0.784	0.9	0.564	0.998
NOP58	UTP18	4435411	4433308	9606.ENSP00000264279	9606.ENSP00000225298	0	0	0	0	0.885	0.798	0.9	0.582	0.998
NOP14	WDR75	4448347	4438919	9606.ENSP00000405068	9606.ENSP00000314193	0	0	0	0	0.883	0.792	0.9	0.608	0.998
WDR43	WDR3	4447102	4438389	9606.ENSP00000384302	9606.ENSP00000308179	0	0	0	0.546	0.909	0.72	0.9	0.727	0.998
RCL1	UTP15	4445592	4437338	9606.ENSP00000371169	9606.ENSP00000296792	0	0	0	0	0.851	0.784	0.9	0.598	0.998
TBL3	WDR75	4450478	4438919	9606.ENSP00000454836	9606.ENSP00000314193	0	0	0	0	0.875	0.784	0.9	0.5	0.998
WDR46	MPHOSPH	4444423	4433887	9606.ENSP00000363746	9606.ENSP00000244230	0	0	0	0	0.873	0.798	0.9	0.467	0.998
IMP4	UTP18	4434687	4433308	9606.ENSP00000259239	9606.ENSP00000225298	0	0	0	0	0.869	0.784	0.9	0.551	0.998
NOP56	WDR3	4445513	4438389	9606.ENSP00000370589	9606.ENSP00000308179	0	0	0	0	0.903	0.802	0.9	0.479	0.998
NOP58	BYSL	4435411	4433499	9606.ENSP00000264279	9606.ENSP00000230340	0	0	0	0	0.883	0.788	0.9	0.303	0.998
HEATR1	BYSL	4442698	4433499	9606.ENSP00000355541	9606.ENSP00000230340	0	0	0	0	0.882	0.73	0.9	0.619	0.998
NOP56	UTP3	4445513	4434386	9606.ENSP00000370589	9606.ENSP00000254803	0	0	0	0	0.876	0.784	0.9	0.664	0.998
NOC4L	UTP15	4440054	4437338	9606.ENSP00000328854	9606.ENSP00000296792	0	0	0	0	0.859	0.788	0.9	0.527	0.998
PWP2	FBL	4436922	4433181	9606.ENSP00000291576	9606.ENSP00000221801	0	0	0	0	0.731	0.795	0.9	0.654	0.997
NOP56	PNO1	4445513	4435289	9606.ENSP00000370589	9606.ENSP00000263657	0	0	0	0	0.834	0.784	0.9	0.364	0.997
NOP14	IMP3	4448347	4439890	9606.ENSP00000405068	9606.ENSP00000326981	0	0	0	0	0.813	0.784	0.9	0.426	0.997
NOC4L	DHX37	4440054	4438659	9606.ENSP00000328854	9606.ENSP00000311135	0	0	0	0	0.868	0.692	0.9	0.427	0.997
HEATR1	KRR1	4442698	4433449	9606.ENSP00000355541	9606.ENSP00000229214	0	0	0	0	0.466	0.797	0.9	0.769	0.997
EMG1	WDR46	4450965	4444423	9606.ENSP00000470560	9606.ENSP00000363746	0	0	0	0	0.764	0.785	0.9	0.597	0.997
IMP4	BYSL	4434687	4433499	9606.ENSP00000259239	9606.ENSP00000230340	0	0	0	0	0.888	0.788	0.9	0.221	0.997
UTP15	UTP3	4437338	4434386	9606.ENSP00000296792	9606.ENSP00000254803	0	0	0	0	0.667	0.784	0.9	0.676	0.997
PWP2	IMP4	4436922	4434687	9606.ENSP00000291576	9606.ENSP00000259239	0	0	0	0	0.781	0.74	0.9	0.591	0.997
PNO1	IMP4	4435289	4434687	9606.ENSP00000263657	9606.ENSP00000259239	0	0	0	0	0.893	0.784	0.9	0.226	0.997
DCAF13	PWP2	4437420	4436922	9606.ENSP00000297579	9606.ENSP00000291576	0	0	0	0	0.804	0.738	0.9	0.54	0.997
UTP11L	IMP3	4444078	4439890	9606.ENSP00000362105	9606.ENSP00000326981	0	0	0	0	0.854	0.779	0.9	0.221	0.997
IMP3	PNO1	4439890	4435289	9606.ENSP00000326981	9606.ENSP00000263657	0	0	0	0	0.873	0.784	0.9	0.269	0.997
WDR75	BYSL	4438919	4433499	9606.ENSP00000314193	9606.ENSP00000230340	0	0	0	0	0.864	0.72	0.9	0.305	0.997
PDCD11	FBL	4443395	4433181	9606.ENSP00000358812	9606.ENSP00000221801	0	0	0	0	0.738	0.757	0.9	0.682	0.997
IMP3	BYSL	4439890	4433499	9606.ENSP00000326981	9606.ENSP00000230340	0	0	0	0	0.85	0.788	0.9	0.221	0.997
EMG1	WDR75	4450965	4438919	9606.ENSP00000470560	9606.ENSP00000314193	0	0	0	0	0.67	0.784	0.9	0.642	0.997

Gene 1	Gene 2	node1_string_internal_id	node2_string_internal_id	node1_external_id	node2_external_id	neighborhood_on_chromosome	gene_fusion	phylogenetic_cooccurrence	homology	coexpression	experimentally_determined_interaction	database_annotated	automated_textmining	combined_score
BMS1	PWP2	4444407	4436922	9606.ENSP00000363642	9606.ENSP00000291576	0	0	0	0	0.812	0.742	0.9	0.597	0.997
UTP11L	RRP9	4444078	4433566	9606.ENSP00000362105	9606.ENSP00000232888	0	0	0	0	0.85	0.779	0.9	0.421	0.997
RCL1	DCAF13	4445592	4437420	9606.ENSP00000371169	9606.ENSP00000297579	0	0	0	0	0.833	0.784	0.9	0.473	0.997
PWP2	KRR1	4436922	4433449	9606.ENSP00000291576	9606.ENSP00000229214	0	0	0	0	0.664	0.737	0.9	0.783	0.997
TBL3	RCL1	4450478	4445592	9606.ENSP00000454836	9606.ENSP00000371169	0	0	0	0	0.732	0.784	0.9	0.653	0.997
WDR46	IMP3	4444423	4439890	9606.ENSP00000363746	9606.ENSP00000326981	0	0	0	0	0.839	0.784	0.9	0.426	0.997
DHX37	DDX49	4438659	4434008	9606.ENSP00000311135	9606.ENSP00000247003	0	0	0	0	0.846	0.157	0.9	0.839	0.997
NOP58	IMP4	4435411	4434687	9606.ENSP00000264279	9606.ENSP00000259239	0	0	0	0	0.851	0.798	0.9	0.408	0.997
WDR3	KRR1	4438389	4433449	9606.ENSP00000308179	9606.ENSP00000229214	0	0	0	0	0.839	0.792	0.9	0.458	0.997
PNO1	FBL	4435289	4433181	9606.ENSP00000263657	9606.ENSP00000221801	0	0	0	0	0.732	0.868	0.9	0.434	0.997
RCL1	CIRH1A	4445592	4439903	9606.ENSP00000371169	9606.ENSP00000327179	0	0	0	0	0.725	0.798	0.9	0.621	0.997
BMS1	IMP4	4444407	4434687	9606.ENSP00000363642	9606.ENSP00000259239	0	0	0	0	0.855	0.797	0.9	0.387	0.997
DHX37	MPHOSPH	4438659	4433887	9606.ENSP00000311135	9606.ENSP00000244230	0	0	0	0	0.83	0.692	0.9	0.559	0.997
CIRH1A	PNO1	4439903	4435289	9606.ENSP00000327179	9606.ENSP00000263657	0	0	0	0	0.856	0.798	0.9	0.338	0.997
BMS1	NOC4L	4444407	4440054	9606.ENSP00000363642	9606.ENSP00000328854	0	0	0	0	0.858	0.788	0.9	0.221	0.997
WDR43	NOC4L	4447102	4440054	9606.ENSP00000384302	9606.ENSP00000328854	0	0	0	0	0.873	0.788	0.9	0.221	0.997
WDR43	PDCD11	4447102	4443395	9606.ENSP00000384302	9606.ENSP00000358812	0	0	0	0	0.861	0.721	0.9	0.451	0.997
NOC4L	PNO1	4440054	4435289	9606.ENSP00000328854	9606.ENSP00000263657	0	0	0	0	0.875	0.788	0.9	0.221	0.997
WDR75	FBL	4438919	4433181	9606.ENSP00000314193	9606.ENSP00000221801	0	0	0	0	0.569	0.827	0.9	0.648	0.997
UTP15	PNO1	4437338	4435289	9606.ENSP00000296792	9606.ENSP00000263657	0	0	0	0	0.867	0.784	0.9	0.358	0.997
HEATR1	UTP3	4442698	4434386	9606.ENSP00000355541	9606.ENSP00000254803	0	0	0	0	0.758	0.798	0.9	0.592	0.997
WDR3	FBL	4438389	4433181	9606.ENSP00000308179	9606.ENSP00000221801	0	0	0	0	0.728	0.837	0.9	0.515	0.997
UTP15	KRR1	4437338	4433449	9606.ENSP00000296792	9606.ENSP00000229214	0	0	0	0	0.511	0.875	0.9	0.624	0.997
WDR75	IMP4	4438919	4434687	9606.ENSP00000314193	9606.ENSP00000259239	0	0	0	0	0.846	0.796	0.9	0.426	0.997
WDR46	FBL	4444423	4433181	9606.ENSP00000363746	9606.ENSP00000221801	0	0	0	0	0.747	0.797	0.9	0.58	0.997
NOP58	PNO1	4435411	4435289	9606.ENSP00000264279	9606.ENSP00000263657	0	0	0	0	0.855	0.798	0.9	0.245	0.997
NOC4L	IMP3	4440054	4439890	9606.ENSP00000328854	9606.ENSP00000326981	0	0	0	0	0.867	0.708	0.9	0.426	0.997
DDX49	BYSL	4434008	4433499	9606.ENSP00000247003	9606.ENSP00000230340	0	0	0	0	0.917	0.129	0.9	0.714	0.997
EMG1	KRR1	4450965	4433449	9606.ENSP00000470560	9606.ENSP00000229214	0.045	0	0	0	0.65	0.797	0.9	0.629	0.997
IMP3	DCAF13	4439890	4437420	9606.ENSP00000326981	9606.ENSP00000297579	0	0	0	0	0.847	0.784	0.9	0.426	0.997
DCAF13	FBL	4437420	4433181	9606.ENSP00000297579	9606.ENSP00000221801	0	0	0	0	0.489	0.885	0.9	0.631	0.997
PDCD11	DCAF13	4443395	4437420	9606.ENSP00000358812	9606.ENSP00000297579	0	0	0	0	0.82	0.721	0.9	0.638	0.997
WDR43	RRP9	4447102	4433566	9606.ENSP00000384302	9606.ENSP00000232888	0	0	0	0.556	0.923	0.651	0.9	0.685	0.997
UTP15	DDX49	4437338	4434008	9606.ENSP00000296792	9606.ENSP00000247003	0	0	0	0	0.921	0.05	0.9	0.682	0.997
NOP14	PNO1	4448347	4435289	9606.ENSP00000405068	9606.ENSP00000263657	0	0	0	0	0.832	0.784	0.9	0.392	0.997
PDCD11	UTP3	4443395	4434386	9606.ENSP00000358812	9606.ENSP00000254803	0	0	0	0	0.862	0.651	0.9	0.541	0.997
WDR36	DIEXF	4449235	4449004	9606.ENSP00000423067	9606.ENSP00000419005	0	0	0	0	0.89	0.692	0.9	0.391	0.997
TBL3	UTP3	4450478	4434386	9606.ENSP00000454836	9606.ENSP00000254803	0	0	0	0	0.852	0.784	0.9	0.439	0.997
RCL1	UTP18	4445592	4433308	9606.ENSP00000371169	9606.ENSP00000225298	0	0	0	0	0.72	0.784	0.9	0.645	0.997
MPHOSPH	BYSL	4433887	4433499	9606.ENSP00000244230	9606.ENSP00000230340	0	0	0	0	0.85	0.76	0.9	0.402	0.997
UTP11L	NOC4L	4444078	4440054	9606.ENSP00000362105	9606.ENSP00000328854	0	0	0	0	0.841	0.788	0.9	0.221	0.997
WDR46	NOP58	4444423	4435411	9606.ENSP00000363746	9606.ENSP00000264279	0	0	0	0	0.864	0.798	0.9	0.147	0.997
RCL1	MPHOSPH	4445592	4433887	9606.ENSP00000371169	9606.ENSP00000244230	0	0	0	0	0.509	0.784	0.9	0.801	0.997
PNO1	MPHOSPH	4435289	4433887	9606.ENSP00000263657	9606.ENSP00000244230	0	0	0	0	0.843	0.784	0.9	0.322	0.997

Gene 1	Gene 2	node1_string_internal_id	node2_string_internal_id	node1_external_id	node2_external_id	neighborhood_on_chromosome	gene_fusion	phylogenetic_cooccurrence	homology	coexpression	experimentally_determined_interaction	database_annotated	automated_textmining	combined_score
KRR1	UTP18	4433449	4433308	9606.ENSP00000229214	9606.ENSP00000225298	0	0	0	0	0.664	0.798	0.9	0.737	0.997
RCL1	UTP6	4445592	4434930	9606.ENSP00000371169	9606.ENSP00000261708	0	0	0	0	0.752	0.791	0.9	0.571	0.997
CIRH1A	KRR1	4439903	4433449	9606.ENSP00000327179	9606.ENSP00000229214	0	0	0	0	0.651	0.798	0.9	0.664	0.997
NOC4L	FBL	4440054	4433181	9606.ENSP00000328854	9606.ENSP00000221801	0	0	0	0	0.766	0.789	0.9	0.485	0.997
NOL6	DCAF13	4437451	4437420	9606.ENSP00000297990	9606.ENSP00000297579	0	0	0	0	0.826	0.651	0.9	0.62	0.997
NOL6	BYSL	4437451	4433499	9606.ENSP00000297990	9606.ENSP00000230340	0	0	0	0	0.696	0.788	0.9	0.642	0.997
WDR43	DCAF13	4447102	4437420	9606.ENSP00000384302	9606.ENSP00000297579	0	0	0	0	0.875	0.651	0.9	0.508	0.997
UTP11L	MPHOSPH	4444078	4433887	9606.ENSP00000362105	9606.ENSP00000244230	0	0	0	0	0.848	0.797	0.9	0.281	0.997
CIRH1A	DHX37	4439903	4438659	9606.ENSP00000327179	9606.ENSP00000311135	0	0	0	0	0.862	0.692	0.9	0.439	0.997
UTP6	FBL	4434930	4433181	9606.ENSP00000261708	9606.ENSP00000221801	0	0	0	0	0.743	0.791	0.9	0.609	0.997
UTP11L	WDR3	4444078	4438389	9606.ENSP00000362105	9606.ENSP00000308179	0	0	0	0	0.694	0.779	0.9	0.616	0.997
BYSL	FBL	4433499	4433181	9606.ENSP00000230340	9606.ENSP00000221801	0	0	0	0	0.696	0.839	0.9	0.638	0.997
EMG1	HEATR1	4450965	4442698	9606.ENSP00000470560	9606.ENSP00000355541	0	0	0	0	0.606	0.796	0.9	0.679	0.997
DHX37	NOL6	4438659	4437451	9606.ENSP00000311135	9606.ENSP00000297990	0	0	0	0	0.855	0.692	0.9	0.541	0.997
NOP56	PWP2	4445513	4436922	9606.ENSP00000370589	9606.ENSP00000291576	0	0	0	0	0.751	0.737	0.9	0.652	0.997
UTP3	BYSL	4434386	4433499	9606.ENSP00000254803	9606.ENSP00000230340	0	0	0	0	0.846	0.788	0.9	0.457	0.997
RCL1	IMP3	4445592	4439890	9606.ENSP00000371169	9606.ENSP00000326981	0	0	0	0	0.821	0.784	0.9	0.505	0.997
NOC4L	NOL6	4440054	4437451	9606.ENSP00000328854	9606.ENSP00000297990	0	0	0	0	0.856	0.736	0.9	0.507	0.997
IMP3	UTP15	4439890	4437338	9606.ENSP00000326981	9606.ENSP00000296792	0	0	0	0	0.679	0.784	0.9	0.664	0.997
DHX37	UTP3	4438659	4434386	9606.ENSP00000311135	9606.ENSP00000254803	0	0	0	0	0.863	0.692	0.9	0.246	0.996
RIOK2	KRR1	4436521	4433449	9606.ENSP00000283109	9606.ENSP00000229214	0	0	0	0	0.843	0.403	0.9	0.627	0.996
NOC4L	PWP2	4440054	4436922	9606.ENSP00000328854	9606.ENSP00000291576	0	0	0	0	0.788	0.733	0.9	0.42	0.996
BMS1	FBL	4444407	4433181	9606.ENSP00000363642	9606.ENSP00000221801	0	0	0	0	0.364	0.837	0.9	0.666	0.996
IMP3	WDR3	4439890	4438389	9606.ENSP00000326981	9606.ENSP00000308179	0	0	0	0	0.701	0.784	0.9	0.512	0.996
IMP3	WDR75	4439890	4438919	9606.ENSP00000326981	9606.ENSP00000314193	0	0	0	0	0.701	0.798	0.9	0.426	0.996
PWP2	PNO1	4436922	4435289	9606.ENSP00000291576	9606.ENSP00000263657	0	0	0	0	0.748	0.735	0.9	0.511	0.996
WDR43	IMP4	4447102	4434687	9606.ENSP00000384302	9606.ENSP00000259239	0	0	0	0	0.855	0.651	0.9	0.426	0.996
DIEXF	NOL6	4449004	4437451	9606.ENSP00000419005	9606.ENSP00000297990	0	0	0	0	0.853	0.692	0.9	0.281	0.996
UTP3	DDX49	4434386	4434008	9606.ENSP00000254803	9606.ENSP00000247003	0	0	0	0	0.855	0.692	0.9	0.27	0.996
NOP14	DHX37	4448347	4438659	9606.ENSP00000405068	9606.ENSP00000311135	0	0	0	0	0.892	0.412	0.9	0.534	0.996
WDR36	IMP3	4449235	4439890	9606.ENSP00000423067	9606.ENSP00000326981	0	0	0	0	0.603	0.784	0.9	0.612	0.996
RPS6	FBL	4445386	4433181	9606.ENSP00000369757	9606.ENSP00000221801	0	0	0	0	0.653	0.828	0.9	0.443	0.996
WDR43	PWP2	4447102	4436922	9606.ENSP00000384302	9606.ENSP00000291576	0	0	0	0	0.821	0.414	0.9	0.738	0.996
RCL1	PWP2	4445592	4436922	9606.ENSP00000371169	9606.ENSP00000291576	0	0	0	0	0.656	0.735	0.9	0.679	0.996
EMG1	BMS1	4450965	4444407	9606.ENSP00000470560	9606.ENSP00000363642	0	0	0	0	0.656	0.792	0.9	0.599	0.996
IMP4	DDX49	4434687	4434008	9606.ENSP00000259239	9606.ENSP00000247003	0	0	0	0	0.928	0.176	0.9	0.401	0.996
DIEXF	RRP9	4449004	4433566	9606.ENSP00000419005	9606.ENSP00000232888	0	0	0	0	0.885	0.144	0.9	0.662	0.996
RCL1	NOC4L	4445592	4440054	9606.ENSP00000371169	9606.ENSP00000328854	0	0	0	0	0.793	0.788	0.9	0.221	0.996
WDR43	RCL1	4447102	4445592	9606.ENSP00000384302	9606.ENSP00000371169	0	0	0	0	0.69	0.651	0.9	0.699	0.996
RPS6	LTV1	4445386	4442915	9606.ENSP00000369757	9606.ENSP00000356548	0	0	0	0	0.087	0.96	0.9	0.058	0.996
EMG1	WDR43	4450965	4447102	9606.ENSP00000470560	9606.ENSP00000384302	0	0	0	0	0.783	0.651	0.9	0.561	0.996
WDR43	PNO1	4447102	4435289	9606.ENSP00000384302	9606.ENSP00000263657	0	0	0	0	0.868	0.651	0.9	0.417	0.996
NOP56	NOC4L	4445513	4440054	9606.ENSP00000370589	9606.ENSP00000328854	0	0	0	0	0.836	0.708	0.9	0.426	0.996
PWP2	UTP3	4436922	4434386	9606.ENSP00000291576	9606.ENSP00000254803	0	0	0	0	0.72	0.742	0.9	0.595	0.996

Gene 1	Gene 2	node1_string_internal_id	node2_string_internal_id	node1_external_id	node2_external_id	neighborhood_on_chromosome	gene_fusion	phylogenetic_cooccurrence	homology	coexpression	experimentally_determined_interaction	database_annotated	automated_textmining	combined_score
NOL6	UTP3	4437451	4434386	9606.ENSP00000297990	9606.ENSP00000254803	0	0	0	0	0.686	0.788	0.9	0.56	0.996
RPS6	BYSL	4445386	4433499	9606.ENSP00000369757	9606.ENSP00000230340	0	0	0	0	0.247	0.919	0.9	0.548	0.996
LTV1	RPS3A	4442915	4441583	9606.ENSP00000356548	9606.ENSP00000346050	0	0	0	0	0.081	0.96	0.9	0.142	0.996
PDCD11	TSR1	4443395	4437751	9606.ENSP00000358812	9606.ENSP00000301364	0	0	0	0	0.899	0	0.9	0.681	0.996
WDR75	PWP2	4438919	4436922	9606.ENSP00000314193	9606.ENSP00000291576	0	0	0	0	0.781	0.737	0.9	0.477	0.996
RCL1	WDR75	4445592	4438919	9606.ENSP00000371169	9606.ENSP00000314193	0	0	0	0	0.475	0.784	0.9	0.709	0.996
PDCD11	IMP4	4443395	4434687	9606.ENSP00000358812	9606.ENSP00000259239	0	0	0	0	0.654	0.788	0.9	0.533	0.996
WDR75	KRR1	4438919	4433449	9606.ENSP00000314193	9606.ENSP00000229214	0	0	0	0	0.637	0.797	0.9	0.618	0.996
RCL1	TSR1	4445592	4437751	9606.ENSP00000371169	9606.ENSP00000301364	0	0	0	0	0.697	0	0.9	0.849	0.995
DIEXF	IMP4	4449004	4434687	9606.ENSP00000419005	9606.ENSP00000259239	0	0	0	0	0.851	0.692	0.9	0	0.995
TBL3	DDX49	4450478	4434008	9606.ENSP00000454836	9606.ENSP00000247003	0	0	0	0	0.916	0.053	0.9	0.48	0.995
NOC4L	TSR1	4440054	4437751	9606.ENSP00000328854	9606.ENSP00000301364	0	0	0	0	0.89	0	0.9	0.649	0.995
RCL1	UTP3	4445592	4434386	9606.ENSP00000371169	9606.ENSP00000254803	0	0	0	0	0.253	0.784	0.9	0.735	0.995
TSR1	KRR1	4437751	4433449	9606.ENSP00000301364	9606.ENSP00000229214	0	0	0	0	0.828	0.395	0.9	0.598	0.995
NOP14	FBL	4448347	4433181	9606.ENSP00000405068	9606.ENSP00000221801	0	0	0	0	0.419	0.836	0.9	0.563	0.995
LTV1	KRR1	4442915	4433449	9606.ENSP00000356548	9606.ENSP00000229214	0	0	0	0	0.873	0.366	0.9	0.532	0.995
UTP15	PWP2	4437338	4436922	9606.ENSP00000296792	9606.ENSP00000291576	0	0	0	0.564	0.787	0.737	0.9	0.781	0.995
DHX37	NOP58	4438659	4435411	9606.ENSP00000311135	9606.ENSP00000264279	0	0	0	0	0.837	0.692	0.9	0.245	0.995
PDCD11	PNO1	4443395	4435289	9606.ENSP00000358812	9606.ENSP00000263657	0	0	0	0	0.731	0.651	0.9	0.566	0.995
EMG1	RCL1	4450965	4445592	9606.ENSP00000470560	9606.ENSP00000371169	0	0	0	0	0.675	0.788	0.9	0.399	0.995
HEATR1	IMP4	4442698	4434687	9606.ENSP00000355541	9606.ENSP00000259239	0	0	0	0	0.692	0.798	0.9	0.426	0.995
NOP56	IMP3	4445513	4439890	9606.ENSP00000370589	9606.ENSP00000326981	0	0	0	0	0.516	0.784	0.9	0.587	0.995
RCL1	FBL	4445592	4433181	9606.ENSP00000371169	9606.ENSP00000221801	0	0	0	0	0.358	0.835	0.9	0.659	0.995
DDX49	RRP9	4434008	4433566	9606.ENSP00000247003	9606.ENSP00000232888	0	0	0	0	0.92	0	0.9	0.474	0.995
RCL1	NOP56	4445592	4445513	9606.ENSP00000371169	9606.ENSP00000370589	0.043	0	0	0	0.455	0.784	0.9	0.657	0.995
RCL1	NOP58	4445592	4435411	9606.ENSP00000371169	9606.ENSP00000264279	0.043	0	0	0	0.495	0.798	0.9	0.642	0.995
TSR1	DDX49	4437751	4434008	9606.ENSP00000301364	9606.ENSP00000247003	0	0	0	0	0.919	0.054	0.9	0.427	0.995
PNO1	DDX49	4435289	4434008	9606.ENSP00000263657	9606.ENSP00000247003	0	0	0	0	0.865	0.461	0.9	0.427	0.995
IMP3	FBL	4439890	4433181	9606.ENSP00000326981	9606.ENSP00000221801	0	0	0	0	0.543	0.793	0.9	0.585	0.995
BMS1	DHX37	4444407	4438659	9606.ENSP00000363642	9606.ENSP00000311135	0	0	0	0	0.908	0.308	0.9	0.412	0.995
HEATR1	TSR1	4442698	4437751	9606.ENSP00000355541	9606.ENSP00000301364	0	0	0	0	0.896	0	0.9	0.58	0.995
BMS1	TSR1	4444407	4437751	9606.ENSP00000363642	9606.ENSP00000301364	0	0	0	0.566	0.906	0.308	0.9	0.944	0.995
RCL1	PDCD11	4445592	4443395	9606.ENSP00000371169	9606.ENSP00000358812	0	0	0	0	0.268	0.651	0.9	0.831	0.995
UTP11L	NOP58	4444078	4435411	9606.ENSP00000362105	9606.ENSP00000264279	0	0	0	0	0.756	0.797	0.9	0.132	0.995
NOC4L	DDX49	4440054	4434008	9606.ENSP00000328854	9606.ENSP00000247003	0	0	0	0	0.916	0.064	0.9	0.539	0.995
DDX49	UTP18	4434008	4433308	9606.ENSP00000247003	9606.ENSP00000225298	0	0	0	0	0.929	0.171	0.9	0.391	0.995
DHX37	RRP9	4438659	4433566	9606.ENSP00000311135	9606.ENSP00000232888	0	0	0	0	0.857	0.318	0.9	0.568	0.995
TBL3	UTP11L	4450478	4444078	9606.ENSP00000454836	9606.ENSP00000362105	0	0	0	0	0.725	0.779	0.9	0.325	0.995
WDR43	TSR1	4447102	4437751	9606.ENSP00000384302	9606.ENSP00000301364	0	0	0	0	0.924	0	0.9	0.357	0.994
WDR36	UTP11L	4449235	4444078	9606.ENSP00000423067	9606.ENSP00000362105	0	0	0	0	0.658	0.779	0.9	0.326	0.994
PDCD11	DHX37	4443395	4438659	9606.ENSP00000358812	9606.ENSP00000311135	0.043	0	0	0	0.894	0.133	0.9	0.458	0.994
WDR43	KRR1	4447102	4433449	9606.ENSP00000384302	9606.ENSP00000229214	0	0	0	0	0.68	0.708	0.9	0.515	0.994
WDR46	DDX49	4444423	4434008	9606.ENSP00000363746	9606.ENSP00000247003	0	0	0	0	0.937	0.064	0.9	0.221	0.994
DHX37	BYSL	4438659	4433499	9606.ENSP00000311135	9606.ENSP00000230340	0	0	0	0	0.849	0.284	0.9	0.556	0.994

Gene 1	Gene 2	node1_string_internal_id	node2_string_internal_id	node1_external_id	node2_external_id	neighborhood_on_chromosome	gene_fusion	phylogenetic_cooccurrence	homology	coexpression	experimentally_determined_interaction	database_annotated	automated_textmining	combined_score
TBL3	DHX37	4450478	4438659	9606.ENSP00000454836	9606.ENSP00000311135	0	0	0	0	0.89	0.057	0.9	0.55	0.994
WDR43	UTP11L	4447102	4444078	9606.ENSP00000384302	9606.ENSP00000362105	0	0	0	0	0.704	0.651	0.9	0.523	0.994
HEATR1	IMP3	4442698	4439890	9606.ENSP00000355541	9606.ENSP00000326981	0	0	0	0	0.278	0.796	0.9	0.65	0.994
NOP56	UTP11L	4445513	4444078	9606.ENSP00000370589	9606.ENSP00000362105	0	0	0	0	0.676	0.779	0.9	0.33	0.994
EMG1	PWP2	4450965	4436922	9606.ENSP00000470560	9606.ENSP00000291576	0	0	0	0	0.522	0.737	0.9	0.588	0.994
WDR36	RCL1	4449235	4445592	9606.ENSP00000423067	9606.ENSP00000371169	0	0	0	0	0.543	0.794	0.9	0.452	0.994
RCL1	HEATR1	4445592	4442698	9606.ENSP00000371169	9606.ENSP00000355541	0	0	0	0	0.228	0.792	0.9	0.714	0.994
DHX37	UTP6	4438659	4434930	9606.ENSP00000311135	9606.ENSP00000261708	0	0	0	0	0.699	0.692	0.9	0.445	0.994
PWP2	DDX49	4436922	4434008	9606.ENSP00000291576	9606.ENSP00000247003	0	0.002	0	0	0.83	0.285	0.9	0.565	0.994
TSR1	NOL6	4437751	4437451	9606.ENSP00000301364	9606.ENSP00000297990	0	0	0	0	0.903	0.087	0.9	0.49	0.994
EMG1	NOL6	4450965	4437451	9606.ENSP00000470560	9606.ENSP00000297990	0	0	0	0	0.321	0.748	0.9	0.708	0.994
KRR1	FBL	4433449	4433181	9606.ENSP00000229214	9606.ENSP00000221801	0	0	0	0	0.358	0.803	0.9	0.611	0.994
RCL1	WDR46	4445592	4444423	9606.ENSP00000371169	9606.ENSP00000363746	0	0	0	0	0.663	0.784	0.9	0.389	0.994
UTP3	FBL	4434386	4433181	9606.ENSP00000254803	9606.ENSP00000221801	0	0	0	0	0.394	0.802	0.9	0.575	0.994
NOL6	PNO1	4437451	4435289	9606.ENSP00000297990	9606.ENSP00000263657	0	0	0	0	0.524	0.736	0.9	0.64	0.994
UTP11L	HEATR1	4444078	4442698	9606.ENSP00000362105	9606.ENSP00000355541	0	0	0	0	0.337	0.779	0.9	0.665	0.994
DIEXF	PDCD11	4449004	4443395	9606.ENSP00000419005	9606.ENSP00000358812	0	0	0	0	0.852	0	0.9	0.621	0.993
UTP6	DDX49	4434930	4434008	9606.ENSP00000261708	9606.ENSP00000247003	0	0	0	0	0.926	0.055	0.9	0.221	0.993
UTP11L	CIRH1A	4444078	4439903	9606.ENSP00000362105	9606.ENSP00000327179	0	0	0	0	0.561	0.797	0.9	0.356	0.993
BMS1	LTV1	4444407	4442915	9606.ENSP00000363642	9606.ENSP00000356548	0	0	0	0	0.857	0.064	0.9	0.573	0.993
IMP3	NOP58	4439890	4435411	9606.ENSP00000326981	9606.ENSP00000264279	0	0	0	0	0.588	0.798	0.9	0.312	0.993
UTP11L	DDX49	4444078	4434008	9606.ENSP00000362105	9606.ENSP00000247003	0	0	0	0	0.927	0.05	0.9	0.221	0.993
WDR36	DHX37	4449235	4438659	9606.ENSP00000423067	9606.ENSP00000311135	0	0	0	0	0.881	0.187	0.9	0.428	0.993
PDCD11	NOB1	4443395	4435851	9606.ENSP00000358812	9606.ENSP00000268802	0	0	0	0	0.55	0.263	0.9	0.831	0.993
DHX37	UTP18	4438659	4433308	9606.ENSP00000311135	9606.ENSP00000225298	0	0	0	0	0.846	0.187	0.9	0.546	0.993
RPS6	TSR1	4445386	4437751	9606.ENSP00000369757	9606.ENSP00000301364	0	0	0	0	0.099	0.932	0.9	0	0.993
RPS6	IMP3	4445386	4439890	9606.ENSP00000369757	9606.ENSP00000326981	0	0	0	0	0.665	0.789	0.9	0.23	0.993
DCAF13	DDX49	4437420	4434008	9606.ENSP00000297579	9606.ENSP00000247003	0	0	0	0	0.925	0.053	0.9	0.221	0.993
TSR1	UTP15	4437751	4437338	9606.ENSP00000301364	9606.ENSP00000296792	0	0	0	0	0.914	0.087	0.9	0.221	0.993
CIRH1A	TSR1	4439903	4437751	9606.ENSP00000327179	9606.ENSP00000301364	0	0	0	0	0.907	0	0.9	0.326	0.993
BMS1	NOB1	4444407	4435851	9606.ENSP00000363642	9606.ENSP00000268802	0	0	0	0	0.841	0.064	0.9	0.625	0.993
WDR43	DDX49	4447102	4434008	9606.ENSP00000384302	9606.ENSP00000247003	0	0	0	0	0.917	0.05	0.9	0.221	0.993
HEATR1	DHX37	4442698	4438659	9606.ENSP00000355541	9606.ENSP00000311135	0	0	0	0	0.886	0.052	0.9	0.482	0.993
WDR46	DHX37	4444423	4438659	9606.ENSP00000363746	9606.ENSP00000311135	0	0	0	0	0.887	0	0.9	0.45	0.993
NOP56	DHX37	4445513	4438659	9606.ENSP00000370589	9606.ENSP00000311135	0	0	0	0	0.837	0.244	0.9	0.502	0.993
RPS3A	FBL	4441583	4433181	9606.ENSP00000346050	9606.ENSP00000221801	0	0	0	0	0.695	0.781	0.9	0.198	0.993
WDR3	DDX49	4438389	4434008	9606.ENSP00000308179	9606.ENSP00000247003	0	0	0	0	0.925	0.064	0.9	0.221	0.993
RCL1	NOB1	4445592	4435851	9606.ENSP00000371169	9606.ENSP00000268802	0.168	0	0	0	0.647	0	0.9	0.776	0.992
DHX37	WDR3	4438659	4438389	9606.ENSP00000311135	9606.ENSP00000308179	0	0	0	0	0.883	0	0.9	0.441	0.992
RCL1	KRR1	4445592	4433449	9606.ENSP00000371169	9606.ENSP00000229214	0	0	0	0	0.433	0.784	0.9	0.428	0.992
NOP14	RIOK2	4448347	4436521	9606.ENSP00000405068	9606.ENSP00000283109	0	0	0	0	0.81	0.05	0.9	0.64	0.992
DHX37	KRR1	4438659	4433449	9606.ENSP00000311135	9606.ENSP00000229214	0	0	0	0	0.725	0.355	0.9	0.637	0.992
NOP56	RPS3A	4445513	4441583	9606.ENSP00000370589	9606.ENSP00000346050	0	0	0	0	0.537	0.784	0.9	0.293	0.992
CIRH1A	NOB1	4439903	4435851	9606.ENSP00000327179	9606.ENSP00000268802	0	0	0	0	0.714	0.692	0.9	0.227	0.992

Gene 1	Gene 2	node1_string_internal_id	node2_string_internal_id	node1_external_id	node2_external_id	neighborhood_on_chromosome	gene_fusion	phylogenetic_cooccurrence	homology	coexpression	experimentally_determined_interaction	database_annotated	automated_textmining	combined_score
NOP56	RPS6	4445513	4445386	9606.ENSP00000370589	9606.ENSP00000369757	0	0	0	0	0.556	0.828	0.9	0.18	0.992
IMP3	NOL6	4439890	4437451	9606.ENSP00000326981	9606.ENSP00000297990	0	0	0	0	0.188	0.749	0.9	0.666	0.992
DIEXF	DDX49	4449004	4434008	9606.ENSP00000419005	9606.ENSP00000247003	0	0	0	0	0.85	0	0.9	0.563	0.992
NOP56	TSR1	4445513	4437751	9606.ENSP00000370589	9606.ENSP00000301364	0	0	0	0	0.893	0.112	0.9	0.335	0.992
NOP14	TSR1	4448347	4437751	9606.ENSP00000405068	9606.ENSP00000301364	0	0	0	0	0.902	0.131	0.9	0.221	0.992
WDR3	TSR1	4438389	4437751	9606.ENSP00000308179	9606.ENSP00000301364	0	0	0	0	0.905	0	0.9	0.294	0.992
DIEXF	HEATR1	4449004	4442698	9606.ENSP00000419005	9606.ENSP00000355541	0	0	0	0	0.856	0	0.9	0.497	0.992
DIEXF	DHX37	4449004	4438659	9606.ENSP00000419005	9606.ENSP00000311135	0	0	0	0	0.883	0.187	0.9	0.285	0.992
DIEXF	WDR43	4449004	4447102	9606.ENSP00000419005	9606.ENSP00000384302	0	0	0	0	0.895	0	0.9	0.339	0.992
TBL3	DIEXF	4450478	4449004	9606.ENSP00000454836	9606.ENSP00000419005	0	0	0	0	0.892	0	0.9	0.334	0.992
RPS3A	IMP3	4441583	4439890	9606.ENSP00000346050	9606.ENSP00000326981	0	0	0	0	0.657	0.769	0.9	0.208	0.992
RCL1	UTP11L	4445592	4444078	9606.ENSP00000371169	9606.ENSP00000362105	0	0	0	0	0.491	0.734	0.9	0.53	0.992
DIEXF	UTP18	4449004	4433308	9606.ENSP00000419005	9606.ENSP00000225298	0	0	0	0	0.856	0.144	0.9	0.497	0.992
DIEXF	UTP15	4449004	4437338	9606.ENSP00000419005	9606.ENSP00000296792	0	0	0	0	0.855	0	0.9	0.54	0.992
EMG1	MPHOSPH	4450965	4433887	9606.ENSP00000470560	9606.ENSP00000244230	0	0	0	0	0.287	0.793	0.9	0.487	0.991
DHX37	DCAF13	4438659	4437420	9606.ENSP00000311135	9606.ENSP00000297579	0	0	0	0	0.825	0	0.9	0.547	0.991
DIEXF	NOP14	4449004	4448347	9606.ENSP00000419005	9606.ENSP00000405068	0	0	0	0	0.889	0	0.9	0.318	0.991
DIEXF	WDR3	4449004	4438389	9606.ENSP00000419005	9606.ENSP00000308179	0	0	0	0	0.886	0	0.9	0.324	0.991
WDR46	TSR1	4444423	4437751	9606.ENSP00000363746	9606.ENSP00000301364	0	0	0	0	0.894	0	0.9	0.249	0.991
WDR75	DHX37	4438919	4438659	9606.ENSP00000314193	9606.ENSP00000311135	0	0	0	0	0.851	0	0.9	0.458	0.991
UTP11L	WDR75	4444078	4438919	9606.ENSP00000362105	9606.ENSP00000314193	0	0	0	0	0.439	0.779	0.9	0.392	0.991
WDR36	TSR1	4449235	4437751	9606.ENSP00000423067	9606.ENSP00000301364	0	0	0	0	0.902	0	0.9	0.221	0.991
DHX37	PWP2	4438659	4436922	9606.ENSP00000311135	9606.ENSP00000291576	0	0	0	0	0.802	0.264	0.9	0.479	0.991
DDX49	MPHOSPH	4434008	4433887	9606.ENSP00000247003	9606.ENSP00000244230	0	0	0	0	0.834	0.269	0.9	0.402	0.991
TBL3	TSR1	4450478	4437751	9606.ENSP00000454836	9606.ENSP00000301364	0	0	0	0	0.898	0	0.9	0.221	0.991
DIEXF	UTP6	4449004	4434930	9606.ENSP00000419005	9606.ENSP00000261708	0	0	0	0	0.884	0	0.9	0.336	0.991
EMG1	UTP3	4450965	4434386	9606.ENSP00000470560	9606.ENSP00000254803	0	0	0	0	0.378	0.784	0.9	0.425	0.991
DIEXF	BMS1	4449004	4444407	9606.ENSP00000419005	9606.ENSP00000363642	0	0	0	0	0.891	0	0.9	0.299	0.991
BMS1	DDX49	4444407	4434008	9606.ENSP00000363642	9606.ENSP00000247003	0	0	0	0	0.824	0.171	0.9	0.427	0.99
TSR1	NOP58	4437751	4435411	9606.ENSP00000301364	9606.ENSP00000264279	0	0	0	0	0.902	0	0.9	0.114	0.99
NOB1	IMP4	4435851	4434687	9606.ENSP00000268802	9606.ENSP00000259239	0	0	0	0	0.863	0	0.9	0.353	0.99
NOC4L	UTP3	4440054	4434386	9606.ENSP00000328854	9606.ENSP00000254803	0	0	0	0	0.468	0.788	0.9	0.221	0.99
TSR1	UTP18	4437751	4433308	9606.ENSP00000301364	9606.ENSP00000225298	0	0	0	0	0.875	0	0.9	0.328	0.99
UTP15	RIOK2	4437338	4436521	9606.ENSP00000296792	9606.ENSP00000283109	0	0	0	0	0.856	0	0.9	0.412	0.99
RPS3A	BYSL	4441583	4433499	9606.ENSP00000346050	9606.ENSP00000230340	0	0	0	0	0.21	0.877	0.9	0.122	0.99
DIEXF	TSR1	4449004	4437751	9606.ENSP00000419005	9606.ENSP00000301364	0	0	0	0	0.9	0	0.9	0.084	0.99
TSR1	DCAF13	4437751	4437420	9606.ENSP00000301364	9606.ENSP00000297579	0	0	0	0	0.884	0	0.9	0.239	0.99
DHX37	TSR1	4438659	4437751	9606.ENSP00000311135	9606.ENSP00000301364	0	0	0	0	0.892	0	0.9	0.229	0.99
TSR1	MPHOSPH	4437751	4433887	9606.ENSP00000301364	9606.ENSP00000244230	0	0	0	0	0.878	0	0.9	0.243	0.99
RPS3A	TSR1	4441583	4437751	9606.ENSP00000346050	9606.ENSP00000301364	0	0	0	0	0.063	0.902	0.9	0.075	0.99
TSR1	UTP6	4437751	4434930	9606.ENSP00000301364	9606.ENSP00000261708	0	0	0	0	0.88	0	0.9	0.246	0.99
TSR1	RRP9	4437751	4433566	9606.ENSP00000301364	9606.ENSP00000232888	0	0	0	0	0.884	0	0.9	0.221	0.99
WDR75	TSR1	4438919	4437751	9606.ENSP00000314193	9606.ENSP00000301364	0	0	0	0	0.883	0	0.9	0.221	0.99
NOL6	DDX49	4437451	4434008	9606.ENSP00000297990	9606.ENSP00000247003	0	0	0	0	0.598	0.692	0.9	0.318	0.99

Gene 1	Gene 2	node1_string_internal_id	node2_string_internal_id	node1_external_id	node2_external_id	neighborhood_on_chromosome	gene_fusion	phylogenetic_cooccurrence	homology	coexpression	experimentally_determined_interaction	database_annotated	automated_textmining	combined_score
PDCD11	IMP3	4443395	4439890	9606.ENSP00000358812	9606.ENSP00000326981	0.05	0	0	0	0.491	0.708	0.9	0.426	0.99
UTP11L	NOL6	4444078	4437451	9606.ENSP00000362105	9606.ENSP00000297990	0	0	0	0	0.427	0.651	0.9	0.562	0.99
BMS1	RIOK2	4444407	4436521	9606.ENSP00000363642	9606.ENSP00000283109	0	0	0	0	0.65	0.064	0.9	0.729	0.989
DHX37	UTP15	4438659	4437338	9606.ENSP00000311135	9606.ENSP00000296792	0	0	0	0	0.813	0.052	0.9	0.473	0.989
EMG1	PDCD11	4450965	4443395	9606.ENSP00000470560	9606.ENSP00000358812	0	0	0	0	0.319	0.706	0.9	0.531	0.989
RCL1	NOL6	4445592	4437451	9606.ENSP00000371169	9606.ENSP00000297990	0	0	0	0	0.26	0.651	0.9	0.657	0.989
RPS3A	RIOK2	4441583	4436521	9606.ENSP00000346050	9606.ENSP00000283109	0	0	0	0	0.078	0.883	0.9	0.161	0.989
UTP11L	PWP2	4444078	4436922	9606.ENSP00000362105	9606.ENSP00000291576	0	0	0	0	0.53	0.733	0.9	0.233	0.989
RCL1	DDX49	4445592	4434008	9606.ENSP00000371169	9606.ENSP00000247003	0	0	0	0	0.821	0	0.9	0.443	0.989
IMP3	DDX49	4439890	4434008	9606.ENSP00000326981	9606.ENSP00000247003	0.051	0	0	0	0.851	0	0.9	0.346	0.989
NOB1	UTP18	4435851	4433308	9606.ENSP00000268802	9606.ENSP00000225298	0	0	0	0	0.852	0	0.9	0.345	0.989
BMS1	IMP3	4444407	4439890	9606.ENSP00000363642	9606.ENSP00000326981	0	0	0	0	0.329	0.792	0.9	0.325	0.989
RPS3A	KRR1	4441583	4433449	9606.ENSP00000346050	9606.ENSP00000229214	0	0	0	0	0.226	0.856	0.9	0.05	0.988
RPS6	RIOK2	4445386	4436521	9606.ENSP00000369757	9606.ENSP00000283109	0	0	0	0	0.086	0.877	0.9	0.066	0.988
NOP14	LTV1	4448347	4442915	9606.ENSP00000405068	9606.ENSP00000356548	0	0	0	0	0.855	0	0.9	0.267	0.988
WDR36	DDX49	4449235	4434008	9606.ENSP00000423067	9606.ENSP00000247003	0	0	0	0	0.859	0	0.9	0.221	0.988
WDR43	DHX37	4447102	4438659	9606.ENSP00000384302	9606.ENSP00000311135	0	0	0	0	0.838	0.187	0.9	0.252	0.988
DIEXF	NOC4L	4449004	4440054	9606.ENSP00000419005	9606.ENSP00000328854	0	0	0	0	0.884	0.064	0.9	0	0.988
LTV1	NOP58	4442915	4435411	9606.ENSP00000356548	9606.ENSP00000264279	0	0	0	0	0.865	0.054	0.9	0.175	0.988
WDR46	NOB1	4444423	4435851	9606.ENSP00000363746	9606.ENSP00000268802	0	0	0	0	0.842	0	0.9	0.311	0.988
DIEXF	WDR75	4449004	4438919	9606.ENSP00000419005	9606.ENSP00000314193	0	0	0	0	0.836	0	0.9	0.374	0.988
LTV1	UTP3	4442915	4434386	9606.ENSP00000356548	9606.ENSP00000254803	0	0	0	0	0.856	0	0.9	0.231	0.988
UTP11L	LTV1	4444078	4442915	9606.ENSP00000362105	9606.ENSP00000356548	0	0	0	0	0.858	0	0.9	0.24	0.988
DIEXF	PNO1	4449004	4435289	9606.ENSP00000419005	9606.ENSP00000263657	0	0	0	0	0.882	0	0.9	0.1	0.988
WDR75	DDX49	4438919	4434008	9606.ENSP00000314193	9606.ENSP00000247003	0	0	0	0	0.857	0.05	0.9	0.221	0.988
TSR1	FBL	4437751	4433181	9606.ENSP00000301364	9606.ENSP00000221801	0	0	0	0	0.824	0.178	0.9	0.267	0.988
DIEXF	CIRH1A	4449004	4439903	9606.ENSP00000419005	9606.ENSP00000327179	0	0	0	0	0.885	0	0.9	0	0.988
UTP11L	FBL	4444078	4433181	9606.ENSP00000362105	9606.ENSP00000221801	0	0	0	0	0.295	0.788	0.9	0.323	0.988
NOP14	DDX49	4448347	4434008	9606.ENSP00000405068	9606.ENSP00000247003	0	0	0	0	0.848	0.082	0.9	0.221	0.987
RIOK2	IMP4	4436521	4434687	9606.ENSP00000283109	9606.ENSP00000259239	0	0	0	0	0.876	0	0.9	0.103	0.987
DIEXF	WDR46	4449004	4444423	9606.ENSP00000419005	9606.ENSP00000363746	0	0	0	0	0.882	0	0.9	0	0.987
DHX37	IMP4	4438659	4434687	9606.ENSP00000311135	9606.ENSP00000259239	0	0	0	0	0.757	0.18	0.9	0.443	0.987
DIEXF	BYSL	4449004	4433499	9606.ENSP00000419005	9606.ENSP00000230340	0	0	0	0	0.86	0	0.9	0.143	0.987
TSR1	PWP2	4437751	4436922	9606.ENSP00000301364	9606.ENSP00000291576	0	0	0	0	0.809	0	0.9	0.406	0.987
CIRH1A	DDX49	4439903	4434008	9606.ENSP00000327179	9606.ENSP00000247003	0	0	0	0	0.847	0	0.9	0.256	0.987
TSR1	UTP3	4437751	4434386	9606.ENSP00000301364	9606.ENSP00000254803	0	0	0	0	0.85	0	0.9	0.221	0.987
RIOK2	UTP18	4436521	4433308	9606.ENSP00000283109	9606.ENSP00000225298	0	0	0	0	0.768	0	0.9	0.486	0.987
TSR1	IMP4	4437751	4434687	9606.ENSP00000301364	9606.ENSP00000259239	0	0	0	0	0.856	0	0.9	0.221	0.987
RIOK2	UTP6	4436521	4434930	9606.ENSP00000283109	9606.ENSP00000261708	0	0	0	0	0.847	0.05	0.9	0.234	0.987
RPS3A	NOP58	4441583	4435411	9606.ENSP00000346050	9606.ENSP00000264279	0	0	0	0	0.328	0.821	0.9	0.099	0.987
DIEXF	KRR1	4449004	4433449	9606.ENSP00000419005	9606.ENSP00000229214	0	0	0	0	0.848	0	0.9	0.168	0.986
NOB1	UTP6	4435851	4434930	9606.ENSP00000268802	9606.ENSP00000261708	0	0	0	0	0.829	0	0.9	0.276	0.986
NOB1	MPHOSPH	4435851	4433887	9606.ENSP00000268802	9606.ENSP00000244230	0	0	0	0	0.82	0	0.9	0.303	0.986
LTV1	MPHOSPH	4442915	4433887	9606.ENSP00000356548	9606.ENSP00000244230	0	0	0	0	0.853	0	0.9	0.168	0.986

Gene 1	Gene 2	node1_string_internal_id	node2_string_internal_id	node1_external_id	node2_external_id	neighborhood_on_chromosome	gene_fusion	phylogenetic_cooccurrence	homology	coexpression	experimentally_determined_interaction	database_annotated	automated_textmining	combined_score
HEATR1	DDX49	4442698	4434008	9606.ENSP00000355541	9606.ENSP00000247003	0	0	0	0	0.837	0.064	0.9	0.221	0.986
PDCD11	LTV1	4443395	4442915	9606.ENSP00000358812	9606.ENSP00000356548	0	0	0	0	0.687	0	0.9	0.6	0.986
NOP14	NOB1	4448347	4435851	9606.ENSP00000405068	9606.ENSP00000268802	0	0	0	0	0.836	0	0.9	0.249	0.986
EMG1	DDX49	4450965	4434008	9606.ENSP00000470560	9606.ENSP00000247003	0	0	0	0	0.817	0	0.9	0.321	0.986
RPS6	NOP58	4445386	4435411	9606.ENSP00000369757	9606.ENSP00000264279	0	0	0	0	0.23	0.827	0.9	0.118	0.986
RPS6	KRR1	4445386	4433449	9606.ENSP00000369757	9606.ENSP00000229214	0	0	0	0	0.209	0.774	0.9	0.273	0.985
DHX37	NOB1	4438659	4435851	9606.ENSP00000311135	9606.ENSP00000268802	0	0	0	0	0.697	0.103	0.9	0.524	0.985
NOC4L	NOB1	4440054	4435851	9606.ENSP00000328854	9606.ENSP00000268802	0	0	0	0	0.524	0.064	0.9	0.721	0.985
RPS6	MPHOSPH	4445386	4433887	9606.ENSP00000369757	9606.ENSP00000244230	0	0	0	0	0.11	0.806	0.9	0.245	0.985
EMG1	RPS6	4450965	4445386	9606.ENSP00000470560	9606.ENSP00000369757	0	0	0	0	0.339	0.769	0.9	0.164	0.985
UTP11L	NOB1	4444078	4435851	9606.ENSP00000362105	9606.ENSP00000268802	0	0	0	0	0.807	0	0.9	0.308	0.985
TBL3	RIOK2	4450478	4436521	9606.ENSP00000454836	9606.ENSP00000283109	0	0	0	0	0.811	0	0.9	0.223	0.984
DIEXF	DCAF13	4449004	4437420	9606.ENSP00000419005	9606.ENSP00000297579	0	0	0	0	0.853	0	0.9	0	0.984
LTV1	IMP4	4442915	4434687	9606.ENSP00000356548	9606.ENSP00000259239	0	0	0	0	0.841	0	0.9	0.094	0.984
DIEXF	NOP58	4449004	4435411	9606.ENSP00000419005	9606.ENSP00000264279	0	0	0	0	0.835	0	0.9	0.132	0.984
LTV1	IMP3	4442915	4439890	9606.ENSP00000356548	9606.ENSP00000326981	0	0	0	0	0.461	0.713	0.9	0.11	0.984
PDCD11	DDX49	4443395	4434008	9606.ENSP00000358812	9606.ENSP00000247003	0	0	0	0	0.477	0.262	0.9	0.647	0.984
LTV1	WDR3	4442915	4438389	9606.ENSP00000356548	9606.ENSP00000308179	0	0	0	0	0.845	0	0.9	0.075	0.984
WDR36	RIOK2	4449235	4436521	9606.ENSP00000423067	9606.ENSP00000283109	0	0	0	0	0.838	0	0.9	0.143	0.984
WDR43	LTV1	4447102	4442915	9606.ENSP00000384302	9606.ENSP00000356548	0	0	0	0	0.849	0	0.9	0	0.984
RPS6	HEATR1	4445386	4442698	9606.ENSP00000369757	9606.ENSP00000355541	0	0	0	0	0.161	0.782	0.9	0.257	0.984
DIEXF	IMP3	4449004	4439890	9606.ENSP00000419005	9606.ENSP00000326981	0	0	0	0	0.836	0.118	0.9	0	0.984
WDR43	NOB1	4447102	4435851	9606.ENSP00000384302	9606.ENSP00000268802	0	0	0	0	0.839	0	0.9	0.115	0.984
DHX37	PNO1	4438659	4435289	9606.ENSP00000311135	9606.ENSP00000263657	0	0	0	0	0.774	0.18	0.9	0.239	0.984
RCL1	RIOK2	4445592	4436521	9606.ENSP00000371169	9606.ENSP00000283109	0	0	0	0	0.461	0	0.9	0.734	0.984
PWP2	NOB1	4436922	4435851	9606.ENSP00000291576	9606.ENSP00000268802	0	0	0	0	0.638	0	0.9	0.616	0.984
EMG1	TSR1	4450965	4437751	9606.ENSP00000470560	9606.ENSP00000301364	0	0	0	0	0.728	0	0.9	0.461	0.984
DIEXF	NOP56	4449004	4445513	9606.ENSP00000419005	9606.ENSP00000370589	0	0	0	0	0.837	0	0.9	0.097	0.984
DIEXF	PWP2	4449004	4436922	9606.ENSP00000419005	9606.ENSP00000291576	0	0	0	0	0.81	0	0.9	0.261	0.984
UTP11L	PDCD11	4444078	4443395	9606.ENSP00000362105	9606.ENSP00000358812	0	0	0	0	0.297	0.651	0.9	0.427	0.984
LTV1	UTP6	4442915	4434930	9606.ENSP00000356548	9606.ENSP00000261708	0	0	0	0	0.827	0	0.9	0.191	0.984
EMG1	RPS3A	4450965	4441583	9606.ENSP00000470560	9606.ENSP00000346050	0	0	0	0	0.362	0.769	0.9	0	0.983
NOB1	RRP9	4435851	4433566	9606.ENSP00000268802	9606.ENSP00000232888	0	0	0	0	0.829	0	0.9	0.115	0.983
HEATR1	RPS3A	4442698	4441583	9606.ENSP00000355541	9606.ENSP00000346050	0	0	0	0	0.256	0.782	0.9	0.088	0.983
RIOK2	DDX49	4436521	4434008	9606.ENSP00000283109	9606.ENSP00000247003	0	0	0	0	0.835	0.054	0.9	0.084	0.983
IMP3	TSR1	4439890	4437751	9606.ENSP00000326981	9606.ENSP00000301364	0	0	0	0	0.73	0.319	0.9	0.221	0.983
LTV1	WDR75	4442915	4438919	9606.ENSP00000356548	9606.ENSP00000314193	0	0	0	0	0.838	0	0.9	0	0.983
LTV1	DHX37	4442915	4438659	9606.ENSP00000356548	9606.ENSP00000311135	0	0	0	0	0.764	0	0.9	0.368	0.983
RIOK2	MPHOSPH	4436521	4433887	9606.ENSP00000283109	9606.ENSP00000244230	0	0	0	0	0.637	0	0.9	0.586	0.983
WDR3	NOB1	4438389	4435851	9606.ENSP00000308179	9606.ENSP00000268802	0	0	0	0	0.815	0	0.9	0.131	0.982
NOB1	KRR1	4435851	4433449	9606.ENSP00000268802	9606.ENSP00000229214	0	0	0	0	0.676	0.157	0.9	0.45	0.982
RIOK2	RRP9	4436521	4433566	9606.ENSP00000283109	9606.ENSP00000232888	0	0	0	0	0.83	0	0.9	0	0.982
RPS3A	RRP9	4441583	4433566	9606.ENSP00000346050	9606.ENSP00000232888	0	0	0	0	0.285	0.769	0.9	0	0.982
RPS6	UTP3	4445386	4434386	9606.ENSP00000369757	9606.ENSP00000254803	0	0	0	0	0.08	0.783	0.9	0.221	0.982

Gene 1	Gene 2	node1_string_internal_id	node2_string_internal_id	node1_external_id	node2_external_id	neighborhood_on_chromosome	gene_fusion	phylogenetic_cooccurrence	homology	coexpression	experimentally_determined_interaction	database_annotated	automated_textmining	combined_score
NOB1	DDX49	4435851	4434008	9606.ENSP00000268802	9606.ENSP00000247003	0	0	0	0	0.719	0.064	0.9	0.415	0.982
RPS6	UTP18	4445386	4433308	9606.ENSP00000369757	9606.ENSP00000225298	0	0	0	0	0.139	0.769	0.9	0.217	0.982
WDR46	LTV1	4444423	4442915	9606.ENSP00000363746	9606.ENSP00000356548	0	0	0	0	0.818	0	0.9	0.085	0.981
RPS6	PWP2	4445386	4436922	9606.ENSP00000369757	9606.ENSP00000291576	0	0	0	0	0.145	0.75	0.9	0.231	0.981
WDR46	RIOK2	4444423	4436521	9606.ENSP00000363746	9606.ENSP00000283109	0	0	0	0	0.814	0	0.9	0.083	0.981
RPS3A	NOB1	4441583	4435851	9606.ENSP00000346050	9606.ENSP00000268802	0	0	0	0	0.102	0.8	0.9	0.095	0.981
RCL1	DHX37	4445592	4438659	9606.ENSP00000371169	9606.ENSP00000311135	0	0	0	0	0.294	0.692	0.9	0.265	0.981
RPS6	NOB1	4445386	4435851	9606.ENSP00000369757	9606.ENSP00000268802	0	0	0	0	0.139	0.8	0.9	0.074	0.981
RPS6	WDR3	4445386	4438389	9606.ENSP00000369757	9606.ENSP00000308179	0	0	0	0	0.116	0.777	0.9	0.112	0.98
DIEXF	RIOK2	4449004	4436521	9606.ENSP00000419005	9606.ENSP00000283109	0	0	0	0	0.816	0	0.9	0	0.98
WDR3	RIOK2	4438389	4436521	9606.ENSP00000308179	9606.ENSP00000283109	0	0	0	0	0.813	0	0.9	0	0.98
WDR36	NOB1	4449235	4435851	9606.ENSP00000423067	9606.ENSP00000268802	0	0	0	0	0.651	0.41	0.9	0.165	0.98
DIEXF	UTP11L	4449004	4444078	9606.ENSP00000419005	9606.ENSP00000362105	0	0	0	0	0.813	0	0.9	0	0.98
NOC4L	RIOK2	4440054	4436521	9606.ENSP00000328854	9606.ENSP00000283109	0	0	0	0	0.763	0	0.9	0.25	0.98
NOP56	LTV1	4445513	4442915	9606.ENSP00000370589	9606.ENSP00000356548	0	0	0	0	0.753	0.064	0.9	0.273	0.98
LTV1	DCAF13	4442915	4437420	9606.ENSP00000356548	9606.ENSP00000297579	0	0	0	0	0.81	0	0.9	0.067	0.98
PDCD11	RIOK2	4443395	4436521	9606.ENSP00000358812	9606.ENSP00000283109	0	0	0	0	0.257	0	0.9	0.763	0.98
DDX49	KRR1	4434008	4433449	9606.ENSP00000247003	9606.ENSP00000229214	0	0	0	0	0.734	0.157	0.9	0.221	0.98
RPS6	RRP9	4445386	4433566	9606.ENSP00000369757	9606.ENSP00000232888	0	0	0	0	0.174	0.769	0.9	0.08	0.98
EMG1	NOB1	4450965	4435851	9606.ENSP00000470560	9606.ENSP00000268802	0	0	0	0	0.468	0	0.9	0.642	0.979
RPS3A	UTP18	4441583	4433308	9606.ENSP00000346050	9606.ENSP00000225298	0	0	0	0	0.185	0.769	0.9	0	0.979
NOP14	RPS6	4448347	4445386	9606.ENSP00000405068	9606.ENSP00000369757	0	0	0	0	0.064	0.784	0.9	0.085	0.979
RPS6	IMP4	4445386	4434687	9606.ENSP00000369757	9606.ENSP00000259239	0	0	0	0	0.078	0.788	0.9	0.064	0.979
RPS6	UTP6	4445386	4434930	9606.ENSP00000369757	9606.ENSP00000261708	0	0	0	0	0.168	0.769	0.9	0	0.979
RPS6	BMS1	4445386	4444407	9606.ENSP00000369757	9606.ENSP00000363642	0	0	0	0	0.093	0.782	0.9	0.062	0.979
RCL1	RPS6	4445592	4445386	9606.ENSP00000371169	9606.ENSP00000369757	0	0	0	0	0.129	0.777	0.9	0.069	0.979
TBL3	RPS3A	4450478	4441583	9606.ENSP00000454836	9606.ENSP00000346050	0	0	0	0	0.113	0.772	0.9	0	0.978
RCL1	RPS3A	4445592	4441583	9606.ENSP00000371169	9606.ENSP00000346050	0	0	0	0	0.122	0.769	0.9	0.062	0.978
NOB1	FBL	4435851	4433181	9606.ENSP00000268802	9606.ENSP00000221801	0	0	0	0	0.483	0.13	0.9	0.573	0.978
WDR36	RPS3A	4449235	4441583	9606.ENSP00000423067	9606.ENSP00000346050	0	0	0	0	0.136	0.769	0.9	0	0.978
WDR36	RPS6	4449235	4445386	9606.ENSP00000423067	9606.ENSP00000369757	0	0	0	0	0.087	0.777	0.9	0.078	0.978
NOP56	DDX49	4445513	4434008	9606.ENSP00000370589	9606.ENSP00000247003	0.05	0	0	0	0.655	0.269	0.9	0.227	0.978
TBL3	RPS6	4450478	4445386	9606.ENSP00000454836	9606.ENSP00000369757	0	0	0	0	0.097	0.784	0.9	0	0.978
RPS6	UTP11L	4445386	4444078	9606.ENSP00000369757	9606.ENSP00000362105	0	0	0	0	0.132	0.764	0.9	0.067	0.978
NOP58	DDX49	4435411	4434008	9606.ENSP00000264279	9606.ENSP00000247003	0.05	0	0	0	0.663	0.269	0.9	0.21	0.978
RPS3A	WDR3	4441583	4438389	9606.ENSP00000346050	9606.ENSP00000308179	0	0	0	0	0.113	0.769	0.9	0	0.977
BMS1	RPS3A	4444407	4441583	9606.ENSP00000363642	9606.ENSP00000346050	0	0	0	0	0.084	0.769	0.9	0	0.977
DIEXF	FBL	4449004	4433181	9606.ENSP00000419005	9606.ENSP00000221801	0	0	0	0	0.324	0.411	0.9	0.503	0.977
RPS6	CIRH1A	4445386	4439903	9606.ENSP00000369757	9606.ENSP00000327179	0	0	0	0	0.07	0.769	0.9	0.085	0.977
RPS6	WDR46	4445386	4444423	9606.ENSP00000369757	9606.ENSP00000363746	0	0	0	0	0.114	0.769	0.9	0	0.977
RPS6	DCAF13	4445386	4437420	9606.ENSP00000369757	9606.ENSP00000297579	0	0	0	0	0.117	0.769	0.9	0	0.977
RPS3A	MPHOSPH	4441583	4433887	9606.ENSP00000346050	9606.ENSP00000244230	0	0	0	0	0.097	0.769	0.9	0	0.977
RPS6	UTP15	4445386	4437338	9606.ENSP00000369757	9606.ENSP00000296792	0	0	0	0	0.078	0.77	0.9	0	0.976
UTP15	NOB1	4437338	4435851	9606.ENSP00000296792	9606.ENSP00000268802	0	0	0	0	0.636	0	0.9	0.396	0.976

Gene 1	Gene 2	node1_string_internal_id	node2_string_internal_id	node1_external_id	node2_external_id	neighborhood_on_chromosome	gene_fusion	phylogenetic_cooccurrence	homology	coexpression	experimentally_determined_interaction	database_annotated	automated_textmining	combined_score
RPS3A	PWP2	4441583	4436922	9606.ENSP00000346050	9606.ENSP00000291576	0	0	0	0	0.095	0.734	0.9	0.128	0.976
RPS6	WDR75	4445386	4438919	9606.ENSP00000369757	9606.ENSP00000314193	0	0	0	0	0.071	0.769	0.9	0	0.976
RPS3A	IMP4	4441583	4434687	9606.ENSP00000346050	9606.ENSP00000259239	0	0	0	0	0.082	0.769	0.9	0	0.976
LTV1	UTP18	4442915	4433308	9606.ENSP00000356548	9606.ENSP00000225298	0	0	0	0	0.654	0	0.9	0.382	0.976
RPS3A	UTP6	4441583	4434930	9606.ENSP00000346050	9606.ENSP00000261708	0	0	0	0	0.119	0.737	0.9	0	0.974
UTP11L	DHX37	4444078	4438659	9606.ENSP00000362105	9606.ENSP00000311135	0	0	0	0	0.683	0	0.9	0.256	0.974
NOB1	UTP3	4435851	4434386	9606.ENSP00000268802	9606.ENSP00000254803	0	0	0	0	0.671	0	0.9	0.274	0.974
WDR46	RPS3A	4444423	4441583	9606.ENSP00000363746	9606.ENSP00000346050	0	0	0	0	0.138	0.723	0.9	0	0.974
NOP56	NOB1	4445513	4435851	9606.ENSP00000370589	9606.ENSP00000268802	0	0	0	0	0.668	0	0.9	0.269	0.973
PWP2	RIOK2	4436922	4436521	9606.ENSP00000291576	9606.ENSP00000283109	0	0	0	0	0.471	0	0.9	0.527	0.972
NOP14	RPS3A	4448347	4441583	9606.ENSP00000405068	9606.ENSP00000346050	0	0	0	0	0.067	0.73	0.9	0	0.972
NOL6	NOB1	4437451	4435851	9606.ENSP00000297990	9606.ENSP00000268802	0	0	0	0	0.284	0	0.9	0.649	0.972
WDR43	RIOK2	4447102	4436521	9606.ENSP00000384302	9606.ENSP00000283109	0	0	0	0	0.722	0.05	0.9	0	0.971
DCAF13	RIOK2	4437420	4436521	9606.ENSP00000297579	9606.ENSP00000283109	0	0	0	0	0.716	0	0.9	0.065	0.971
TBL3	NOB1	4450478	4435851	9606.ENSP00000454836	9606.ENSP00000268802	0	0	0	0	0.682	0	0.9	0.188	0.971
DDX49	FBL	4434008	4433181	9606.ENSP00000247003	9606.ENSP00000221801	0	0.004	0	0	0.448	0.157	0.9	0.463	0.971
RPS3A	CIRH1A	4441583	4439903	9606.ENSP00000346050	9606.ENSP00000327179	0	0	0	0	0.154	0.681	0.9	0	0.97
LTV1	RRP9	4442915	4433566	9606.ENSP00000356548	9606.ENSP00000232888	0	0	0	0	0.664	0	0.9	0.198	0.97
NOB1	NOP58	4435851	4435411	9606.ENSP00000268802	9606.ENSP00000264279	0	0	0	0	0.642	0	0.9	0.253	0.97
LTV1	UTP15	4442915	4437338	9606.ENSP00000356548	9606.ENSP00000296792	0	0	0	0	0.643	0	0.9	0.237	0.97
WDR75	RIOK2	4438919	4436521	9606.ENSP00000314193	9606.ENSP00000283109	0	0	0	0	0.7	0	0.9	0.058	0.969
TBL3	LTV1	4450478	4442915	9606.ENSP00000454836	9606.ENSP00000356548	0	0	0	0	0.677	0	0.9	0.145	0.969
EMG1	RIOK2	4450965	4436521	9606.ENSP00000470560	9606.ENSP00000283109	0	0	0	0	0.482	0	0.9	0.453	0.969
DIEXF	RCL1	4449004	4445592	9606.ENSP00000419005	9606.ENSP00000371169	0	0	0	0	0.566	0	0.9	0.36	0.969
EMG1	DIEXF	4450965	4449004	9606.ENSP00000470560	9606.ENSP00000419005	0	0	0	0	0.694	0.05	0.9	0.048	0.968
CIRH1A	RIOK2	4439903	4436521	9606.ENSP00000327179	9606.ENSP00000283109	0	0	0	0	0.581	0	0.9	0.278	0.967
DIEXF	NOB1	4449004	4435851	9606.ENSP00000419005	9606.ENSP00000268802	0	0	0	0	0.685	0	0.9	0.054	0.967
UTP11L	RIOK2	4444078	4436521	9606.ENSP00000362105	9606.ENSP00000283109	0	0	0	0	0.599	0	0.9	0.259	0.967
LTV1	CIRH1A	4442915	4439903	9606.ENSP00000356548	9606.ENSP00000327179	0	0	0	0	0.673	0	0.9	0.082	0.967
RPS3A	UTP3	4441583	4434386	9606.ENSP00000346050	9606.ENSP00000254803	0	0	0	0	0.103	0.651	0.9	0.085	0.967
DIEXF	LTV1	4449004	4442915	9606.ENSP00000419005	9606.ENSP00000356548	0	0	0	0	0.674	0	0.9	0	0.966
DCAF13	NOB1	4437420	4435851	9606.ENSP00000297579	9606.ENSP00000268802	0	0	0	0	0.641	0	0.9	0.129	0.966
LTV1	NOC4L	4442915	4440054	9606.ENSP00000356548	9606.ENSP00000328854	0	0	0	0	0.255	0	0.9	0.587	0.966
RPS6	NOC4L	4445386	4440054	9606.ENSP00000369757	9606.ENSP00000328854	0	0	0	0	0.27	0.582	0.9	0	0.966
WDR75	NOB1	4438919	4435851	9606.ENSP00000314193	9606.ENSP00000268802	0	0	0	0	0.647	0	0.9	0.115	0.966
RPS3A	DCAF13	4441583	4437420	9606.ENSP00000346050	9606.ENSP00000297579	0	0	0	0	0.112	0.651	0.9	0	0.966
LTV1	PWP2	4442915	4436922	9606.ENSP00000356548	9606.ENSP00000291576	0	0	0	0	0.5	0	0.9	0.379	0.966
RPS3A	WDR75	4441583	4438919	9606.ENSP00000346050	9606.ENSP00000314193	0	0	0	0	0.097	0.651	0.9	0	0.965
RPS3A	UTP15	4441583	4437338	9606.ENSP00000346050	9606.ENSP00000296792	0	0	0	0	0.068	0.651	0.9	0	0.964
UTP11L	RPS3A	4444078	4441583	9606.ENSP00000362105	9606.ENSP00000346050	0	0	0	0	0.106	0.635	0.9	0	0.964
IMP3	DHX37	4439890	4438659	9606.ENSP00000326981	9606.ENSP00000311135	0	0	0	0	0.339	0.167	0.9	0.426	0.964
EMG1	DHX37	4450965	4438659	9606.ENSP00000470560	9606.ENSP00000311135	0	0	0	0	0.247	0	0.9	0.562	0.964
DHX37	FBL	4438659	4433181	9606.ENSP00000311135	9606.ENSP00000221801	0	0	0	0	0.228	0.18	0.9	0.484	0.963
UTP11L	TSR1	4444078	4437751	9606.ENSP00000362105	9606.ENSP00000301364	0	0	0	0	0.57	0	0.9	0.221	0.963

Gene 1	Gene 2	node1_string_internal_id	node2_string_internal_id	node1_external_id	node2_external_id	neighborhood_on_chromosome	gene_fusion	phylogenetic_cooccurrence	homology	coexpression	experimentally_determined_interaction	database_annotated	automated_textmining	combined_score
WDR36	LTV1	4449235	4442915	9606.ENSP00000423067	9606.ENSP00000356548	0	0	0	0	0.639	0	0.9	0.074	0.963
LTV1	NOL6	4442915	4437451	9606.ENSP00000356548	9606.ENSP00000297990	0	0	0	0	0.317	0	0.9	0.474	0.96
IMP3	RIOK2	4439890	4436521	9606.ENSP00000326981	9606.ENSP00000283109	0	0	0	0	0.429	0.31	0.9	0.126	0.96
RIOK2	FBL	4436521	4433181	9606.ENSP00000283109	9606.ENSP00000221801	0	0	0	0	0.461	0	0.9	0.291	0.958
HEATR1	RIOK2	4442698	4436521	9606.ENSP00000355541	9606.ENSP00000283109	0	0	0	0	0.435	0	0.9	0.269	0.955
LTV1	DDX49	4442915	4434008	9606.ENSP00000356548	9606.ENSP00000247003	0	0	0	0	0.521	0.05	0.9	0.073	0.952
WDR43	RPS6	4447102	4445386	9606.ENSP00000384302	9606.ENSP00000369757	0	0	0	0	0.218	0.434	0.9	0	0.951
RCL1	LTV1	4445592	4442915	9606.ENSP00000371169	9606.ENSP00000356548	0	0	0	0	0.193	0	0.9	0.437	0.95
RPS6	NOL6	4445386	4437451	9606.ENSP00000369757	9606.ENSP00000297990	0	0	0	0	0.078	0.449	0.9	0.128	0.949
IMP3	NOB1	4439890	4435851	9606.ENSP00000326981	9606.ENSP00000268802	0	0	0	0	0.332	0.068	0.9	0.269	0.948
NOL6	RIOK2	4437451	4436521	9606.ENSP00000297990	9606.ENSP00000283109	0	0	0	0	0.155	0.064	0.9	0.418	0.947
DHX37	RIOK2	4438659	4436521	9606.ENSP00000311135	9606.ENSP00000283109	0	0	0	0	0.462	0	0.9	0.106	0.947
RPS6	PDCD11	4445386	4443395	9606.ENSP00000369757	9606.ENSP00000358812	0	0	0	0	0.129	0.428	0.9	0.074	0.947
PDCD11	RPS3A	4443395	4441583	9606.ENSP00000358812	9606.ENSP00000346050	0	0	0	0	0.129	0.414	0.9	0.093	0.947
RIOK2	NOP58	4436521	4435411	9606.ENSP00000283109	9606.ENSP00000264279	0	0	0	0	0.383	0	0.9	0.198	0.946
RPS3A	NOL6	4441583	4437451	9606.ENSP00000346050	9606.ENSP00000297990	0	0	0	0	0.124	0.414	0.9	0.069	0.945
LTV1	FBL	4442915	4433181	9606.ENSP00000356548	9606.ENSP00000221801	0	0	0	0	0.249	0.099	0.9	0.284	0.945
RIOK2	UTP3	4436521	4434386	9606.ENSP00000283109	9606.ENSP00000254803	0	0	0	0	0.386	0	0.9	0.162	0.944
NOP56	RIOK2	4445513	4436521	9606.ENSP00000370589	9606.ENSP00000283109	0	0	0	0	0.46	0	0.9	0.048	0.944
RPS3A	DHX37	4441583	4438659	9606.ENSP00000346050	9606.ENSP00000311135	0	0	0	0	0.106	0.416	0.9	0	0.943
EMG1	LTV1	4450965	4442915	9606.ENSP00000470560	9606.ENSP00000356548	0	0	0	0	0.262	0	0.9	0.27	0.941
HEATR1	NOB1	4442698	4435851	9606.ENSP00000355541	9606.ENSP00000268802	0	0	0	0	0.289	0	0.9	0.228	0.94
LTV1	HEATR1	4442915	4442698	9606.ENSP00000356548	9606.ENSP00000355541	0	0	0	0	0.378	0	0.9	0.084	0.938
WDR43	RPS3A	4447102	4441583	9606.ENSP00000384302	9606.ENSP00000346050	0	0	0	0	0.354	0	0.9	0	0.932
RPS3A	NOC4L	4441583	4440054	9606.ENSP00000346050	9606.ENSP00000328854	0	0	0	0	0.201	0.064	0.9	0	0.918
RPS6	DDX49	4445386	4434008	9606.ENSP00000369757	9606.ENSP00000247003	0	0	0	0	0.095	0.05	0.9	0.084	0.91
RPS6	DHX37	4445386	4438659	9606.ENSP00000369757	9606.ENSP00000311135	0	0	0	0	0.072	0.102	0.9	0.049	0.91
DIEXF	RPS6	4449004	4445386	9606.ENSP00000419005	9606.ENSP00000369757	0	0	0	0	0.081	0.075	0.9	0.067	0.91
RPS3A	DDX49	4441583	4434008	9606.ENSP00000346050	9606.ENSP00000247003	0	0	0	0	0.063	0.08	0.9	0.049	0.907
DIEXF	RPS3A	4449004	4441583	9606.ENSP00000419005	9606.ENSP00000346050	0	0	0	0	0	0	0.9	0	0.9
