# Supplementary material for: Overexpression of DDX49 in prostate cancer is associated with poor prognosis
Source: BMC Urol. 2023 Apr 27;23:66. doi: 10.1186/s12894-023-01251-4 (PMC10134639; doi:10.1186/s12894-023-01251-4)
Supplement: Supplementary file 2 — Additional file 2: Table S2. The GO and KEGG enrichment results of DDX49 related genes. [file 12894_2023_1251_MOESM2_ESM.pdf]

Table S2. The GO and KEGG enrichment results of DDX49 related genes.

ONTOLOGY	ID	Description	GeneRatio	BgRatio	pvalue	p.adjust	qvalue	geneID	Count
BP	GO:0006364	rRNA processing	35/37	205/18493	1.14E-67	2.15E-65	1.56E-65	FBL/UTP18/KRR1/BYSL/RRP9/MPHOSPH10/DDX49/UTP3/IMP4/UTP6/NOP58/NOB1/RIOK2/PWP2/UTP15/DCAF13/NOL6/TSR1/WDR3/DHX37/WDR75/IMP3/NOC4L/HEATR1/PDCD11/BMS1/WDR46/RPS6/NOP56/RCL1/WDR43/NOP14/WDR36/TBL3/EMG1	35
BP	GO:0042254	ribosome biogenesis	36/37	282/18493	1.45E-65	1.36E-63	9.90E-64	FBL/UTP18/KRR1/BYSL/RRP9/MPHOSPH10/DDX49/UTP3/IMP4/UTP6/NOP58/NOB1/RIOK2/PWP2/UTP15/DCAF13/NOL6/TSR1/WDR3/DHX37/WDR75/IMP3/NOC4L/HEATR1/LTV1/PDCD11/BMS1/WDR46/RPS6/NOP56/RCL1/WDR43/NOP14/WDR36/TBL3/	36
BP	GO:0016072	rRNA metabolic process	35/37	240/18493	4.55E-65	2.85E-63	2.07E-63	FBL/UTP18/KRR1/BYSL/RRP9/MPHOSPH10/DDX49/UTP3/IMP4/UTP6/NOP58/NOB1/RIOK2/PWP2/UTP15/DCAF13/NOL6/TSR1/WDR3/DHX37/WDR75/IMP3/NOC4L/HEATR1/PDCD11/BMS1/WDR46/RPS6/NOP56/RCL1/WDR43/NOP14/WDR36/TBL3/EMG1	35
BP	GO:0034470	ncRNA processing	35/37	376/18493	7.92E-58	3.72E-56	2.71E-56	FBL/UTP18/KRR1/BYSL/RRP9/MPHOSPH10/DDX49/UTP3/IMP4/UTP6/NOP58/NOB1/RIOK2/PWP2/UTP15/DCAF13/NOL6/TSR1/WDR3/DHX37/WDR75/IMP3/NOC4L/HEATR1/PDCD11/BMS1/WDR46/RPS6/NOP56/RCL1/WDR43/NOP14/WDR36/TBL3/EMG1	35
BP	GO:0022613	ribonucleoprotein complex biogenesis	36/37	485/18493	1.17E-56	4.40E-55	3.21E-55	FBL/UTP18/KRR1/BYSL/RRP9/MPHOSPH10/DDX49/UTP3/IMP4/UTP6/NOP58/NOB1/RIOK2/PWP2/UTP15/DCAF13/NOL6/TSR1/WDR3/DHX37/WDR75/IMP3/NOC4L/HEATR1/LTV1/PDCD11/BMS1/WDR46/RPS6/NOP56/RCL1/WDR43/NOP14/WDR36/TBL3/	36
BP	GO:0042274	ribosomal small subunit biogenesis	18/37	63/18493	4.42E-36	1.38E-34	1.01E-34	BYSL/UTP3/UTP6/NOB1/RIOK2/PWP2/DCAF13/TSR1/DHX37/HEATR1/LTV1/BMS1/WDR46/RPS6/RCL1/NOP14/TBL3/EMG1	18

ONTOLOGY	ID	Description	GeneRatio	BgRatio	pvalue	p.adjust	qvalue	geneID	Count
BP	GO:0030490	maturation of SSU-rRNA	15/37	43/18493	1.79E-31	4.80E-30	3.49E-30	BYSL/UTP3/UTP6/NOB1/RIOK2/PWP2/DCAF13/TSR1/DHX37/HEATR1/BMS1/WDR46/RCL1/NOP14/TBL3	15
BP	GO:0000462	maturation of SSU-rRNA from tricistronic rRNA transcript (SSU-rRNA, 5.8S rRNA, LSU-rRNA)	13/37	33/18493	4.21E-28	9.90E-27	7.21E-27	BYSL/UTP3/UTP6/PWP2/DCAF13/TSR1/DHX37/HEATR1/BMS1/WDR46/RCL1/NOP14/TBL3	13
BP	GO:2000232	regulation of rRNA processing	5/37	12/18493	1.90E-11	3.96E-10	2.88E-10	RIOK2/UTP15/WDR75/HEATR1/WDR43	5
BP	GO:0090069	regulation of ribosome biogenesis	5/37	14/18493	4.78E-11	8.99E-10	6.54E-10	RIOK2/UTP15/WDR75/HEATR1/WDR43	5
BP	GO:0045943	positive regulation of transcription by RNA	4/37	22/18493	9.67E-08	1.65E-06	1.20E-06	UTP15/WDR75/HEATR1/WDR43	4
BP	GO:0006356	regulation of transcription by RNA polymerase I	4/37	32/18493	4.68E-07	7.34E-06	5.34E-06	UTP15/WDR75/HEATR1/WDR43	4
BP	GO:0006360	transcription by RNA polymerase I	4/37	63/18493	7.42E-06	0.000107	7.81E-05	UTP15/WDR75/HEATR1/WDR43	4
BP	GO:0000469	cleavage involved in rRNA processing	3/37	21/18493	9.57E-06	0.000128	9.35E-05	NOB1/RCL1/NOP14	3
BP	GO:0006403	RNA localization	5/37	231/18493	9.16E-05	0.001148	0.000836	FBL/NOP58/RIOK2/NOL6/LTV1	5
BP	GO:0000478	endonucleolytic cleavage involved in rRNA	2/37	11/18493	0.000212	0.002342	0.001705	RCL1/NOP14	2
BP	GO:0000479	endonucleolytic cleavage of tricistronic rRNA	2/37	11/18493	0.000212	0.002342	0.001705	RCL1/NOP14	2
BP	GO:0000054	ribosomal subunit export from nucleus	2/37	15/18493	0.000402	0.003981	0.002898	RIOK2/LTV1	2
BP	GO:0033750	ribosome localization	2/37	15/18493	0.000402	0.003981	0.002898	RIOK2/LTV1	2
BP	GO:0090502	RNA phosphodiester bond hydrolysis,	3/37	76/18493	0.000469	0.004406	0.003207	NOB1/RCL1/NOP14	3
BP	GO:0071428	rRNA-containing ribonucleoprotein complex	2/37	17/18493	0.00052	0.004653	0.003387	RIOK2/LTV1	2
BP	GO:0000466	maturation of 5.8S rRNA from tricistronic rRNA	2/37	21/18493	0.000799	0.006824	0.004967	RCL1/NOP14	2
BP	GO:0031167	rRNA methylation	2/37	26/18493	0.001228	0.010039	0.007307	FBL/EMG1	2
BP	GO:0000460	maturation of 5.8S rRNA	2/37	27/18493	0.001325	0.010377	0.007553	RCL1/NOP14	2
BP	GO:0071426	ribonucleoprotein complex export from nucleus	3/37	129/18493	0.002167	0.01548	0.011267	RIOK2/NOL6/LTV1	3
BP	GO:0071166	ribonucleoprotein complex localization	3/37	130/18493	0.002215	0.01548	0.011267	RIOK2/NOL6/LTV1	3
BP	GO:0000154	rRNA modification	2/37	35/18493	0.002223	0.01548	0.011267	FBL/EMG1	2
BP	GO:0006405	RNA export from nucleus	3/37	137/18493	0.002571	0.017259	0.012563	RIOK2/NOL6/LTV1	3
BP	GO:0090501	RNA phosphodiester bond hydrolysis	3/37	152/18493	0.003447	0.022345	0.016264	NOB1/RCL1/NOP14	3
BP	GO:0006611	protein export from nucleus	3/37	180/18493	0.005526	0.034629	0.025206	RIOK2/NOL6/LTV1	3
BP	GO:0051168	nuclear export	3/37	194/18493	0.006795	0.038109	0.027739	RIOK2/NOL6/LTV1	3
BP	GO:0001824	blastocyst development	2/37	62/18493	0.00683	0.038109	0.027739	BYSL/EMG1	2
BP	GO:0050657	nucleic acid transport	3/37	195/18493	0.006892	0.038109	0.027739	RIOK2/NOL6/LTV1	3
BP	GO:0050658	RNA transport	3/37	195/18493	0.006892	0.038109	0.027739	RIOK2/NOL6/LTV1	3
BP	GO:0051236	establishment of RNA localization	3/37	198/18493	0.007187	0.038606	0.028101	RIOK2/NOL6/LTV1	3
CC	GO:0030684	preribosome	29/38	73/19659	8.42E-66	2.78E-64	1.86E-64	FBL/UTP18/KRR1/BYSL/RRP9/MPHOSPH10/UTP3/IMP4/UTP6/NOP58/NOB1/RIOK2/PWP2/DCAF13/NOL6/TSR1/WDR3/IMP3/NOC4L/HEATR1/LTV1/PDCD11/BMS1/WDR46/NOP56/NOP14/WDR36/TBL3/EMG1	29

ONTOLOGY	ID	Description	GeneRatio	BgRatio	pvalue	p.adjust	qvalue	geneID	Count
CC	GO:0032040	small-subunit processome	23/38	37/19659	4.34E-57	7.15E-56	4.79E-56	FBL/UTP18/KRR1/RRP9/MPHOSPH10/UTP3/IMP4/UTP6/NOP58/PWP2/DCAF13/NOL6/WDR3/IMP3/NOC4L/HEATR1/PDCD11/WDR46/NOP56/NOP14/WDR36/TBL3/EMG1	23
CC	GO:0030686	90S preribosome	14/38	29/19659	5.01E-32	5.52E-31	3.69E-31	UTP18/MPHOSPH10/IMP4/UTP6/PWP2/NOL6/WDR3/IMP3/NOC4L/HEATR1/BMS1/NOP14/WDR36/TBL3	14
CC	GO:0044452	nucleolar part	17/38	180/19659	2.52E-25	2.08E-24	1.39E-24	FBL/UTP18/RRP9/MPHOSPH10/IMP4/UTP6/NOP58/PWP2/UTP15/NOL6/WDR3/IMP3/HEATR1/NOP56/WDR43/WDR36/TBL3	17
CC	GO:0030688	preribosome, small subunit precursor	7/38	13/19659	9.55E-17	6.30E-16	4.22E-16	BYSL/NOB1/RIOK2/TSR1/NOC4L/LTV1/NOP14	7
CC	GO:0005732	small nucleolar ribonucleoprotein complex	5/38	28/19659	1.95E-09	1.07E-08	7.20E-09	FBL/RRP9/MPHOSPH10/NOP58/NOP56	5
CC	GO:0001650	fibrillar center	7/38	134/19659	6.18E-09	2.91E-08	1.95E-08	FBL/IMP4/NOP58/UTP15/HEATR1/NOP56/WDR43	7
CC	GO:0022627	cytosolic small ribosomal subunit	2/38	45/19659	0.003418	0.014099	0.009444	RPS3A/RPS6	2
CC	GO:0015935	small ribosomal subunit	2/38	73/19659	0.00877	0.030485	0.020421	RPS3A/RPS6	2
CC	GO:0015030	Cajal body	2/38	75/19659	0.009238	0.030485	0.020421	FBL/NOP58	2
MF	GO:0030515	snoRNA binding	14/38	31/17632	7.83E-31	3.92E-29	2.56E-29	BYSL/RRP9/IMP4/UTP6/NOP58/PWP2/TSR1/WDR3/IMP3/HEATR1/BMS1/NOP56/NOP14/TBL3	14
MF	GO:0001094	TFIID-class transcription factor binding	2/38	11/17632	0.000246	0.006131	0.004001	FBL/NOP58	2
MF	GO:0019843	rRNA binding	3/38	65/17632	0.000368	0.006131	0.004001	IMP4/IMP3/EMG1	3
MF	GO:0001091	RNA polymerase II basal transcription factor	2/38	20/17632	0.000839	0.006611	0.004315	FBL/NOP58	2
MF	GO:0140098	catalytic activity, acting on RNA	5/38	352/17632	0.0009	0.006611	0.004315	FBL/NOB1/DHX37/RCL1/EMG1	5
MF	GO:0008649	rRNA methyltransferase activity	2/38	21/17632	0.000926	0.006611	0.004315	FBL/EMG1	2
MF	GO:0140102	catalytic activity, acting on a rRNA	2/38	21/17632	0.000926	0.006611	0.004315	FBL/EMG1	2
MF	GO:0004521	endoribonuclease activity	2/38	63/17632	0.008131	0.042821	0.027946	NOB1/RCL1	2
MF	GO:0008173	RNA methyltransferase activity	2/38	63/17632	0.008131	0.042821	0.027946	FBL/EMG1	2
MF	GO:0001098	basal transcription machinery binding	2/38	68/17632	0.009421	0.042821	0.027946	FBL/NOP58	2
MF	GO:0001099	basal RNA polymerase II transcription	2/38	68/17632	0.009421	0.042821	0.027946	FBL/NOP58	2
KEGG	hsa03008	Ribosome biogenesis in eukaryotes	22/24	105/7882	1.43E-40	1.43E-39	1.36E-39	RCL1/MPHOSPH10/EMG1/NOP56/TBL3/WDR3/WDR36/FBL/WDR43/NOB1/UTP18/NOP58/HEATR1/IMP3/RIOK2/UTP6/PWP2/NOL6/WDR75/UTP15/IMP4/BMS1	22
